# Floxed *Il1rl2* Locus with mCherry Reporter Element Reveals Distinct Expression Patterns of the IL-36 Receptor in Barrier Tissues

**DOI:** 10.3390/cells13090787

**Published:** 2024-05-06

**Authors:** Nopprarat Tongmuang, Kathy Q. Cai, Jiahui An, Mariah Novy, Liselotte E. Jensen

**Affiliations:** 1 Department of Microbiology, Immunology and Inflammation, Temple University Lewis Katz School of Medicine, Philadelphia, PA 19140, USA; 2 Center for Inflammation and Lung Research, Temple University Lewis Katz School of Medicine, Philadelphia, PA 19140, USA; 3 Histopathology Facility, Fox Chase Cancer Center, Temple Health, Philadelphia, PA 19111, USA; 4 Cancer Signaling and Microenvironment, Fox Chase Cancer Center, Temple Health, Philadelphia, PA 19111, USA

**Keywords:** barrier tissue, epithelial cells, epithelium, IL-1RL2, *Il1rl2*, IL-36, IL-36RA, IL-38, interleukin, leukocytes

## Abstract

IL-36 cytokines are emerging as beneficial in immunity against pathogens and cancers but can also be detrimental when dysregulated in autoimmune and autoinflammatory conditions. Interest in targeting IL-36 activity for therapeutic purposes is rapidly growing, yet many unknowns about the functions of these cytokines remain. Thus, the availability of robust research tools is essential for both fundamental basic science and pre-clinical studies to fully access outcomes of any manipulation of the system. For this purpose, a floxed *Il1rl2*, the gene encoding the IL-36 receptor, mouse strain was developed to facilitate the generation of conditional knockout mice. The targeted locus was engineered to contain an inverted mCherry reporter sequence that upon Cre-mediated recombination will be flipped and expressed under the control of the endogenous *Il1rl2* promoter. This feature can be used to confirm knockout in individual cells but also as a reporter to determine which cells express the IL-36 receptor IL-1RL2. The locus was confirmed to function as intended and further used to demonstrate the expression of IL-1RL2 in barrier tissues. *Il1rl2* expression was detected in leukocytes in all barrier tissues. Interestingly, strong expression was observed in epithelial cells at locations in direct contact with the environment such as the skin, oral mucosa, the esophagus, and the upper airways, but almost absent from epithelial cells at more inward facing sites, including lung alveoli, the small intestine, and the colon. These findings suggest specialized functions of IL-1RL2 in outward facing epithelial tissues and cells. The generated mouse model should prove valuable in defining such functions and may also facilitate basic and translational research.

## 1. Introduction

The IL-36 cytokines initiate immune responses by promoting the expression of immune-related genes in cells bearing the IL-36 receptor (IL-36R) on their surface. The IL-36R is a heterodimer of IL-1 receptor-like 2 (IL-1RL2) and IL-1R accessory protein (IL-1RAP); the latter is also a cofactor for IL-1 signaling. Dysregulation of IL-36 signaling can lead to inflammatory diseases in, for example, skin, airways, and bowels. The first IL-36-targeted therapeutic to receive FDA approval (September 2022) for human use in the US was the monoclonal antibody Spesolimab directed against IL-1RL2 [1]. Similar IL-1RL2-neutralizing antibodies are being developed [2,3,4]; however, clinical trials are also ongoing in which IL-36 is used as an adjuvant in cancer immunotherapy (ClinicalTrials.gov ID NCT03739931) [5]. Thus, as the field moves forward, a detailed understanding of IL-36 system regulation and its impact on essential immune functions becomes critical for efficacious therapeutic targeting.

There are three agonist IL-36 cytokines: IL-36α, IL-36β, and IL-36γ, and two receptor antagonists: the well-defined IL-36R antagonist (IL-36Ra) and the lesser understood IL-1 family member 10 (IL-1F10), commonly referred to as IL-38 [6]. Each of these agonists and antagonists is encoded by independent genes located in the same genomic region as the IL-1 genes: *IL1A* and *IL1B* [6]. Upon IL-36 engagement of the IL-36R, intracellular signaling from the cell membrane to the nucleus ensures that gene transcription of, for example, proinflammatory cytokines is initiated (reviewed in [6]). Among these genes are neutrophil recruiting chemokines, e.g., CXCL1 and IL-8, and activators of leukocytes, such as TNF and IL-6 [6]. These are activities that in a well-functioning immune system promote protection against bacteria, fungi, and viruses (reviewed in [6]). In addition, they may contribute to cancer control [7,8,9]. However, overly strong IL-36 responses may lead to acute or chronic inflammatory diseases.

The strongest direct link to human disease is in generalized pustular psoriasis, where loss of function mutations in IL-36Ra lead to exuberant neutrophil recruitment to the skin [10]. This has led to the classification ‘deficiency of IL-36 receptor antagonist (DITRA)‘ as an autoinflammatory disorder [10]. Dysregulation of the IL-36 system, especially increased expression of one or more of the IL-36 cytokines, has also been suggested to play a role in other neutrophil rich dermatoses, plaque psoriasis, hidradenitis suppurativa, Netherton syndrome, autoimmune skin blistering, and atopic dermatitis (reviewed in [11]). Asthma, inflammatory bowel disease, and rhinitis are among additional immune mediated diseases associated with the IL-36 system [12,13,14]. As interest in blocking IL-36 signaling with IL-1RL2 neutralizing antibodies increases [1,2,3,4,15], there is a growing need to identify the IL-36 responsive cell types in vivo in healthy and diseased states.

Many previous studies have used global knockout mice to evaluate functional involvement in inflammatory or disease-related mechanisms. However, IL-1RL2 is expressed in multiple cell types, which may have complex interactions that are not detected when the receptor is absent from all cells. For example, epithelial cells in the skin, keratinocytes, exhibit a high constitutive expression of IL-1RL2, while some leukocytes that are recruited during inflammation, e.g., monocytes/macrophages, dendritic cells, and possibly T cells, also can express the receptor [6,16,17,18,19,20,21]. Little is known about possible dynamic changes during immune responses and spatial distribution within tissues. Furthermore, how IL-1RL2 expressing cells control normal immune mechanisms and cancers remains ill defined. Knowledge of such functions would provide a solid foundation for the application of IL-1RL2 neutralizing antibodies and IL-36 as immune adjuvants for, for example, cancer therapy.

Cre-mediated recombination of *loxP* loci has become a mainstay in biological and medical research as an indispensable genetic engineering tool [22]. Many diverse approaches are utilized and often inducible strategies using, for example, tamoxifen allow exquisite distinction between dynamic gene functions over time. However, many factors can impact effectiveness of the Cre-facilitated gene modification and thus careful attention to experimental design is critical for reproducible results. Here we describe the development of a new floxed *Il1rl2* mouse strain with an inserted mCherry reporter element that allows verification of successful DNA recombination. Furthermore, we demonstrate the application of this reporter element by examining the distribution of the *Il1rl2* promoter-driven gene expression in barrier tissues in direct contact with the host environment and the microbiome.

## 2. Materials and Methods

### 2.1. Generation of Floxed Il1rl2 Locus

The targeting vector and mouse strain development was commissioned at the Ingenious Targeting Laboratory (Supplemental File S1). All stages of vector development through breeding to Flp deleter mice and subsequent C57BL/6 wild type mice to remove the Flp gene were performed at the Ingenious Targeting Laboratory. The sequenced targeting vector was introduced into C57BL/6 embryonic stem cells and clones confirmed by PCR and sequencing (Supplemental File S2). Positive clones were implanted in pseudo-pregnant females. Germline transmission offspring were bred to Flp deleter mice to remove the neomycin cassette. Deletion of the Neomycin cassette was confirmed by PCR and sequencing (Supplemental File S3) and mice backcrossed to C57BL/6 mice to remove the Flp gene (Supplemental File S3). The sequence of the final targeted locus is shown in Supplemental File S4. *Il1rl2^loxP+^Flp^−^ (Il1rl2^loxP^)* founders were transferred from the Ingenious Targeting Laboratory to Temple University. Founders were intercrossed for strain maintenance or bred to Cre-expressing mice for expression analyses.

### 2.2. Generation of Cre-Expressing Strains and Genotyping

**Figure 1:**
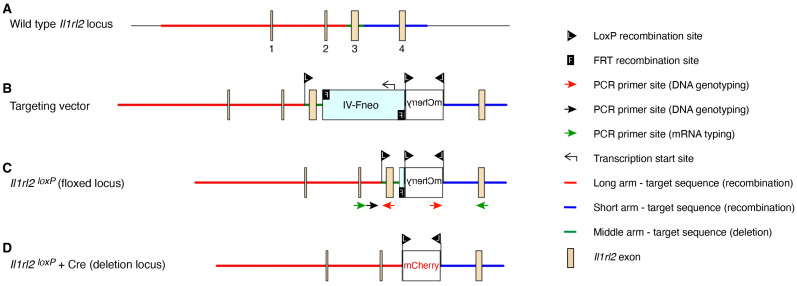
Targeting strategy for generating *Il1rl2* floxed allele with mCherry reporter gene. Schematic representation of the wild type *Il1rl2* locus (**A**), the targeting vector (**B**), the floxed *Il1rl2* locus after Flp recombination (**C**), and the *Il1rl2* deletion locus after Cre recombination (**D**). Arrows (**C**) indicate approximate positions of PCR primers for genotyping (black and red; note the two red sites are identical/one primer) and mRNA analyses (green).

*Il1rl2^loxP^* mice were mated with Cre-expressing mice. The resulting offspring were either intercrossed or backcrossed to *Il1rl2^loxP^* homozygous mice to generate *Il1rl2^loxP^* homozygous and heterozygous mice with and without the Cre gene. Mice were genotyped using ear punches and a previously described protocol [23] with the LOX1 and SDL2 primers listed in Table 1 and illustrated in Figure 1. For Cre-mediated recombination in all cells the B6.C-Tg(CMV-Cre)1Cgn/J (RRID:IMSR_JAX:006054, Jackson Laboratory) strain was used. The B6N.Cg-Tg(KRT14-cre)1Amc/J (RRID:IMSR_JAX:018964, Jackson Laboratory) was used to drive constitutive recombination in Krt14 expressing cells, including keratinocytes, in the skin. Cre negative littermates were used as controls. All procedures using mice were approved by the Temple University Institutional Animal Care and Use Committee and in compliance with the United States Public Health Service Policy on Humane Care and Use of Laboratory Animals.

### 2.3. Tissue Collection

Organs were collected from uninfected 6–12-weeks-old mice. Skin was also collected from HSV-1-infected mice. Tissues were either immediately fixed in formaldehyde or snap frozen on dry ice and stored at −80 °C until processing. For the collection of the lungs, the trachea was exposed, and a blunt needle inserted into the trachea to allow inflation of the lungs with formaldehyde. The trachea was tied off before removal of the lungs from the chest cavity. The lungs were subsequently placed in formaldehyde to complete the fixation.

### 2.4. RNA Isolation and Analyses

Mouse lungs and skins were homogenized in TRI Reagent (Zymo Research) using a Bio-Gen PRO200 homogenizer equipped with interchangeable Multi-Gen 7XL probes (PRO Scientific). RNA was isolated from homogenates using a Direct-zol RNA Miniprep Plus kit (Zymo Research) according to the manufacturer’s protocol. The total RNA at 1 µg per sample was converted to cDNA using AMV reverse transcriptase (Promega) according to the manufacturer’s instructions. The generated cDNA was examined by PCR using the primers Il1rl2E2 and Il1rl2E4 (Table 1, Figure 1) and standard agarose gel electrophoresis.

### 2.5. Herpes Simplex Virus-1 (HSV-1) Infections

Mice aged 8–10 weeks were infected with HSV-1 using a previously described protocol [24,25]. Briefly, mice were denuded with epilating cream and carefully cleaned with water. The following day the mice were infected with 1.5 × 10^6^ plaque forming units on the flank using scarification. At 5 days post-infection the skin was collected for analyses.

### 2.6. Immunohistochemistry and Immunofluorescence

Fixed tissues were embedded in paraffin. Paraffin sections were deparaffinized and hydrated through xylenes and graded alcohol, respectively. To unmask the antigens, the sections were microwaved in 10 mM sodium citrate pH 6.0. The sections were permeabilized in 1% triton x-100 in 0.05% Tween 20 (TBST) for 20 min. To block endogenous enzyme activity, the slides were incubated with 3% hydrogen peroxide. After that, the slides were blocked with 5% goat serum, 20% egg whites, and biotin solution (Vector). The mCherry protein was detected using rabbit anti-mCherry (E5D8F, Cell Signaling Technology), and incubated with corresponding horseradish peroxidase-linked secondary antibody. DAB substrates were added to visualize the mCherry expression. Slides were mounted using mounting medium. IHC stained slides were digitized using a Leica Aperio CS2 scanner and examined using Aperio ImageScope 12.4.3 for Windows 10 Professional (https://www.leicabiosystems.com/digital-pathology/manage/aperio-imagescope/, accessed on 3 May 2024). Additional images were acquired using an OLYMPUS microscope (IX71).

Immunofluorescence microscopy for CD45 and mCherry was performed on deparaffinized histologic slides after heat-mediated antigen-retrieval in citrate solution. Slides were blocked for 1 h at room temperature in PBS with 2% FBS (blocking buffer), and then incubated at 4 °C overnight with rat anti-CD45 (30-F11, Cell Signaling Technology, Danvers, MA, USA, 1:200) and rabbit anti-mCherry (E5D8F, 1:200) primary antibody diluted in blocking buffer. The next day, slides were incubated for 1 h at room temperature with goat anti-rat IgG-FITC (Santa Cruz Biotechnology, Dallas, TX, USA, sc-2011, 1:200) and goat anti-rabbit IgG-TR (Santa Cruz Biotechnology, sc-2780, 1:200) diluted in blocking buffer, subsequently stained with DAPI (Invitrogen, Carlsbad, CA, USA, D3571) and mounted with Sub-x Mounting Medium (Leica, Wetzlar, Germany). Staining was visualized using an OLYMPUS microscope (Tokyo, Japan, IX71, 400× magnification).

## 3. Results

### 3.1. Il1rl2 Targeting Strategy

In our previous work we identified a significant involvement of IL-36 cytokines in skin inflammation [26] and protective immunity against the herpes simplex virus (HSV) [24,25]. While some of these activities appeared identical to those of IL-1 [27,28], we also noted differences in our model systems. The IL-1RL2-engaged cells with physiological relevant functions can be identified through use of Cre–lox-mediated conditional deletion in individual cell types. However, functions may change over time; thus, mandating the use of inducible models such as the tamoxifen–Cre system. These stimulated approaches require confirmation of recombination. We therefore set out to develop a floxed *Il1rl2* mouse strain incorporating a reporter cassette that is activated upon Cre-mediated recombination (Figure 1). To avoid potential conflict between the reporter sequence and the splicing of the *Il1rl2* loci, we required a reporter smaller than 1000 bp. Since the skin can have significant green autofluorescence, we therefore chose mCherry as the reporter.

The *Il1rl2^loxP^* locus was generated through homologous recombination. The targeting allele contained parts of the wild type (C57BL/6) *Il1rl2* gene spanning 3476 bp of the promoter upstream of the 5′ untranslated region in XM_036165947, exons 1–4, and 712 bp downstream of exon 4 (Figure 1A,B, Supplemental File S4). A neomycin selection cassette was inserted between exons 3 and 4 together with a reversed mCherry coding sequence. Three *loxP* sites flanking exon 3 and the ends of the mCherry sequence were included; thus, in the final successfully targeted allele (Figure 1C and Supplemental File S4), Cre recombination will invert the mCherry cassette, allowing the expression of mCherry protein, while the *Il1rl2* exon 3 will be deleted (Figure 1D). The elimination of exon 3 results in a reading frame shift that prevents the expression of the IL-1RL2 protein.

After germline transmission of the initially targeted *Il1rl2* gene, the neo cassette was removed by breeding the mice to *Flpo* deleter mice (Supplemental File S3). Germline transmission of targeted *Il1rl2* with neo removed (*Il1rl2^loxP^*, Figure 1C) was confirmed and mice bred to wild type C57BL/6 mice to remove the *Flpo* gene (Supplemental File S3).

### 3.2. Functional Validation of Floxed Locus

**Figure 2:**
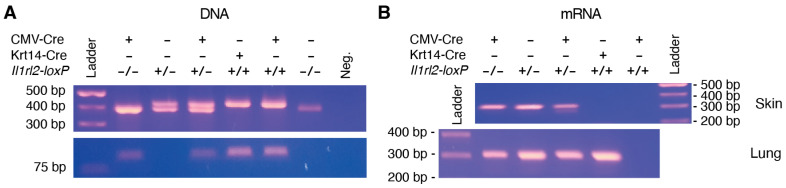
Validation of DNA locus and mRNA expression. (**A**) Upper panel: Mice were genotyped for the *Il1rl2^loxP^* locus using the black and red primers in Figure 1C to identify wild type (396 bp) and the *Il1rl2^loxP^* locus (428 bp or 430 bp before and after recombination, respectively). Note that the two red sites are identical/one primer. Lower panel: Primers for Cre transgene generate an approximately 100 bp product. (**B**) RNA was isolated from the skin and lungs of representative mice from (**A**), reverse transcribed, and PCR performed with primers for *Il1rl2* exons 2 and 4 (green arrows Figure 1, Table 1). The *Il1rl2* mRNA with exon 3 present is expected to produce a 304 bp product. Deletion of exon 3 will result in the generation of early stop codons in exon 4 when exon 2 is spliced to exon 4.

To confirm functionality of the individual allele elements in the targeted locus, *Flp^−^ Il1rl2^loxP^* mice were further crossed with two Cre strains. Keratinocytes have extensively been associated with IL-1RL2 activity and thus a Krt14-Cre strain was used to delete IL-1RL2 in this cell type. In addition, we used a CMV-Cre strain to disrupt *Il1rl2* in all cells. Heterozygous Cre mice were mated with mice heterozygous or homozygous for the floxed *Il1rl2* locus. DNA genotyping of offspring revealed the expected banding patterns (Figure 2A) suggesting correct DNA recombination had taken place in the presence of Cre (Figure 1D).

To further examine the integrity of the floxed *Il1rl2* locus, we also evaluated the ability of the modified gene to generate a working *Il1rl2* mRNA in the absence of Cre, a prerequisite for the use of the mouse strain for generating conditional knockout mice. RNA was isolated from skin and lungs and examined by RT-PCR. Tissues were collected from *Il1rl2^loxP^* heterozygous and homozygous mice, which additionally were either Cre-negative or expressed *Cre* under either the Krt14 or CMV promoters (Figure 2B). The predicted PCR band was observed, as expected, in all tissues from mice with the wild type *Il1rl2* locus heterozygous as well as homozygous. In contrast, the band was absent in both skin and lungs from *Il1rl2^loxP^* locus homozygous mice expressing *Cre* under the control of the CMV promoter (Figure 2B). Furthermore, the band was present in lungs, but not skin, from *l1rl2^loxP+/+^* mice expressing *Cre* under the Krt14 promoter (Figure 2B). This demonstrates the principle of conditionally knocking out expression in specific cell types and tissues. Furthermore, it indicates that *Il1rl2* mRNA is expressed and spliced correctly from the *Il1rl2* locus and that Cre-mediated recombination deletes exon 3 as intended.

### 3.3. Functional Validation of mCherry Reporter in Skin

**Figure 3:**
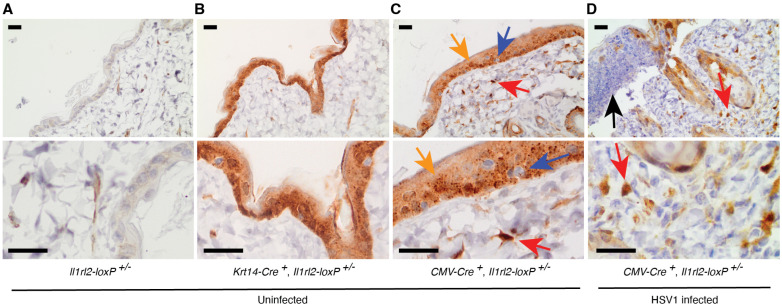
The *Il1rl2* promoter is active in interfollicular and follicular epidermal keratinocytes and immune cells in the dermis, but not fibroblasts or neutrophils. Mice with indicated genotypes were left uninfected (**A**–**C**) or infected with HSV-1 (**D**). (**D**) Skin was collected on day 5, post-infection. (**A**–**D**) Skin was fixed in formaldehyde and mCherry protein detected by IHC. Yellow and blue arrows identify mCherry positive and negative, respectively, cells in the epidermis. Red arrows point to mCherry positive cells in the dermis. Black arrow points to mCherry negative neutrophils in wound scab. Scale bars represent 20 μm.

The two above used deleter mouse strains utilize constitutively active promoters to drive the expression of Cre. Nevertheless, some functional studies may require that protein activity is present at the onset of an experiment and only during a preset time course is the function eliminated. This can be achieved through use of drug-activated, for example, tamoxifen, Cre mutants. However, because the drug is delivered into the mice, it may not work equally efficiently every time or in all tissues, thus requiring confirmation of the drug-induced gene inactivation. In the present locus design (Figure 1), the inclusion of the inverted *mCherry* sequence (Figure 1B,C) allows the distinction between cells already known to express *Il1rl2* with and without Cre-mediated deletion of exon 3. As the *loxP* recombination event will reverse the *mCherry* sequence, cells with the desired exon 3 deletion will express *mCherry* mRNA under the control of the *Il1rl2* promoter. Cells without the recombination event will express the wild type *Il1rl2* mRNA and not mCherry. To illustrate this feature and verify its functionality in our new mouse strain, we examined mCherry expression in skin (Figure 3). To avoid confounding from potential IL-1RL2 dependent outcomes, e.g., reduced recruitment of leukocytes into the skin, the skin was collected from *l1rl2^loxP^* heterozygous mice to allow a functional *Il1rl2* allele to remain in all cells. As predicted, in the absence of Cre, no specific staining for mCherry was detected (Figure 3A).

The skin is a tissue well known to have a high degree of IL-36R expression in keratinocytes in the epidermis and possibly some leukocytes [6]. Here, the mCherry expression in skin from *Krt14-Cre^+^*, *Il1rl2-loxP^+/−^* mice was detected in epidermal keratinocytes and hair follicles, which are extensions of the epidermis (Figure 3B). In the *CMV-Cre^+^*, *Il1rl2-loxP^+/−^* mice, mCherry could also be detected in dermal leukocytes (Figure 3C, red arrow), while some cells in the epidermis were negative for mCherry (blue arrows, Figure 3C); these latter cells may be Langerhans or γδ T cells. Because of our previous functional studies of IL-36 during HSV-1 infections [24,25], we also examined HSV-1-infected skin (Figure 3D). In this tissue, intact epidermal keratinocytes and a larger number of dermal leukocytes expressed mCherry, while the neutrophils recruited to lesions (Figure 3D, black arrow) appeared negative. This further demonstrates the ability to drive recombination in specific cell types.

### 3.4. Application of mCherry Reporter to Identify IL-1RL2 Expressing Cells in Barrier Tissues

Early preliminary studies of the IL-36 cytokines and their receptor suggested a predominant expression of the system in epithelial tissues [6]; however, due to limited availability of suitable reagents, a full characterization was never undertaken. Thus, we decided to utilize the mCherry reporter element in the *CMV-Cre^+^*, *Il1rl2-loxP^+/−^* mice to examine the IL-1RL2 expression pattern in additional tissues. Due to our own interests in host interactions with microbes, we focused on the barrier tissues with direct contact with the external environment.

#### 3.4.1. Small Intestine and Colon

**Figure 4:**
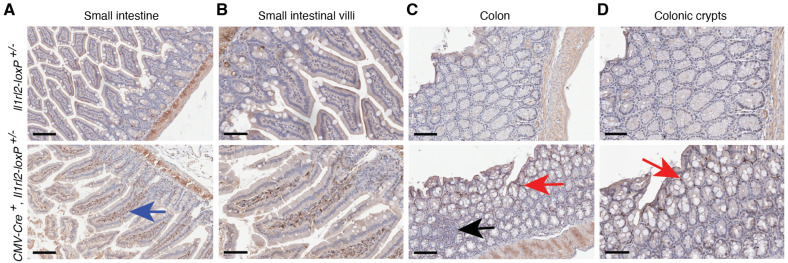
The *Il1rl2* promoter is active in normal leukocytes in the small and large intestines. The intestines were collected from mice with the indicated genotypes and examined for the mCherry *Il1rl2* reporter using IHC. The blue and red arrows identify mCherry-positive cells in the small intestinal villi and colonic crypts, respectively. The black arrow points to the mCherry-negative MALT. Scale bars represent 100 (**A**), 60 (**B**), 100 (**C**), and 70 (**D**) μm, respectively.

Functionally, the IL-36 system was initially investigated in the skin in the context of psoriasis [27,29,30], but studies of inflammatory bowel disease quickly followed [31,32,33,34]. Here we examined the *Il1rl2* promoter-driven mCherry reporter expression in the small and large intestines from normal, healthy *CMV-Cre^+^*, *Il1rl2-loxP^+/−^* mice. We did not detect the *Il1rl2* reporter in epithelial cells in either tissue (Figure 4). The mucosal associated lymphoid tissue (MALT) was also negative for mCherry (Figure 4C, black arrow). However, mCherry-positive cells were present below the epithelium in both the villi and crypts (Figure 4). While these cells could be leukocytes [6], the presence of the IL-36R on fibroblasts has been implied by functional in vitro studies, e.g., [32,35].

**Figure 5:**
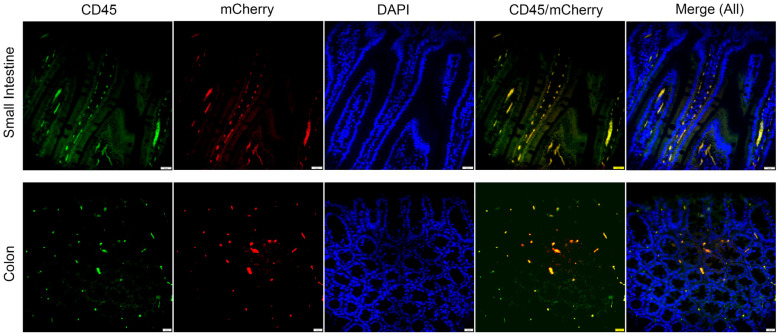
Leukocytes in the small and large intestines express the *Il1rl2* reporter. Tissues from *CMV-Cre^+^*, *Il1rl2-loxP^+/−^* mice in Figure 4 were stained for CD45 (green), a leukocyte marker, and mCherry (red). Nuclei were stained with DAPI. Scale bars represent 20 μm.

Fibroblasts exhibit great heterogeneity in the intestines [36]; thus, to assess the cellular subtype of the mCherry-positive cells, we performed double stained immunofluorescence analyses for the leukocytes and the *Il1rl2* reporter (Figure 5). All mCherry-positive cells were also stained for CD45, which identified them as leukocytes. Thus, in normal small intestines and colons in mice, the *Il1rl2* promoter appears to be only active in leukocytes.

#### 3.4.2. Lungs and Trachea

**Figure 6:**
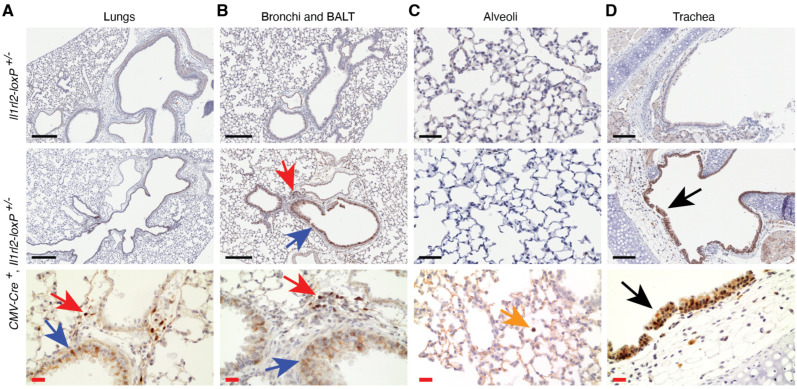
The *Il1rl2* promoter is active in epithelial cells and leukocytes in the airways. Lungs of mice with the indicated genotypes were inflated, collected, and fixed in formaldehyde. Sections were stained with antibodies directed against mCherry. Blue arrows point to mCherry-positive cells in the conductive airway epithelium. Red arrows identify mCherry-positive cells in the BALT. Yellow arrow points to mCherry-positive leukocyte in the alveoli. Black arrows point to mCherry-positive epithelium in the trachea. Black scale bars represent 300 (**A**), 200 (**B**), 50 (**C**), and 100 (**D**) μm, respectively. Red scale bars represent 20 μm.

As in the skin [6], IL-36 signaling in the lungs promotes inflammation [37,38,39,40,41,42,43]. While this can be important for protective immunity against viruses and bacteria that target the lungs, the system can also have adverse effects leading to pulmonary disease. Many unknowns remain about how these beneficial and detrimental effects are initiated and regulated. A thorough elucidation of these will require detailed understanding of when and how different cell types within the lungs are activated. We therefore applied the above-described approach to identity IL-36R expressing cells in the airways and lungs (Figure 6). Within the lungs we observed a strong expression of the *Il1rl2* reporter in the bronchial epithelium and in leukocytes in the bronchus-associated lymphoid tissue (BALT) (Figure 6B). Rare leukocytes, likely macrophages, were positive for mCherry in the alveoli, but the alveolar epithelium did not reveal any significant mCherry expression (Figure 6C). In contrast, the epithelium in the trachea revealed very strong staining for the reporter (Figure 6D). Thus, the IL-36 receptor might function in normal tissue primarily in the airway epithelium and leukocytes within the lungs.

#### 3.4.3. Oral Mucosa and Tongue

**Figure 7:**
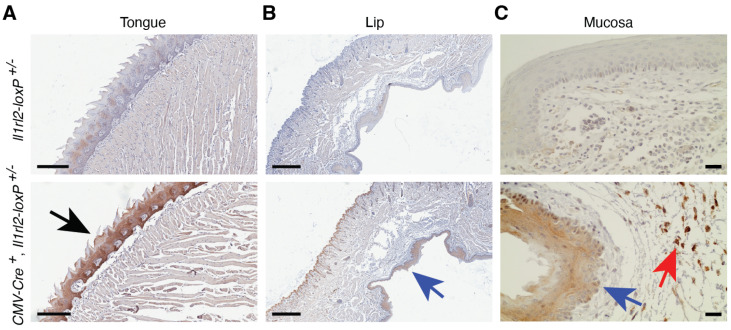
The epithelium of the tongue and buccal mucosa exhibit *Il1rl2* promoter activity. The tongue and lower lips of mice with the indicated genotypes were examined for *Il1rl2* mCherry reporter activity using IHC. Black arrow indicates reporter positive epithelium on the top of the tongue. Blue arrows point to positive mucosal epithelium in the lip. Red arrow shows positive leukocytes. Scale bars represent 200 (**A**), 500 (**B**), and 20 (**C**) μm, respectively.

The IL-36 system has been shown to activate expression of proinflammatory cytokines in oral epithelial cells [44,45]. This activity may be beneficial for the control of bacteria, e.g., *Porphyromonas gingivalis* and *Prevotella melaninogenica* [46,47], and *Candida albicans* fungi [48], but may also promote gingivitis, periodontitis, and lichen planus [47,49,50,51,52]. We found that the mCherry *Il1rl2* reporter was highly expressed in epithelial cells on the dorsal surface of the tongue (Figure 7A, black arrow). In the mucosa of the lip, reporter expression could be detected in epithelial cells (Figure 7B,C, blue arrows) and leukocytes located in the submucosa, below the mucosal membrane (Figure 7, red arrows). This provides further support for IL-36 signaling having important functions in the oral cavity.

#### 3.4.4. Vagina and Uterus

**Figure 8:**
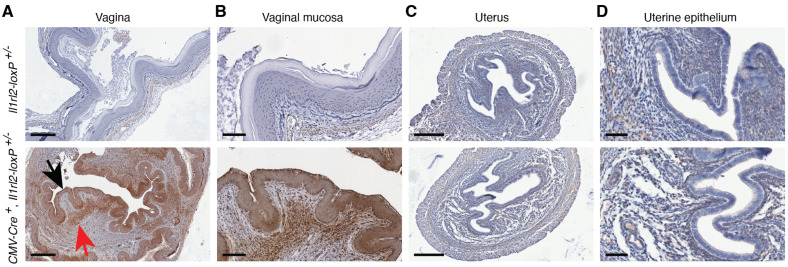
IL-1RL2 expression is restricted to the vagina in the normal female reproductive tract. Using IHC, mCherry expression, under the control of the *Il1rl2* promoter, was examined in vaginas and uteri from mice with the indicated genotypes. Black arrow indicates reporter positive epithelium in the vagina. Example of positive leukocytes in the vaginal submucosa is indicated with a red arrow. Scale bars represent 300 (**A**), 100 (**B**), 200 (**C**), and 50 (**D**) μm, respectively.

The vagina is another barrier tissue in which the IL-36 system has been studied. So far it has been associated with host defense against bacteria and viruses [53,54,55,56,57], hormonal changes [58], and cancer [59]. Our analyses of mCherry reporter expression demonstrate potent *Il1rl2* promoter activity in the vaginal epithelium and leukocytes in the lamina propria below (Figure 8A,B). We also examined the uterus; however, no reporter activity was detected in this tissue (Figure 8C,D). Thus, it would appear that, at least in normal tissue, the IL-36R function is restricted to the vagina in the female reproductive tract.

#### 3.4.5. Esophagus and Stomach

**Figure 9:**
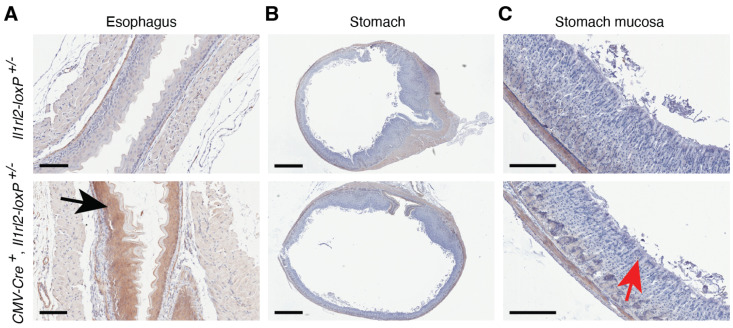
The epithelium in the esophagus, but not the stomach, expresses IL-1RL2. The esophagi and stomachs of *Il1rl2* reporter mice with the indicated genotypes were examined by IHC for mCherry expression. The black arrow points to the mCherry-positive epithelium in the esophagus. The red arrow indicates the mCherry-negative epithelium in the stomach. Scale bars represent 100 (**A**), 1000 (**B**), and 200 (**C**) μm, respectively.

While the IL-36 system has been studied in the oral cavity and the intestine, the barrier tissues between those sites, i.e., the esophagus and the stomach, have not been examined. Here, we identified potent expression of the *Il1rl2* mCherry reporter in the epithelium of the esophagus (Figure 9A, black arrow). In contrast, no expression was detected in the stomach (Figure 9B,C). This suggests that in a normal esophagus, but not the stomach, the IL-36 system has one or more functions important for maintaining homeostasis.

#### 3.4.6. Urinary Tract

**Figure 10:**
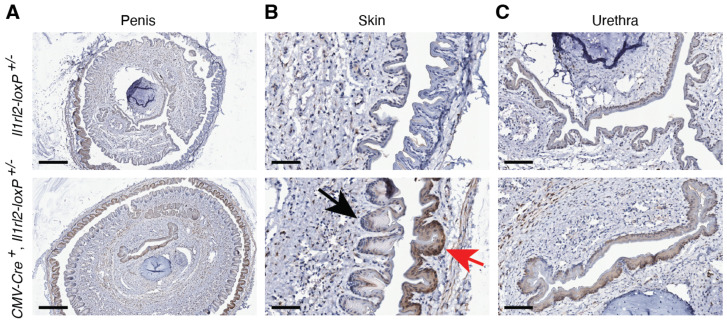
The epithelial cells in the foreskin, the penis skin, and the urethra have *Il1rl2* promoter activity. IHC was used to identify mCherry *Il1rl2* reporter activity in penises collected from mice with the indicated genotypes. Scale bars represent 300 (**A**), 60 (**B**), and 90 (**C**) μm, respectively.

Another important epithelium that has not been studied is that of the urethra. Here, for the ease of tissue collection, we focused on the male urinary tract. IHC analyses of penises from reporter mice (Figure 10A) identified mCherry expression in the skin, with noticeably stronger expression in the foreskin compared to that of the skin on the penis itself (Figure 10B, red and black arrows, respectively). In addition, we observed *Il1rl2* reporter activity in the urethra (Figure 10C). The staining in the urethra was weaker than in the foreskin (Figure 10A). Thus, the IL-36 system appears to be present in both the urinary tract and the skin associated with the male penis.

## 4. Discussion

The IL-36 cytokines were discovered more than 20 years ago [6]. Soon after, they were determined to signal through the orphaned receptor IL-1Rrp2, now known as IL-1RL2 [29]. IL-36Ra and IL-1F10/IL-38 are additional ligands for IL-1RL2 and were discovered around the same time [6]. However, the physiological and pathological functions of this extended system remain poorly understood. To enable in-depth mechanistic studies, we generated and carefully validated a new mouse strain that will allow targeted inactivation of IL-1RL2 in specific cell types (Figure 1). Furthermore, the targeting strategy included a reporter gene that allows verification of Cre-mediated recombination (Figure 1). To demonstrate the application of this reporter, we here utilized it to examine the distribution of IL-1RL2 expression in several barrier tissues (Figure 3, Figure 4, Figure 5, Figure 6, Figure 7, Figure 8, Figure 9 and Figure 10). When Cre was expressed under the control of a CMV promoter in all cells, reporter-positive leukocytes were found in most of the tissues examined. In contrast, expression in epithelial cells exhibited a more tissue-dependent distribution (Figure 3, Figure 4, Figure 5, Figure 6, Figure 7, Figure 8, Figure 9 and Figure 10) with strong expression in the epithelial cells of the skin, upper airways, tongue, esophagus, and vagina. Modest expression was detected in the oral mucosa and the urinary tract. Limited or no expression was detected in the epithelial cells in the small and large intestine, lung alveoli, uterus, and stomach (Figure 3, Figure 4, Figure 5, Figure 6, Figure 7, Figure 8, Figure 9 and Figure 10). Our newly developed mouse strain, described in the present study, will allow further research into the specific functions of IL-1RL2 in these many distinct tissues and cell types through application of Cre-directed recombination to generate conditional knockout sub-strains.

In early studies associated with the discovery of the IL-36 system, the strongest expression of IL-36 cytokines and IL-1RL2 was observed in tissues with epithelial cells [60,61]. In the skin, it was specifically demonstrated that keratinocytes, but not fibroblasts, endothelial cells or melanocytes, express the *Il1rl2* mRNA [61]. These experiments utilized Northern blotting, RNase protection assays and in situ hybridization as the detection methods; approaches that offer availability and specificity in the absence of quality antibodies, but which also can be technically challenging. The expression pattern we observe here in the epidermis by using the mCherry reporter for IL-1RL2 promoter activity and standard immunohistochemistry (Figure 3) correlates with those early findings. Furthermore, we were also able to detect low numbers of mCherry-positive leukocytes in the dermis, and that the number of these increased during a viral skin infection (Figure 3). Immunohistochemistry is a well-established technology that is relatively straight forward to set up in many laboratories. As such, the here illustrated approach for identifying IL-1RL2-expressing cells in tissues and their specific locations within those tissues is one that can easily be adapted in most research settings.

In addition to skin, we also examined IL-1RL2 reporter activity in several barrier tissues in which the function of the IL-36 system previously was investigated directly in mice or indirectly with cell cultures. In support of studies involving oral and vaginal epithelial cells, e.g., [44,45,53,54], we observed strong IL-1RL2 reporter activity in the epithelial cells in the tongue and the oral and vaginal mucosa (Figure 7 and Figure 8, black and blue arrows). These tissues also contained mCherry positive leukocytes (Figure 7 and Figure 8, red arrows) suggesting multiple functions for IL-1RL2 ligands in these tissues. Such functions may be defined through the application of cell type specific conditional knockout mice derived from the here generated mouse strain.

Several in vivo studies of lung infections and the IL-36 system have been conducted in mice [37,38,39,40,41,42,43]. The lungs are made up of many different types of epithelial cells. Type I and type II alveolar epithelial cells form the gas exchanging alveoli, while undifferentiated basal and differentiated ciliated epithelial cells form the conducting airways with epithelial goblet and club cells interspersed. Interestingly, we observed a very distinct differential expression of the IL-1RL2 reporter in these different types of epithelial cells with the strongest expression in the upper bronchial airways with a gradual reduction in expression as the airways branch deeper into the lungs with most of the alveolar cells being negative for mCherry (Figure 6A–C). Epithelial cells in the trachea also stained strongly for the IL-1RL2 reporter (Figure 6D). As in other tissues examined, IL-1RL2 reporter-positive leukocytes were also present in uninfected lungs (Figure 6). The differential involvement of these distinct cell types in normal lung functions, immunity, infections, and chronic diseases have not been delineated. Thus, it appears that several opportunities to refine our understanding of the IL-36 system in lung function and infections exist.

The IL-36 system has been linked to inflammation, fibrosis, and tissue repair in the intestine [31,32,62,63,64,65]. Some of these in vivo studies in mice focused on dendritic and T cell controlled inflammatory responses [63,64,65]. In the unchallenged mice examined here, the MALT, a site rich in dendritic cells, was negative for mCherry expression (Figure 4C). Instead, we observed the presence of leukocytes with IL-1RL2 promoter activity in the lamina propria in both the small intestine and the colon (Figure 4 and Figure 5). These leukocyte populations may be responsible for initiating inflammation in the gut in the above studied models. However, unlike, for example, the skin and vaginal mucosa, where we observe extensive reporter staining in the epithelial cells, the epithelial cells in the small intestine and colon were negative for mCherry in our analyses (Figure 4 and Figure 5). This differs from the reported expression of IL-1RL2 in epithelial cells in the human colon [66] and IL-1RL2 ligand engagement in human cell lines with epithelial morphology and derived from colorectal adenocarcinomas [67]. Whether these discordant outcomes reflect differences between humans and mice, tumor-dependent expression, or, perhaps, issues with the antibodies directed against human IL-1RL2, for which true controls are lacking, remains to be determined. Our observations here that colon epithelial cells and fibroblasts are negative for IL-1RL2 promoter activity (Figure 4 and Figure 5) is also in disagreement with studies describing IL-36-induced responses in colonic epithelial cells and fibroblasts, e.g., [32,34,35,62]. Such differences may be due to the use of cell lines derived from cancer cells, the altered expression profiles of cells maintained in cultures, and/or secondary responses driven by traces of other cell types in primary cultures. The application of in vivo models utilizing conditional KO approaches may resolve these discordant observations. Possible mechanisms that could be explored are the roles of the microbiome and γδ T cells in shaping *Il1rl2* mRNA expression in the small and large intestines.

The dramatic differences in *Il1rl2* promoter activity observed in the barrier tissues examined here (Figure 3, Figure 4, Figure 5, Figure 6, Figure 7, Figure 8, Figure 9 and Figure 10) may be surprising given previous studies, as outlined above. Barrier tissues are often described as those in contact with microbes and there is increasing interest in studies of both how the microbiome may shape expression of the IL-36 system, and vice versa, how the latter may influence the former (reviewed in [68]). Interestingly, our data demonstrate a differential and distinct expression pattern of the IL-36 receptor across various barrier tissues (Figure 3, Figure 4, Figure 5, Figure 6, Figure 7, Figure 8, Figure 9 and Figure 10). Both the skin and the intestine, especially the colon, are extensively studied in the context of the microbiome. Yet, while both tissues have *Il1rl2* reporter-positive leukocytes, only the skin has expression in the epithelial cells (Figure 3, Figure 4 and Figure 5). One aspect that is different in these two tissues and their associated microbiomes is the dynamics of the microbiomes and frequency of exposures to potential pathogens. The skin microbiome undergoes significant changes in early life [69]; however, even in adulthood there are significant variations in the bacterial, fungal, and viral microbiomes from one topographical site to another [70,71,72]. Due to their external locations, these sites are inherently subject to more daily possibilities for fluctuations. In contrast, due to the low pH of the stomach environment, which does not have detectable *Il1rl2* reporter activity (Figure 9B,C), the microbiome in the intestine may be more stable. The epithelial tissues here identified as having significant *Il1rl2* promoter activity, are those in contact with the external environment, i.e., the skin, the oral cavity, the upper airways, the esophagus, the vagina and the urinary tract, while those in more protected, even sterile, locations, e.g., the small intestine, the colon, the stomach and the uterus, do not express IL-1RL2 in epithelial cells. This may suggest that an essential function of the IL-36 system in *Il1rl2*-positive epithelia is to act as a first line of defense against foreign microorganisms. This aligns with our previous hypothesis that the IL-36 system acts as a countermeasure to overcome microbial immune evasion strategies [6].

## 5. Conclusions

In summary, our data presented here reveal the presence of the IL-36 receptor in multiple barrier tissues, including some which have not previously been studied. While great progress in our understanding of the IL-36 system has been made during the last decade, many unknowns about normal physiological functions in diverse organ systems remain to be uncovered. Furthermore, our insight into how the system causes and protects against disease is only beginning to materialize. The here described mouse strain will represent a valuable tool in the continued exploration of beneficial and detrimental activities. Investigators interested in using the strain should contact the corresponding author.

## Figures and Tables

**Table 1:** Primers used for genotyping DNA and mRNA. Primers are shown in 5′–3′ orientation. PNDEL1 and PNDEL2 were used to screen for presence of the Neomycin cassette (428 bp product). Primers in bold are shown in Figure 1 with matching colors.

**Forward Primers**	**Reverse Primers**
**LOX1: AGGGAAGCTGTCTTTAGAACCAAGC**	**SDL2: CACACGTATCTGGGGAAGGAAAGG**
PNDEL1: TCCCAAGTCTCCCTCTCCAT	PNDEL2: CCATCTGTTGTTTGCCCCTC
CHRRYSC1: CACCCTTGGTCACCTTCAGCTTGG	A2: ACCTTCAAGGACCTGTGTCATTCC
**Il1rl2E2: TTGCTCTTCTGTGGGGTGTTT**	**Il1rl2E4: GGTAGCAGTTGTGGGCATTC**
Cre: GCGGTCTGGCAGTAAAAACTATC	Cre: GTGAAACAGCATTGCTGTCACTT

## Data Availability

The original contributions presented in the study are included in the article/Supplementary Materials, further inquiries can be directed to the corresponding author.

## Supplementary Materials

The following supporting information can be downloaded at: https://www.mdpi.com/article/10.3390/cells13090787/s1, Supplemental File S1, Supplemental File S2, Supplemental File S3, Supplemental File S4.
