# Supplementary material for: Floxed Il1rl2 Locus with mCherry Reporter Element Reveals Distinct Expression Patterns of the IL-36 Receptor in Barrier Tissues
Source: Cells. 2024 May 6;13(9):787. doi: 10.3390/cells13090787 (PMC11083296; doi:10.3390/cells13090787)
Supplement: Supplementary file 1 [file cells-13-00787-s001.zip › Supplemental file S1.pdf]

ingenious targeting laboratory

JENSEN: Construction of Il1rl2 cKO And mCherry KI Targeting Vector

Sections:

1. Vector Design Outline
2. Sequence Data Analysis
3. Cassette Sequences

1. Vector Design Outline

A 9.4 kb region used to construct the targeting vector was first sub cloned from a positively identified C57BL/6 BAC clone (RP23-216C5) using a homologous recombination-based technique. The region is designed in such a way that the long homology arm (LA) extends ~6.2 kb 5' to the distal LoxP cassette and the short homology arm (SA) extends ~2.1 kb 3' to the inversion casstte. A FRT-flanked Neo cassette was inserted downstream of exon 3 and placed immediately upstream of the inversion cassette.

The targeting vector was confirmed by restriction analysis and sequencing after each modification step. The boundaries of the 2 homology arms were confirmed by sequencing with P6 and T73 primers that read through both sides of the backbone vector into the genomic sequences. IV-Neo N3 and iNeo N2 primers read from the selection cassette into the inversion cassette (IV-Neo N3) and the 3' end of the MA (iNeo N2). Primer JENS LOX1 was used to sequencing confirm the distal LoxP insertion. Primers mCherry SQ1-2 and JENS SQ2 were used to confirm the remaining sequence of the inversion cassette.

Targeting Vector For Il1rl2 cKO and mCherry Expression

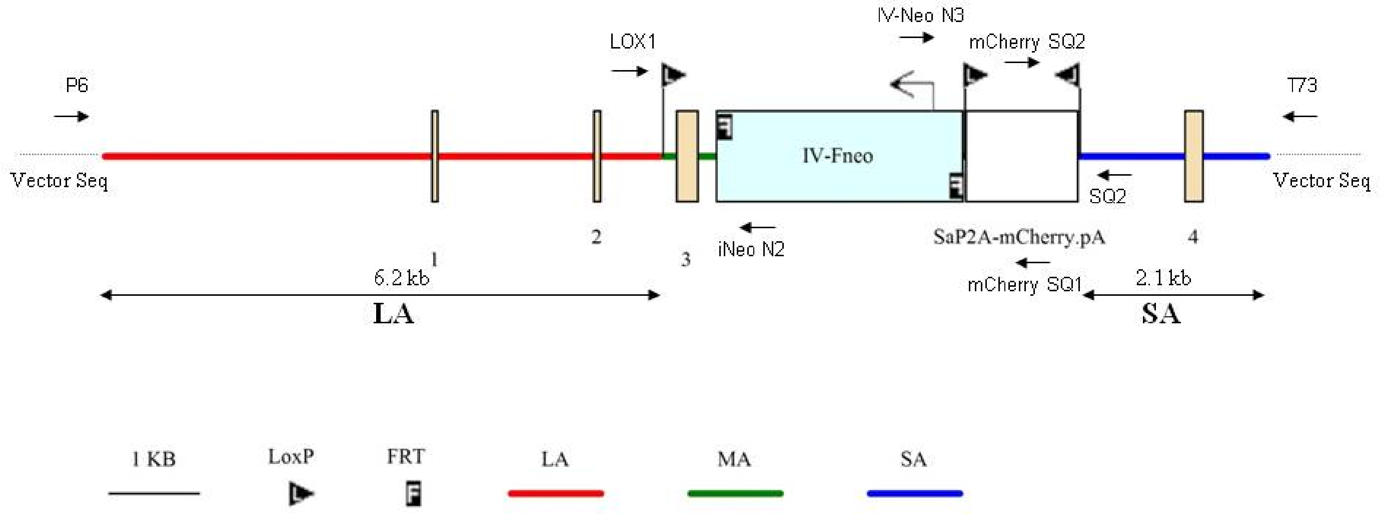

Primers used for sequencing:

```
Primer P6:          5'- GAG TGC ACC ATA TGG ACA TAT TGT C       -3'
Primer T73:         5'- TAA TGC AGG TTA ACC TGG CTT ATC G       -3'
Primer iNeo N2:     5'- AGT ATG GCT TTC CTT CCC GAT GG          -3'
Primer IVNeo N3:    5'- TCT AAG GCC GAG TCT TAT GAG CAG         -3'
Primer JENS LOX1:   5'- AGG GAA GCT GTC TTT AGA ACC AAG C       -3'
Primer JENS SQ2:    5'- CAT TAC CTC TGG CAC ATG GAT TC          -3'
Primer mCherry SQ1: 5'- AGA CCG CCA AGC TGA AGG TGA C           -3'
```

2200 Smithtown Avenue, Ronkonkoma NY 11779 ● tel: (631) 468-8530 Ext.118 ● email: alu@genetargeting.com

Primer mCherry SQ2: 5’- AGT TCA TCA CGC GCT CCC ACT TG -3’

2200 Smithtown Avenue, Ronkonkoma NY 11779 ● tel: (631) 468-8530 Ext.118 ● email: alu@genetargeting.com

Backbone Vector Information

iTL cloning vector (~2.45 kb) derived from pSP72 (Promega) contains an ampicillin selection cassette for retransformation of the construct prior to electroporation. A FRT flanked Neomycin cassette was inserted into the gene as described in the project schematic. The targeting construct can be linearized with NotI prior to the electroporation of ES cells.

The total size of the targeting construct (including vector backbone and a DT cassette) is ~ 16.9kb.

2200 Smithtown Avenue, Ronkonkoma NY 11779 ● tel: (631) 468-8530 Ext.118 ● email: alu@genetargeting.com

ingenious targeting laboratory

2. Sequence Data Analysis

Sequencing Data from Targeting Construct

P6 primer sequencing data aligned with targeted allele sequence

```
Query  10449  AGC-ACAAACCTTTGTCCATGCTGCTCTACTTGAAGAATGCTCTGTAAGTTGCTATTAAG  10507
              ||| ||||||||||||||||||||||||||||||||||||||||||||||||||||||||
Sbjct  130    AGCTACAAACCTTTGTCCATGCTGCTCTACTTGAAGAATGCTCTGTAAGTTGCTATTAAG  189

Query  10508  GCTGCCAAATGGATAGCTTTTCTGTTTCTCTTGGCCCTCTGGATTTTCTTTCCCCTAGTT  10567
              ||||||||||||||||||||||||||||||||||||||||||||||||||||||||||||
Sbjct  190    GCTGCCAAATGGATAGCTTTTCTGTTTCTCTTGGCCCTCTGGATTTTCTTTCCCCTAGTT  249

Query  10568  TTTAGATAGGCAGAACCAAGCCACCCTTTCCAAAGTCACTTGCTCCTCTCCCTGATAGTT  10627
              ||||||||||||||||||||||||||||||||||||||||||||||||||||||||||||
Sbjct  250    TTTAGATAGGCAGAACCAAGCCACCCTTTCCAAAGTCACTTGCTCCTCTCCCTGATAGTT  309

Query  10628  TGCTTGGTGTGGTTACAGAAGGACAGTAGCTCTTCCGGTTCTTGGCTGACTACATTCCAC  10687
              ||||||||||||||||||||||||||||||||||||||||||||||||||||||||||||
Sbjct  310    TGCTTGGTGTGGTTACAGAAGGACAGTAGCTCTTCCGGTTCTTGGCTGACTACATTCCAC  369

Query  10688  AGAGACTGTGCGGCTCTCTCACACACAGGGTTGCAGTGTTGGGCACACACCTAGAAATCA  10747
              ||||||||||||||||||||||||||||||||||||||||||||||||||||||||||||
Sbjct  370    AGAGACTGTGCGGCTCTCTCACACACAGGGTTGCAGTGTTGGGCACACACCTAGAAATCA  429

Query  10748  CTGTGAGTGTGAGAGTCAAAACCGATGTCCTTTGAAATGAATGCTTCTTTATTAGTTAAG  10807
              ||||||||||||||||||||||||||||||||||||||||||||||||||||||||||||
Sbjct  430    CTGTGAGTGTGAGAGTCAAAACCGATGTCCTTTGAAATGAATGCTTCTTTATTAGTTAAG  489

Query  10808  TATTAATGTTTAAAAGCTTTACTTACAAGTACCTGGGCTATGGGACTGCCTTTGCTCCCG  10867
              ||||||||||||||||||||||||||||||||||||||||||||||||||||||||||||
Sbjct  490    TATTAATGTTTAAAAGCTTTACTTACAAGTACCTGGGCTATGGGACTGCCTTTGCTCCCG  549

Query  10868  ATTCATGCCAACCTTCATTAATGGCCAAGTGTGCTGAGACAGGAGCCAGGTCCTCTAACT  10927
              ||||||||||||||||||||||||||||||||||||||||||||||||||||||||||||
Sbjct  550    ATTCATGCCAACCTTCATTAATGGCCAAGTGTGCTGAGACAGGAGCCAGGTCCTCTAACT  609

Query  10928  CCCAGATAACTAGTTACCCTTTCTAACCATCTTCTTTCTGCAGATGACATTGGAGAGGAA  10987
              ||||||||||||||||||||||||||||||||||||||||||||||||||||||||||||
Sbjct  610    CCCAGATAACTAGTTACCCTTTCTAACCATCTTCTTTCTGCAGATGACATTGGAGAGGAA  669

Query  10988  TAAATGCAAGTCCTTTTATTTTTGATAAGGACATATGTCCGAGTTAAGTTTGAAAAGGTA  11047
              ||||||||||||||||||||||||||||||||||||||||||||||||||||||||||||
Sbjct  670    TAAATGCAAGTCCTTTTATTTTTGATAAGGACATATGTCCGAGTTAAGTTTGAAAAGGTA  729

Query  11048  GCCCCAGGAGCATCAAGAGTAGATAAGGATTTAGGGTGTTAATGTACAAGCAGAATGAGT  11107
              ||||||||||||||||||||||||||||||||||||||||||||||||||||||||||||
Sbjct  730    GCCCCAGGAGCATCAAGAGTAGATAAGGATTTAGGGTGTTAATGTACAAGCAGAATGAGT  789

Query  11108  TGCAGCACTGGTTGGCACCAGAACTTGCCAGCAGCTCCAGGGCCCTGTGACTAGGAGCCA  11167
              ||||||||||||||||||||||||||||||||||||||||||||||||||||||||||||
Sbjct  790    TGCAGCACTGGTTGGCACCAGAACTTGCCAGCAGCTCCAGGGCCCTGTGACTAGGAGCCA  849

Query  11168  GCACCACTTGGAAAGTGTCAGGGCAGATGGCTTCCTGCTTGCCAGGCTGGAAAGACCAGG  11227
              |||||||||||||||||||   ||||||||||||||||||||||||||||||||||||||
Sbjct  850    GCACCACTTGGAAAGTGTCNN-GCAGATGGCTTCCTGCTTGCCAGGCTGGAAAGACCAGG  908
```

Note: shaded sequence is from backbone vector

Query: Respective Targeted Allele Sequence
Sbjct: Sequencing data from Targeting Vector

ingenious targeting laboratory

2200 Smithtown Avenue, Ronkonkoma NY 11779 ● tel: (631) 468-8530 Ext.118 ● email: alu@genetargeting.com

JENS LOX1 primer sequencing data aligned with targeted allele sequence: genomic sequence is in plain text; 5' Loxp cassette is highlighted with LoxP site underlined

```
Query  16421  TCTGACTGCAAGTATAGGCAGGAAAACGACCAGGTTGGTTCTATGTTGAGACAGAGACTA  16480
              |||| |||| |||||||||||| |||||||||||||||||||||||||||||||||||||
Sbjct  13     TCTGNCTGC-AGTATAGGCAGG-AAACGACCAGGTTGGTTCTATGTTGAGACAGAGACTA  70

Query  16481  AGGGCAGAAGAGTGAACAGATACAGACACAGGGGCCGTGGTTCTGTGGAGGGTCCTGGGG  16540
              ||||||||||||||||||||||||||||||||||||||||||||||||||||||||||||
Sbjct  71     AGGGCAGAAGAGTGAACAGATACAGACACAGGGGCCGTGGTTCTGTGGAGGGTCCTGGGG  130

Query  16541  AAGCCTTGTCCTCACTATACATTTGAAGGTCTTAGCATTATGCTTCTAAATGACTGGGCT  16600
              ||||||||||||||||||||||||||||||||||||||||||||||||||||||||||||
Sbjct  131    AAGCCTTGTCCTCACTATACATTTGAAGGTCTTAGCATTATGCTTCTAAATGACTGGGCT  190

Query  16601  AGTTTGGGGAAACACCCCAAATAACTTCGTATAATGTATGCTATACGAAGTTATGTACAA  16660
              ||||||||||||||||||||||||||||||||||||||||||||||||||||||||||||
Sbjct  191    AGTTTGGGGAAACACCCCAAATAACTTCGTATAATGTATGCTATACGAAGTTATGTACAA  250

Query  16661  ACTCCTAAATGCAATTTCACATTGACCAGAGCAAAATATTCCCTAAAAAGAATAATGACT  16720
              ||||||||||||||||||||||||||||||||||||||||||||||||||||||||||||
Sbjct  251    ACTCCTAAATGCAATTTCACATTGACCAGAGCAAAATATTCCCTAAAAAGAATAATGACT  310

Query  16721  CCAGTTTTCTGCCtttattttattttattttattttattttattttattttattgttttC  16780
              ||||||||||||||||||||||||||||||||||||||||||||||||||||||||||||
Sbjct  311    CCAGTTTTCTGCCTTTATTTTATTTTATTTTATTTTATTTTATTTTATTTTATTGTTTTC  370

Query  16781  CTTTCCTTCCCCAGATACGTGTGAGGACATTTTTATGCACAATGTGATAATTTCAGAGGG  16840
              ||||||||||||||||||||||||||||||||||||||||||||||||||||||||||||
Sbjct  371    CTTTCCTTCCCCAGATACGTGTGAGGACATTTTTATGCACAATGTGATAATTTCAGAGGG  430

Query  16841  CCAGCCTTTTCCTTTCAACTGCACATACCCGCCAGAAACAAACGGGGCAGTAAATCTGAC  16900
              ||||||||||||||||||||||||||||||||||||||||||||||||||||||||||||
Sbjct  431    CCAGCCTTTTCCTTTCAACTGCACATACCCGCCAGAAACAAACGGGGCAGTAAATCTGAC  490

Query  16901  ATGGTACAAAACACCTAGCAAAAGCCCAGTATCTAACAACAGACACCTTAGAGTTCACCA  16960
              ||||||||||||||||||||||||||||||||||||||||||||||||||||||||||||
Sbjct  491    ATGGTACAAAACACCTAGCAAAAGCCCAGTATCTAACAACAGACACCTTAGAGTTCACCA  550

Query  16961  GGACCAGACCTGGATCTTGTTTCTTCCATTGACACTGGAGGACTCCGGTATCTATCAGTG  17020
              ||||||||||||||||||||||||||||||||||||||||||||||||||||||||||||
Sbjct  551    GGACCAGACCTGGATCTTGTTTCTTCCATTGACACTGGAGGACTCCGGTATCTATCAGTG  610

Query  17021  TGTTATAAGGTAAGTCCTTCATTTAAAGTGGAACTAA-TTCCCAAGTCTCCCTCTCCATT  17079
              || ||||| | |||||||||||||||||||| ||||| ||||||||||||||||||||||
Sbjct  611    TGNTATAANGNAAGTCCTTCATTTAAAGTGGNACTAATTTCCCAAGTCTCCCTCTCCATT  670
```

Query: Respective Targeted Allele Sequence
Sbjct: Sequencing data from Targeting Vector

ingenious targeting laboratory

iNeoN2 primer sequencing data aligned with targeted allele sequence. genomic sequence is in plain text; Neo sequence is in red text with FRT site underlined;

```
Query  16762  attttattttattgttttCCTTTCCTTCCCCAGATACGTGTGAGGACATTTTTATGCACA  16821
              |||||||||||  ||||||||||| |||||||||||||||||||||||||||||||||||
Sbjct  661    ATTTTATTTTA-NGTTTTCCTTTCNTTCCCCAGATACGTGTGAGGACATTTTTATGCACA  603

Query  16822  ATGTGATAATTTCAGAGGGCCAGCCTTTTCCTTTCAACTGCACATACCCGCCAGAAACAA  16881
              ||||||||||||||||||||||||||||||||||||||||||||||||||||||||||||
Sbjct  602    ATGTGATAATTTCAGAGGGCCAGCCTTTTCCTTTCAACTGCACATACCCGCCAGAAACAA  543

Query  16882  ACGGGGCAGTAAATCTGACATGGTACAAAACACCTAGCAAAAGCCCAGTATCTAACAACA  16941
              ||||||||||||||||||||||||||||||||||||||||||||||||||||||||||||
Sbjct  542    ACGGGGCAGTAAATCTGACATGGTACAAAACACCTAGCAAAAGCCCAGTATCTAACAACA  483

Query  16942  GACACCTTAGAGTTCACCAGGACCAGACCTGGATCTTGTTTCTTCCATTGACACTGGAGG  17001
              ||||||||||||||||||||||||||||||||||||||||||||||||||||||||||||
Sbjct  482    GACACCTTAGAGTTCACCAGGACCAGACCTGGATCTTGTTTCTTCCATTGACACTGGAGG  423

Query  17002  ACTCCGGTATCTATCAGTGTGTTATAAGGTAAGTCCTTCATTTAAAGTGGAACTAATTCC  17061
              ||||||||||||||||||||||||||||||||||||||||||||||||||||||||||||
Sbjct  422    ACTCCGGTATCTATCAGTGTGTTATAAGGTAAGTCCTTCATTTAAAGTGGAACTAATTCC  363

Query  17062  CAAGTCTCCCTCTCCATTTCTAAAGACAACTTTTTAAGAAAATGGGTATTATTGGCCTTT  17121
              ||||||||||||||||||||||||||||||||||||||||||||||||||||||||||||
Sbjct  362    CAAGTCTCCCTCTCCATTTCTAAAGACAACTTTTTAAGAAAATGGGTATTATTGGCCTTT  303

Query  17122  ATTTTGGAGACTTGTTATATAGCCTAGACAAGTTTCCAACTGCTTTCTAAAGTACTGAAA  17181
              ||||||||||||||||||||||||||||||||||||||||||||||||||||||||||||
Sbjct  302    ATTTTGGAGACTTGTTATATAGCCTAGACAAGTTTCCAACTGCTTTCTAAAGTACTGAAA  243

Query  17182  GGACAGGTGTGAACCACCACAACCAGCCGCTACACACCACAACCAGCTGCTACACAAATG  17241
              ||||||||||||||||||||||||||||||||||||||||||||||||||||||||||||
Sbjct  242    GGACAGGTGTGAACCACCACAACCAGCCGCTACACACCACAACCAGCTGCTACACAAATG  183

Query  17242  CGTACGTTCGTGGGATTGTGTCCGTGTCGCGAAGTTCCTATACTTTCTAGAGAATAGGAA  17301
              ||||||||||||||||||||||||||||||||||||||||||||||||||||||||||||
Sbjct  182    CGTACGTTCGTGGGATTGTGTCCGTGTCGCGAAGTTCCTATACTTTCTAGAGAATAGGAA  123

Query  17302  CTTCCCGCGGTTGTAAGTTCTCCAGATCTAGAGTAAGACAGAAGTCTGGAAGACATGGGA  17361
              ||||||||||||||||||||||||||||||||||||||||||||||||||||||||||||
Sbjct  122    CTTCCCGCGGTTGTAAGTTCTCCAGATCTAGAGTAAGACAGAAGTCTGGAAGACATGGGA  63

Query  17362  GTGAGTTGTCAGGTGATCCAGGAAGAGACCTTCTGCAATCCAGTGACCA  17410
              ||||||||||||||||||||||||||||| | ||||| ||||| || ||
Sbjct  62     GTGAGTTGTCAGGTGATCCAGGAAGAGACNT-CTGCA-TCCAGNGANCA  16
```

Query: Respective Targeted Allele Sequence
Sbjct: Sequencing data from Targeting Vector

2200 Smithtown Avenue, Ronkonkoma NY 11779 ● tel: (631) 468-8530 Ext.118 ● email: alu@genetargeting.com

IV-NeoN3 primer sequencing data aligned with targeted allele sequence: Neo sequence is in red text with FRT site underlined; Lox71 site highlighted in yellow; Sa.P2A-mCherry.pA inversion cassette is in blue text.

```
Query  19781  GGACGCTCGCTGCGCCCTTCGTCTGACGTGGCAGCGCTCGCCGTGAGGAGGGGGGCGCCC  19840
              ||||||||||||||||||||||||||||||||||||||||||||||||||||||||||||
Sbjct  27     GGACGCTCGCTGCGCCCTTCGTCTGACGTGGCAGCGCTCGCCGTGAGGAGGGGGGCGCCC  86

Query  19841  GCGGGAGGCGCCAAAACCCGGCGCGGAGGCCGCATGCTCCAGACTGCCTTGGGAAAAGCG  19900
              ||||||||||||||||||||||||||||||||||||||||||||||||||||||||||||
Sbjct  87     GCGGGAGGCGCCAAAACCCGGCGCGGAGGCCGCATGCTCCAGACTGCCTTGGGAAAAGCG  146

Query  19901  CCTCCCCTACCCGGTAGAATGAAGTTCCTATACTTTCTAGAGAATAGGAACTTCGTTGGT  19960
              ||||||||||||||||||||||||||||||||||||||||||||||||||||||||||||
Sbjct  147    CCTCCCCTACCCGGTAGAATGAAGTTCCTATACTTTCTAGAGAATAGGAACTTCGTTGGT  206

Query  19961  ACCGTACGCAATTGTACCGTTCGTATAATGTATGCTATACGAAGTTATCTAGCGCGCCTC  20020
              ||||||||||||||||||||||||||||||||||||||||||||||||||||||||||||
Sbjct  207    ACCGTACGCAATTGTACCGTTCGTATAATGTATGCTATACGAAGTTATCTAGCGCGCCTC  266

Query  20021  GACTCTAGCCCCCTCGACTAGAGGATCGAGCCCCAGCTGGTTCTTTCCGCCTCAGAAGCC  20080
              ||||||||||||||||||||||||||||||||||||||||||||||||||||||||||||
Sbjct  267    GACTCTAGCCCCCTCGACTAGAGGATCGAGCCCCAGCTGGTTCTTTCCGCCTCAGAAGCC  326

Query  20081  ATAGAGCCCACCGCATCCCCAGCATGCCTGCTATTGTCTTCCCAATCCTCCCCCTTGCTG  20140
              ||||||||||||||||||||||||||||||||||||||||||||||||||||||||||||
Sbjct  327    ATAGAGCCCACCGCATCCCCAGCATGCCTGCTATTGTCTTCCCAATCCTCCCCCTTGCTG  386

Query  20141  TCCTGccccaccccaccccccAGAATAGAATGACACCTACTCAGACAATGCGATGCAATT  20200
              ||||||||||||||||||||||||||||||||||||||||||||||||||||||||||||
Sbjct  387    TCCTGCCCCACCCCACCCCCCAGAATAGAATGACACCTACTCAGACAATGCGATGCAATT  446

Query  20201  TCCTCATTTTATTAGGAAAGGACAGTGGGAGTGGCACCTTCCAGGGTCAAGGAAGGCACG  20260
              ||||||||||||||||||||||||||||||||||||||||||||||||||||||||||||
Sbjct  447    TCCTCATTTTATTAGGAAAGGACAGTGGGAGTGGCACCTTCCAGGGTCAAGGAAGGCACG  506

Query  20261  GGGGAGGGGCAAACAACAGATGGCTGGCAACTAGAAGGCACAGTCGAGGCTGATCAGCGA  20320
              ||||||||||||||||||||||||||||||||||||||||||||||||||||||||||||
Sbjct  507    GGGGAGGGGCAAACAACAGATGGCTGGCAACTAGAAGGCACAGTCGAGGCTGATCAGCGA  566

Query  20321  GCTCACTTGTACAGCTCGTCCATGCCGCCGGTGGAGTGGCGGCCCT-CGGCGCGT-TCGT  20378
              ||||| | |||  |||||| ||||  | |||||||||||||||||| |||||||| ||||
Sbjct  567    GCTCANTNGTANNGCTCGTACATGNGGTCGGTGGAGTGGCGGCCCTACGGCGCGTNTCGT  626
```

Query: Respective Targeted Allele Sequence
Sbjct: Sequencing data from Targeting Vector

ingenious targeting laboratory

mCherry SQ1 primer sequencing data aligned with targeted allele sequence: Sa.P2A-mCherry.pA inversion cassette is in blue text.

```
Query  20124  AATCCTCCCCTTGCTGTCCTGccccaccccacccccccAGAATAGAATGACACCTACTCA  20183
              |||||||||||||||||| |||||||||||||||||||||||||||||||||||||||||
Sbjct  732    AATCCTCCCCTTGCTGTCNTGCCCCACCCCACCCCCCCAGAATAGAATGACACCTACTCA  673

Query  20184  GACAATGCGATGCAATTTCCTCATTTTATTAGGAAAGGACAGTGGGAGTGGCACCTTCCA  20243
              ||||||||||||||||||||||||||||||||||||||||||||||||||||||||||||
Sbjct  672    GACAATGCGATGCAATTTCCTCATTTTATTAGGAAAGGACAGTGGGAGTGGCACCTTCCA  613

Query  20244  GGGTCAAGGAAGGCACGGGGGAGGGGCAAACAACAGATGGCTGGCAACTAGAAGGCACAG  20303
              ||||||||||||||||||||||||||||||||||||||||||||||||||||||||||||
Sbjct  612    GGGTCAAGGAAGGCACGGGGGAGGGGCAAACAACAGATGGCTGGCAACTAGAAGGCACAG  553

Query  20304  TCGAGGCTGATCAGCGAGCTCACTTGTACAGCTCGTCCATGCCGCCGGTGGAGTGGCGGC  20363
              ||||||||||||||||||||||||||||||||||||||||||||||||||||||||||||
Sbjct  552    TCGAGGCTGATCAGCGAGCTCACTTGTACAGCTCGTCCATGCCGCCGGTGGAGTGGCGGC  493

Query  20364  CCTCGGCGCGTTCGTACTGTTCCACGATGGTGTAGTCCTCGTTGTGGGAGGTGATGTCCA  20423
              ||||||||||||||||||||||||||||||||||||||||||||||||||||||||||||
Sbjct  492    CCTCGGCGCGTTCGTACTGTTCCACGATGGTGTAGTCCTCGTTGTGGGAGGTGATGTCCA  433

Query  20424  ACTTGATGTTGACGTTGTAGGCGCCGGGCAGCTGCACGGGCTTCTTGGCCTTGTAGGTGG  20483
              ||||||||||||||||||||||||||||||||||||||||||||||||||||||||||||
Sbjct  432    ACTTGATGTTGACGTTGTAGGCGCCGGGCAGCTGCACGGGCTTCTTGGCCTTGTAGGTGG  373

Query  20484  TCTTGACCTCAGCGTCGTAGTGGCCGCCGTCCTTCAGCTTCAGCCTCTGCTTGATCTCGC  20543
              ||||||||||||||||||||||||||||||||||||||||||||||||||||||||||||
Sbjct  372    TCTTGACCTCAGCGTCGTAGTGGCCGCCGTCCTTCAGCTTCAGCCTCTGCTTGATCTCGC  313

Query  20544  CCTTCAGGGCGCCGTCCTCGGGGTACATCCGCTCGGAGGAGGCCTCCCAGCCCATGGTCT  20603
              ||||||||||||||||||||||||||||||||||||||||||||||||||||||||||||
Sbjct  312    CCTTCAGGGCGCCGTCCTCGGGGTACATCCGCTCGGAGGAGGCCTCCCAGCCCATGGTCT  253

Query  20604  TCTTCTGCATTACGGGGCCGTCGGAGGGGAAGTTGGTGCCGCGCAGCTTCACCTTGTAGA  20663
              ||||||||||||||||||||||||||||||||||||||||||||||||||||||||||||
Sbjct  252    TCTTCTGCATTACGGGGCCGTCGGAGGGGAAGTTGGTGCCGCGCAGCTTCACCTTGTAGA  193

Query  20664  TGAACTCGCCGTCCTGCAGGGAGGAGTCCTGGGTCACGGTCACCACGCCGCCGTCCTCGA  20723
              ||||||||||||||||||||||||||||||||||||||||||||||||||||||||||||
Sbjct  192    TGAACTCGCCGTCCTGCAGGGAGGAGTCCTGGGTCACGGTCACCACGCCGCCGTCCTCGA  133

Query  20724  AGTTCATCACGCGCTCCCACTTGAAGCCCTCGGGGAAGGACAGCTTCAAGTAGTCGGGGA  20783
              ||||||||||||||||||||||||||||||||||||||||||||||||||||||||||||
Sbjct  132    AGTTCATCACGCGCTCCCACTTGAAGCCCTCGGGGAAGGACAGCTTCAAGTAGTCGGGGA  73

Query  20784  TGTCGGCGGGGTGCTTCACGTAGGCCTTGGAGCCGTACATGAACTGAGGGGACAGG  20839
              |||||||||||||||||||||||||||||||||||||||||||||||||| |||||
Sbjct  72     TGTCGGCGGGGTGCTTCACGTAGGCCTTGGAGCCGTACATGAACTGAGGG-ACAGG  18
```

Query: Respective Targeted Allele Sequence
Sbjct: Sequencing data from Targeting Vector

mCherry SQ2 primer sequencing data aligned with targeted allele sequence: Sa.P2A-mCherry.pA inversion cassette is in blue text; Lox66 cassette sequence is highlighted in yellow with Lox66 site underlined; genomic sequence is in plain text.

```
Query  20787  CGGCGGGGTGCTTCACGTAGGCCTTGGAGCCGTACATGAACTGAGGGGACAGGATGTCCC  20846
              ||||||||||||||||||||||||||||||||||||||||||||||||||||||||||||
Sbjct  19     CGGCGGGGTGCTTCACGTAGGCCTTGGAGCCGTACATGAACTGAGGGGACAGGATGTCCC  78

Query  20847  AGGCGAAGGGCAGGGGGCCACCCTTGGTCACCTTCAGCTTGGCGGTCTGGGTGCCCTCGT  20906
              ||||||||||||||||||||||||||||||||||||||||||||||||||||||||||||
Sbjct  79     AGGCGAAGGGCAGGGGGCCACCCTTGGTCACCTTCAGCTTGGCGGTCTGGGTGCCCTCGT  138

Query  20907  AGGGGCGGCCCTCGCCCTCGCCCTCGATCTCGAACTCGTGGCCGTTCACGGAGCCCTCCA  20966
              ||||||||||||||||||||||||||||||||||||||||||||||||||||||||||||
Sbjct  139    AGGGGCGGCCCTCGCCCTCGCCCTCGATCTCGAACTCGTGGCCGTTCACGGAGCCCTCCA  198

Query  20967  TGTGCACCTTGAAGCGCATGAACTCCTTGATGATGGCCATGTTATCCTCCTCGCCCTTGC  21026
              ||||||||||||||||||||||||||||||||||||||||||||||||||||||||||||
Sbjct  199    TGTGCACCTTGAAGCGCATGAACTCCTTGATGATGGCCATGTTATCCTCCTCGCCCTTGC  258

Query  21027  TCACCATAGGTCCAGGGTTCTCCTCCACGTCTCCAGCCTGCTTCAGCAGGCTGAAGTTAG  21086
              ||||||||||||||||||||||||||||||||||||||||||||||||||||||||||||
Sbjct  259    TCACCATAGGTCCAGGGTTCTCCTCCACGTCTCCAGCCTGCTTCAGCAGGCTGAAGTTAG  318

Query  21087  TAGCTCCGCTTCCGTCCTCACACGTATCTGGGGAAGGAAAGGaaaacaataaaataaaat  21146
              ||||||||||||||||||||||||||||||||||||||||||||||||||||||||||||
Sbjct  319    TAGCTCCGCTTCCGTCCTCACACGTATCTGGGGAAGGAAAGGAAAACAATAAAATAAAAT  378

Query  21147  aaaataaaataaaataaaataaaataaaGGCAGAAAACTGGAGTCATTATTCTTTTTAGG  21206
              ||||||||||||||||||||||||||||||||||||||||||||||||||||||||||||
Sbjct  379    AAAATAAAATAAAATAAAATAAAATAAAGGCAGAAAACTGGAGTCATTATTCTTTTTAGG  438

Query  21207  GAATATTTTGCTCTGGTCAATGTGAAATTGCATTTAGGAGTTTGTACCGTTCGTATAGCA  21266
              ||||||||||||||||||||||||||||||||||||||||||||||||||||||||||||
Sbjct  439    GAATATTTTGCTCTGGTCAATGTGAAATTGCATTTAGGAGTTTGTACCGTTCGTATAGCA  498

Query  21267  TACATTATACGAAGTTATACGCGTAGGGCAGCCTTGAACTGGCAGGAGATGACGTCCAGT  21326
              ||||||||||||||||||||||||||||||||||||||||||||||||||||||||||||
Sbjct  499    TACATTATACGAAGTTATACGCGTAGGGCAGCCTTGAACTGGCAGGAGATGACGTCCAGT  558

Query  21327  GAGCACTTTTGAAAGCAGAACAGCTTTAGAAATAACTTTTGGAGCACAGATTGCACCATC  21386
              ||||||||||||||||||||||||||||||||||||||||||||||||||||||||||||
Sbjct  559    GAGCACTTTTGAAAGCAGAACAGCTTTAGAAATAACTTTTGGAGCACAGATTGCACCATC  618

Query  21387  TTCTTCATTGAAGTTTTAGCCCAGACCAGCTTACAGAGAGAGGTGTTTTATCAATGAGAT  21446
              ||||||||||||||||||||||||||||||||||||||||||||||||||||||||||||
Sbjct  619    TTCTTCATTGAAGTTTTAGCCCAGACCAGCTTACAGAGAGAGGTGTTTTATCAATGAGAT  678

Query  21447  TGCTGCCATTTCCGAAGATGGCTAATGGTTTTGGAAAACTGGCTGATCCAGACCCAA-CT  21505
              ||||||||||||||||||||||||||||||||||||||||||||||||||||||||| ||
Sbjct  679    TGCTGCCATTTCCGAAGATGGCTAATGGTTTTGGAAAACTGGCTGATCCAGACCCAATCT  738
```

Query: Respective Targeted Allele Sequence
Sbjct: Sequencing data from Targeting Vector

ingenious targeting laboratory

JENS SQ2 primer sequencing data aligned with targeted allele sequence: Sa.P2A-mCherry.pA inversion cassette is in blue text; Lox66 cassette sequence is highlighted in yellow with Lox66 site underlined; genomic sequence is in plain text.

```
Query  20806  GGCCTTGGAGCC-GTACATGAACTGAGGGG-ACAGGATGTCCCAGGCGAAGGCAGGGGG  20863
              |||||||||||| ||||||||||||||||| ||||||||||||||||||||||||||||
Sbjct  775    GGCCTTGGAGCCCGTACATGAACTGAGGGGGACAGGATGTCCCAGGCGAAGGCAGGGGG  716

Query  20864  CCACCCTTGGTCACCTTCAGCTTGGCGGTCTGGGTGCCCTCGTAGGGGCGGCCCTCGCCC  20923
              ||||||||||||||||||||||||||||||||||||||||||||||||||||||||||||
Sbjct  715    CCACCCTTGGTCACCTTCAGCTTGGCGGTCTGGGTGCCCTCGTAGGGGCGGCCCTCGCCC  658

Query  20924  TCGCCCTCGATCTCGAACTCGTGGCCGTTCACGGAGCCCTCCATGTGCACCTTGAAGCGC  20983
              ||||||||||||||||||||||||||||||||||||||||||||||||||||||||||||
Sbjct  657    TCGCCCTCGATCTCGAACTCGTGGCCGTTCACGGAGCCCTCCATGTGCACCTTGAAGCGC  598

Query  20984  ATGAACTCCTTGATGATGGCCATGTTATCCTCCTCGCCCTTGCTCACCATAGGTCCAGGG  21043
              ||||||||||||||||||||||||||||||||||||||||||||||||||||||||||||
Sbjct  597    ATGAACTCCTTGATGATGGCCATGTTATCCTCCTCGCCCTTGCTCACCATAGGTCCAGGG  538

Query  21044  TTCTCCTCCACGTCTCCAGCCTGCTTCAGCAGGCTGAAGTTAGTAGCTCCGCTTCCGTCC  21103
              ||||||||||||||||||||||||||||||||||||||||||||||||||||||||||||
Sbjct  537    TTCTCCTCCACGTCTCCAGCCTGCTTCAGCAGGCTGAAGTTAGTAGCTCCGCTTCCGTCC  478

Query  21104  TCACACGTATCTGGGGAAGGAAAGGaaaacaataaaataaaataaaataaaataaaataa  21163
              ||||||||||||||||||||||||||||||||||||||||||||||||||||||||||||
Sbjct  477    TCACACGTATCTGGGGAAGGAAAGGAAAACAATAAAATAAAATAAAATAAAATAAAATAA  418

Query  21164  aataaaataaaGGCAGAAAACTGGAGTCATTATTCTTTTTAGGGAATATTTTGCTCTGGT  21223
              ||||||||||||||||||||||||||||||||||||||||||||||||||||||||||||
Sbjct  417    AATAAAATAAAGGCAGAAAACTGGAGTCATTATTCTTTTTAGGGAATATTTTGCTCTGGT  358

Query  21224  CAATGTGAAATTGCATTTAGGAGTTTGTACCGTTCGTATAGCATACATTATACGAAGTTA  21283
              ||||||||||||||||||||||||||||||||||||||||||||||||||||||||||||
Sbjct  357    CAATGTGAAATTGCATTTAGGAGTTTGTACCGTTCGTATAGCATACATTATACGAAGTTA  298

Query  21284  TACGCGTAGGGCAGCCTTGAACTGGCAGGAGATGACGTCCAGTGAGCACTTTTGAAAGCA  21343
              ||||||||||||||||||||||||||||||||||||||||||||||||||||||||||||
Sbjct  297    TACGCGTAGGGCAGCCTTGAACTGGCAGGAGATGACGTCCAGTGAGCACTTTTGAAAGCA  238

Query  21344  GAACAGCTTTAGAAATAACTTTTGGAGCACAGATTGCACCATCTTCTTCATTGAAGTTTT  21403
              ||||||||||||||||||||||||||||||||||||||||||||||||||||||||||||
Sbjct  237    GAACAGCTTTAGAAATAACTTTTGGAGCACAGATTGCACCATCTTCTTCATTGAAGTTTT  178

Query  21404  AGCCCAGACCAGCTTACAGAGAGAGGTGTTTTATCAATGAGATTGCTGCCATTTCCGAAG  21463
              ||||||||||||||||||||||||||||||||||||||||||||||||||||||||||||
Sbjct  177    AGCCCAGACCAGCTTACAGAGAGAGGTGTTTTATCAATGAGATTGCTGCCATTTCCGAAG  118

Query  21464  ATGGCTAATGGTTTTGGAAAACTGGCTGATCCAGACCCAACTAGATCATTCAAACACATC  21523
              ||||||||||||||||||||||||||||||||||||||||||||||||||||||||||||
Sbjct  117    ATGGCTAATGGTTTTGGAAAACTGGCTGATCCAGACCCAACTAGATCATTCAAACACATC  58

Query  21524  TGATAAGGAATCAGCTTAAGGCAGACATTTGAAAGCTAGCAAATCTGGG  21572
              ||||||||||||||||||||||||||||| ||||||||| ||| |||||
Sbjct  57     TGATAAGGAATCAGCTTAAGGCAGACATT-GAAAGCTAGNAAANCTGGG  10
```

Query: Respective Targeted Allele Sequence
Sbjct: Sequencing data from Targeting Vector

T73 primer sequencing data aligned with respective targeted allele sequence

```
Query  23100  ACA-CAAATTCTTG-CCTAAGAAAGACTCCTC-AGGGC-TATCTGTGTTC-TGTTACTCA  23154
              ||| |||||||||| ||||||||||  ||||| ||||| |||||| |||| |||||||||
Sbjct  298    ACACCAAATTCTTGCCCTAAGAAAGNNTCCTCCAGGGCTTATCTGNGTTCTTGTTACTCA  239

Query  23155  -CCAAACCTTGTCCCCAGCCATGGCTGCTGCTATCAACTACTGCCACCCTCCTCCCGCCA  23213
               |||||||||||||||||||||||||||||||||||||||||||||||||||||||||||
Sbjct  238    CCCAAACCTTGTCCCCAGCCATGGCTGCTGCTATCAACTACTGCCACCCTCCTCCCGCCA  179

Query  23214  CTCCCCACCTCAGTATTTGAAAACTCAGTTGTATCTTAGCCTCTTCTCTTTGAAATGTGA  23273
              ||||||||||||||||||||||||||||||||||||||||||||||||||||||||||||
Sbjct  178    CTCCCCACCTCAGTATTTGAAAACTCAGTTGTATCTTAGCCTCTTCTCTTTGAAATGTGA  119

Query  23274  ACAGTTTCCCATGCTCCTCTTTCTTCATCCTGTGTTTGCTTTAACCTGGCCTTCTCTCCT  23333
              ||||||||||||||||||||||||||||||||||||||||||||||||||||||||||||
Sbjct  118    ACAGTTTCCCATGCTCCTCTTTCTTCATCCTGTGTTTGCTTTAACCTGGCCTTCTCTCCT  59

Query  23334  CAAGGCTCAGCTAGACACT  23352
              |||||||||||||||||||
Sbjct  58     CAAGGCTCAGCTAGACACT  40
```

Query: Respective Targeted Allele Sequence
Sbjct: Sequencing data from Targeting Vector

ingenious targeting laboratory

Backbone Vector Sequence

3’end of BAC subclone joins here

catCGATGATGGGCCACATTGGCCTCGACGATATCGCGATCGCCGATAAGCCAGGTTAACCTGCATTAACGCGCCGTCGACGCGGCGCG
TTTGCGTATTGGGCGCTCTTCCGCTTCCTCGCTCACTGACTCGCTGCGCTCGGTCGTTCGGCTGCGGCGAGCGGTATCAGCTCACTCAA
AGGCGGTAATACGGTTATCCACAGAATCAGGGGATAACGCAGGAAAGAACATGTGAGCAAAAGGCCAGCAAAAGGCCAGGAACCGTAAA
AAGGCCGCGTTGCTGGCGTTTTTCCATAGGCTCCGCCCCCCTGACGAGCATCACAAAAATCGACGCTCAAGTCAGAGGTGGCGAAACCC
GACAGGACTATAAAGATACCAGGCGTTTCCCCCTGGAAGCTCCCTCGTGCGCTCTCCTGTTCCGACCCTGCCGCTTACCGGATACCTGT
CCGCCTTTCTCCCTTCGGGAAGCGTGGCGCTTTCTCATAGCTCACGCTGTAGGTATCTCAGTTCGGTGTAGGTCGTTCGCTCCAAGCTG
GGCTGTGTGCACGAACCCCCCGTTCAGCCCGACCGCTGCGCCTTATCCGGTAACTATCGTCTTGAGTCCAACCCGGTAAGACACGACTT
ATCGCCACTGGCAGCAGCCACTGGTAACAGGATTAGCAGAGCGAGGTATGTAGGCGGTGCTACAGAGTTCTTGAAGTGGTGGCCTAACT
ACGGCTACACTAGAAGAACAGTATTTGGTATCTGCGCTCTGCTGAAGCCAGTTACCTTCGGAAAAAGAGTTGGTAGCTCTTGATCCGGC
AAACAAACCACCGCTGGTAGCGGTGGTTTTTTTGTTTGCAAGCAGCAGATTACGCGCAGAAAAAAAGGATCTCAAGAAGATCCTTTGAT
CTTTTCTACGGGGTCTGACGCTCAGTGGAACGAAAACTCACGTTAAGGGATTTTGGTCATGAGATTATCAAAAAGGATCTTCACCTAGA
TCCTTTTAAATTAAAAATGAAGTTTTAAATCAATCTAAAGTATATATGAGTAAACTTGGTCTGACAGTTACCAATGCTTAATCAGTGAG
GCACCTATCTCAGCGATCTGTCTATTTCGTTCATCCATAGTTGCCTGACTCCCCGTCGTGTAGATAACTACGATACGGGAGGGCTTACC
ATCTGGCCCCAGTGCTGCAATGATACCGCGAGACCCACGCTCACCGGCTCCAGATTTATCAGCAATAAACCAGCCAGCCGGAAGGGCCG
AGCGCAGAAGTGGTCTGCAACTTTATCCGCCTCCATCCAGTCTATTAATTGTTGCCGGGAAGCTAGAGTAAGTAGTTCGCCAGTTAATA
GTTTGCGCAACGTTGTTGCCATTGCTACAGGCATCGTGGTGTCACGCTCGTCGTTTGGTATGGCTTCATTCAGCTCCGGTTCCCAACGA
TCAAGGCGAGTTACATGATCCCCCATGTTGTGCAAAAAAGCGGTTAGCTCCTTCGGTCCTCCGATCGTTGTCAGAAGTAAGTTGGCCGC
AGTGTTATCACTCATGGTTATGGCAGCACTGCATAATTCTCTTACTGTCATGCCATCCGTAAGATGCTTTTCTGTGACTGGTGAGTACT
CAACCAAGTCATTCTGAGAATAGTGTATGCGGCGACCGAGTTGCTCTTGCCCGGCGTCAATACGGGATAATACCGCGCCACATAGCAGA
ACTTTAAAAGTGCTCATCATTGGAAAACGTTCTTCGGGGCGAAAACTCTCAAGGATCTTACCGCTGTTGAGATCCAGTTCGATGTAACC
CACTCGTGCACCCAACTGATCTTCAGCATCTTTTACTTTCACCAGCGTTTCTGGGTGAGCAAAAACAGGAAGGCAAAATGCCGCAAAAA
AGGGAATAAGGGCGACACGGAAATGTTGAATACTCATACTCTTCCTTTTTCAATATTATTGAAGCATTTATCAGGGTTATTGTCTCATG
AGCGGATACATATTTGAATGTATTTAGAAAAATAAACAAATAGGGGTTCCGCGCACATTTCCCCGAAAAGTGCCACCTGACGTCTAAGA
AACCATTATTATCATGACATTAACCTATAAAAATAGGCGTATCACGAGGCCCTTTCGTCTCGCGCGTTTCGGTGATGACGGTGAAAACC
TCTGACACATGCAGCTCCCGGAGACGGTCACAGCTTGTCTGTAAGCGGATGCCGGGAGCAGACAAGCCCGTCAGGGCGCGTCAGCGGGT
GTTGGCGGGTGTCGGGGCTGGCTTAACTATGCGGCATCAGAGCAGATTGTACTGAGAGTGCACCATATGGACATATTGTCGTTAGAACG
CGGCTACAATTAATACATAACCTTATGTATCATACACATACGATTTAGGTGACACTATAGAACTCGATGCGGCCCCTGCAGGCGCGCCA
TTTAAATGCGGCCGCACCTCAGGATGTCCCCTGAAGct

5’ end of BAC subclone joins here

2200 Smithtown Avenue, Ronkonkoma NY 11779 ● tel: (631) 468-8530 Ext.118 ● email: alu@genetargeting.com

3. Cassette Sequences

FRT flanked IV-UBS-Neo Cassette (3'-5')

FRT sites are ***Bold/Italics***
ATG and **STOP** codons are indicated

GTACGTTCGTGGGATTGTGTCCGTGTCGC***GAAGTTCCTATACTTTCTAGAGAATAGGAACTTC***CCGCGGTTGTAAGTTCTCCAGATCTA
GAGTAAGACAGAAGTCTGGAAGACATGGGAGTGAGTTGTCAGGTGATCCAGGAAGAGACCTTCTGCAATCCAGTGACCAATTAATTACA
GCAGAAAGGACCATCGGGAAGGAAAGCCATACTCTCCAGGAACGTCATTAGTCGGGATCTTCAGTTGCTACAAGAAGCAGATGTCAAAC
GGCCTTCCCCTAACCATGTGAGAAGTGAGCTTTCACTGGCCCGGGTGTGAAGTGATTCTAATGGAATAAATGGATTTGCTAAGGAATAG
TTTCCTCAGAAGAAATCCTGGGAGCAAGTGGGGAAAGCTGACTCAGCAAAACAGAGCTGTTTCTTGAGGACGATGCCAATAGCAATCAT
TTGACCAAACTGAAGTGGCCGTCAGGAGGCATGAGGATCTGATATCAGGGAGCTCTCAGACGTCGCTTGGTCGGTCTTTATTCGAACCC
CAGAGTCCCGC**TCA**GAAGAACTCGTCAAGAAGGCGATAGAAGGCGATGCGCTGCGAATCGGGAGCGGCGATACCGTAAAGCACGAGGAA
GCGGTCAGCCCATTCGCCGCCAAGCTCTTCAGCAATATCACGGGTAGCCAACGCTATGTCCTGATAGCGGTCCGCCACACCCAGCCGGC
CACAGTCGATGAATCCAGAAAAGCGGCCATTTTCCACCATGATATTCGGCAAGCAGGCATCGCCATGGGTCACGACGAGATCCTCGCCG
TCGGGCATGCGCGCCTTGAGCCTGGCGAACAGTTCGGCTGGCGCGAGCCCCTGATGCTCTTCGTCCAAATCATCCTGATCGACAAGACC
GGCTTCCATCCGTGTCCGTGCTCGCTCGATGCGATGTTTCGCTTGGTGGTCGAATGGGCAGGTAGCCGGATCAAGCGTATGCAGCCGCC
GCATTGCATCAGCCATGATGGATACTTTCTCGGCCGGAGCAAGATGAGATGACAGGAGATCCTGCCCCGGCACTTCGCCCAATAGCAGC
CAGTCCCTTCCCGCTTCCGTGACAACGTCGAGCACAGCTGCGCAAGGAACGCCCGTCGTGGCCAGCCACGATAGCCGCGCTGCCTCGTC
CTGCAATTCATTCAAGGCACCGGATAGGTCGGTCTTGACAAAAAGAACCGGGCGCCCCTGCGCTGACAGCCGGAACACGGCGGCATCAG
AGCAGCCGATCGTCTGTTGTGCCCAGTCATAGCCGAATAGCCTCTCCACCCAAGCGGCCGGAGAACCTGCGTGCAATCCATCTTGTTCA
ATGGCCGATCC**CAT**GGTTTAGTTCCTCACCTTGTCGTATTATACTATGCCGATATACTATGCCGATGATTAATTGTCAACACGTCTAAC
AAAAAAGCCAAAAACGGCCAGAATTTAGCGGACAATTTACTAGTCTAACACTGAAAATTACATATTGACCCAAATGATTACATTTCAAA
AGGTGCCTAAAAAACTTCACAAAACACACTCGCCAACCCCGAGCGCATAGTTCAAAACCGGAGCTTCAGCTACTTAAGAAGATACGTAC
ATAAAACCGACCAAAGAAACTGACGCCTCACTTATCCCTCCCCTCACCCGAGGTCCGGCGCCTGTCGATTCAGGAGAGCCTACCCTAGG
CCCGAACCCTGCGTCCTGCGACGGAGAAAAGCCTACCGCACACCTACCGGCAGGTGGCCCCACCCTGCATTATAAGCCAACAGAACGGG
TGACGTCACGACACGACGAGGGCGCGCGCTCCCAAAGTTACGGGTGCACTGCCCAACGGCACCGCCATAACTGCCGCCCCCGCAACAGA
CGACAAACCGAGTTCTCCAGTCAGTGACAAACTTCACGTCAGGGTCCCCAGATGGTGCCCCAGCCCATCTCACCCGAATAAGAGCTTTC
CCGCATTAGCGAAGGCCTCAAGACCTTGGGTTCTTGCCGCCCACCATGCCCCCCACCTTGTTTCAACGACCTCACAGCCCGCCTCACAA
GCGTCTTCCATTCAAGACTCGGGAACAGCCGCCATTTTGCTGCGCTCCCCCCAACCCCCAGTTCAGGGCAACCTTGCTCGCGGACCCAG
ACTACAGCCCTTGGCGGTCTCTCCACACGCTTCCGTCCCACCGAGCGGCCCGGCGGCCACGAAAGCCCCGGCCAGCCCAGCAGCCCGCT
ACTCACCAAGTGACGATCACAGCGATCCACAAACAAGAACTGCGACCCAAATCCCGGCTGCGACGGAACTAGCTGTGCCACACCCGGCG
CGTCCTTATATAATCATCGGCGTTCACCGCCCCACGGAGATCCCTCCGCAGAATCGCCGAGAAGGGACTACTTTTCCTCGCCTGTTCCG
CTCTCTGGAAAGAAAACCAGTGCCCTAGAGTCACCCAAGTCCCGTCCTAAAATGTCCTTCTGCTGATACTGGGGTTCTAAGGCCGAGTC
TTATGAGCAGCGGGCCGCTGTCCTGAGCGTCCGGGCGGAAGGATCAGGACGCTCGCTGCGCCCTTCGTCTGACGTGGCAGCGCTCGCCG
TGAGGAGGGGGGCGCCCGCGGGAGGCGCCAAAACCCGGCGCGGAGGCCGCATGCTCCAGACTGCCTTGGGAAAAGCGCCTCCCCTACCC
GGTAGAAT***GAAGTTCCTATACTTTCTAGAGAATAGGAACTTC***GTTGGTACCGTACGCAATTG
