# Supplementary material for: Floxed Il1rl2 Locus with mCherry Reporter Element Reveals Distinct Expression Patterns of the IL-36 Receptor in Barrier Tissues
Source: Cells. 2024 May 6;13(9):787. doi: 10.3390/cells13090787 (PMC11083296; doi:10.3390/cells13090787)

# JENS-B: Screening and Reconfirmation of Recombinant Clones

## Sections:

1. PCR Screening Strategy
2. Reconfirmation of Expanded Clones by PCR
3. Confirmation of 3’ Cassette Retention by DNA Sequencing
4. Confirmation of 5’ Distal LoxP Retention by DNA Sequencing
5. Analysis of Gene Targeting by Real-time PCR
6. References

## 1. PCR Screening Strategy

Ten micrograms of the targeting vector was linearized and then transfected by electroporation of FLP C57Bl/6 (BF1) embryonic stem cells. After selection with G418 antibiotic, surviving clones were expanded for PCR analysis to identify recombinant ES clones. The Neo cassette in the targeting vector was removed during ES clone expansion.

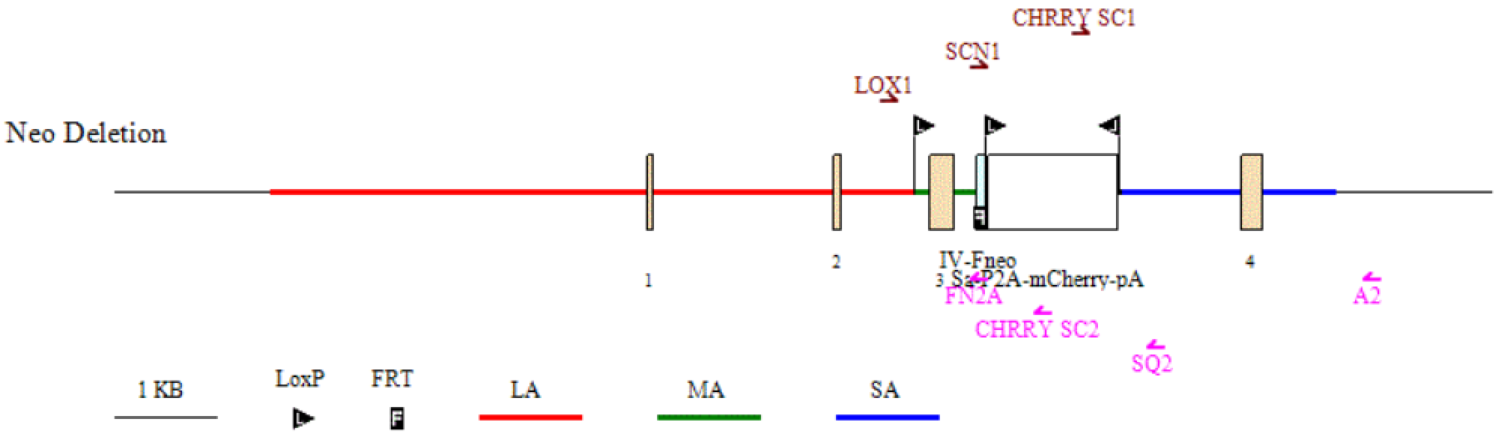


### Primers for PCR Screening

```
A2:         5’- ACC TTC AAG GAC CTG TGT CAT TCC   -3’
CHRRY SC1:  5’- CAC CCT TGG TCA CCT TCA GCT TGG   -3’
SQ2:        5’- CAT TAC CTC TGG CAC ATG GAT TC    -3’
SCN1:       5’- CGT ACG TTC GTG GGA TTG TGT CC    -3’
CHRRY SC2:  5’- AAG CAG AGG CTG AAG CTG AAG GAC   -3’
LOX1:       5’- AGG GAA GCT GTC TTT AGA ACC AAG C -3’
FN2A:       5’- AAC TTC GCG ACA CGG ACA CAA TCC   -3’
```

Screening primer A2 was designed downstream of the short homology arm (SA) outside the 3’ region used to generate the targeting construct. PCR reactions using A2 with the CHRRY SC1 primer amplify 2.82 kb fragment. Clones 163, 251, 323, 372, and 374 were identified as positive and selected for further expansion.

ingenious targeting laboratory

## 2. Reconfirmation of Expanded Clones by PCR

Clones 163, 251, 323, 372, and 374 were expanded and reconfirmed for SA integration. An “x” denotes expanded clones. DNA from an individual clone (before expansion) was used as a positive control and denoted as a (+). No DNA was used as a negative control, and denoted by a (--). Wild Type DNA was used as a negative control, and denoted by a (wt).

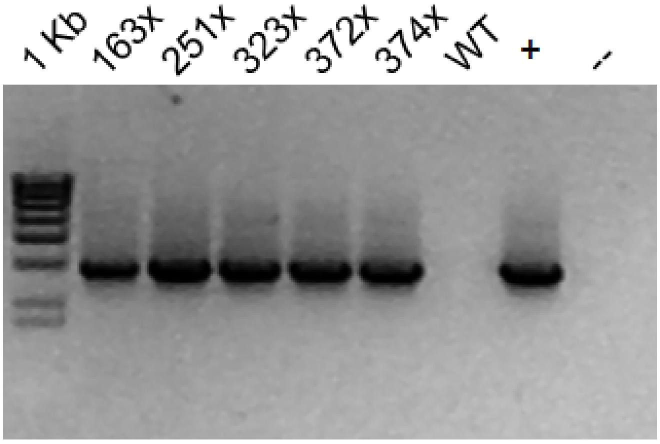


**JENS-B A2/ CHRRY SC1 (2.82 KB)**

### 3. Confirmation of 3’ Cassette Retention by DNA Sequencing

Confirmation of 3’ cassette retention was performed by PCR using the SQ2 and SCN1 primers.  This reaction produces a product 1.73 kb in size.

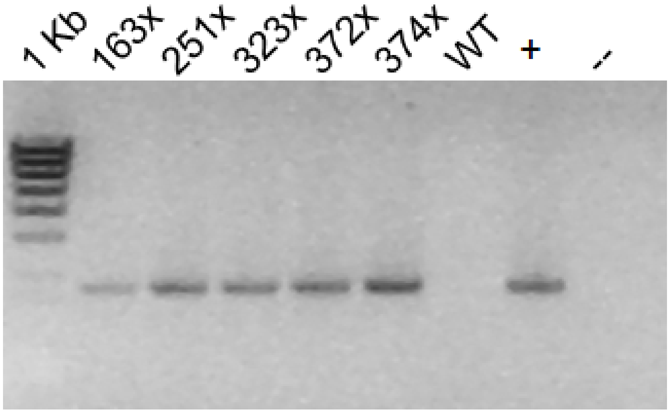


**JENS-B SQ2/ SCN1 (1.73 KB)**

Sequencing was performed on purified PCR DNA to confirm the Sa-P2A-mCherry-pA/ genome junction using the CHRRY SC1 primer. The sequence from a confirmed clone is shown below (Query = sequence from clone #374; Sbjct = vector sequence).

```
Query  21     GCCCTCGCCCTCGATCTCGAACTCGTGGCCGTTCACGGAGCCCTCCATGTGCACCTTGAA  80
              ||||||||||||||||||||||||||||||||||||||||||||||||||||||||||||
Sbjct  18271  GCCCTCGCCCTCGATCTCGAACTCGTGGCCGTTCACGGAGCCCTCCATGTGCACCTTGAA  18330

Query  81     GCGCATGAACTCCTTGATGATGGCCATGTTATCCTCCTCGCCCTTGCTCACCATAGGTCC  140
              ||||||||||||||||||||||||||||||||||||||||||||||||||||||||||||
Sbjct  18331  GCGCATGAACTCCTTGATGATGGCCATGTTATCCTCCTCGCCCTTGCTCACCATAGGTCC  18390

Query  141    AGGGTTCTCCTCCACGTCTCCAGCCTGCTTCAGCAGGCTGAAGTTAGTAGCTCCGCTTCC  200
              ||||||||||||||||||||||||||||||||||||||||||||||||||||||||||||
Sbjct  18391  AGGGTTCTCCTCCACGTCTCCAGCCTGCTTCAGCAGGCTGAAGTTAGTAGCTCCGCTTCC  18450

Query  201    GTCCTCACACGTATCTGGGGAAGGAAAGGaaaacaataaaataaaataaaataaaataaa  260
              ||||||||||||||||||||||||||||||||||||||||||||||||||||||||||||
Sbjct  18451  GTCCTCACACGTATCTGGGGAAGGAAAGGAAAACAATAAAATAAAATAAAATAAAATAAA  18510

Query  261    ataaaataaaataaaGGCAGAAAACTGGAGTCATTATTCTTTTTAGGGAATATTTTGCTC  320
              ||||||||||||||||||||||||||||||||||||||||||||||||||||||||||||
Sbjct  18511  ATAAAATAAAATAAAGGCAGAAAACTGGAGTCATTATTCTTTTTAGGGAATATTTTGCTC  18570

Query  321    TGGTCAATGTGAAATTGCATTTAGGAGTTTGTATAACTTCGTATAGCATACATTATACGA  380
              ||||||||||||||||||||||||||||||||||||||||||||||||||||||||||||
Sbjct  18571  TGGTCAATGTGAAATTGCATTTAGGAGTTTGTATAACTTCGTATAGCATACATTATACGA  18630

Query  381    ACGGTACGCGTAGGGCAGCCTTGAACTGGCAGGAGATGACGTCCAGTGAGCACTTTTGAA  440
              ||||||||||||||||||||||||||||||||||||||||||||||||||||||||||||
Sbjct  18631  ACGGTACGCGTAGGGCAGCCTTGAACTGGCAGGAGATGACGTCCAGTGAGCACTTTTGAA  18690

Query  441    AGCAGAACAGCTTTAGAAATAACTTTTGGAGCACAGATTGCACCATCTTCTTCATTGAAG  500
              ||||||||||||||||||||||||||||||||||||||||||||||||||||||||||||
Sbjct  18691  AGCAGAACAGCTTTAGAAATAACTTTTGGAGCACAGATTGCACCATCTTCTTCATTGAAG  18750

Query  501    TTTTAGCCCAGACCAGCTTACAGAGAGAGGTGTTTTATCAATGAGATTGCTGCCATTTCC  560
              ||||||||||||||||||||||||||||||||||||||||||||||||||||||||||||
Sbjct  18751  TTTTAGCCCAGACCAGCTTACAGAGAGAGGTGTTTTATCAATGAGATTGCTGCCATTTCC  18810

Query  561    GAAGATGGCTAATGGTTTTGGAAAACTGGCTGATCCAGACCCAACTAGATCATTCAAACA  620
              ||||||||||||||||||||||||||||||||||||||||||||||||||||||||||||
Sbjct  18811  GAAGATGGCTAATGGTTTTGGAAAACTGGCTGATCCAGACCCAACTAGATCATTCAAACA  18870

Query  621    CATCTGATAAGGAATCAGCTTAAGGCAGACATTTGAAAGCTAGCAAATCTGGGAGATGTT  680
              ||||||||||||||||||||||||||||||||||||||||||||||||||||||||||||
Sbjct  18871  CATCTGATAAGGAATCAGCTTAAGGCAGACATTTGAAAGCTAGCAAATCTGGGAGATGTT  18930

Query  681    CTAGTGTCTACATGAATCCATGT  703
              |||||||||||||||||||||||
Sbjct  18931  CTAGTGTCTACATGAATCCATGT  18953
```

Sa-P2A-mCherry-pA cassette sequence is in orange text; Lox71 is highlighted cyan; genomic sequence is in plain text.

Sequencing was performed on purified PCR DNA to confirm Sa-P2A-mCherry-pA/ Neo cassette junction using the CHRRY SC2 primer. The sequence from a confirmed clone is shown below (Query = sequence from clone #374; Sbjct = vector sequence).

```
Query  19     GTC-AGACCACCTACAAGGCCAAGAAGCCCGTGCAGCTGCCCGGCGCCTACAACGTCAAC  77
              ||| ||||||||||||||||||||||||||||||||||||||||||||||||||||||||
Sbjct  17841  GTCAAGACCACCTACAAGGCCAAGAAGCCCGTGCAGCTGCCCGGCGCCTACAACGTCAAC  17782

Query  78     ATCAAGTTGGACATCACCTCCCACAACGAGGACTACACCATCGTGGAACAGTACGAACGC  137
              ||||||||||||||||||||||||||||||||||||||||||||||||||||||||||||
Sbjct  17781  ATCAAGTTGGACATCACCTCCCACAACGAGGACTACACCATCGTGGAACAGTACGAACGC  17722

Query  138    GCCGAGGGCCGCCACTCCACCGGCGGCATGGACGAGCTGTACAAGTGAGCTCGCTGATCA  197
              ||||||||||||||||||||||||||||||||||||||||||||||||||||||||||||
Sbjct  17721  GCCGAGGGCCGCCACTCCACCGGCGGCATGGACGAGCTGTACAAGTGAGCTCGCTGATCA  17662

Query  198    GCCTCGACTGTGCCTTCTAGTTGCCAGCCATCTGTTGTTTGCCCCTCCCCCGTGCCTTCC  257
              ||||||||||||||||||||||||||||||||||||||||||||||||||||||||||||
Sbjct  17661  GCCTCGACTGTGCCTTCTAGTTGCCAGCCATCTGTTGTTTGCCCCTCCCCCGTGCCTTCC  17602

Query  258    TTGACCCTGGAAGGTGCCACTCCCACTGTCCTTTCCTAATAAAATGAGGAAATTGCATCG  317
              ||||||||||||||||||||||||||||||||||||||||||||||||||||||||||||
Sbjct  17601  TTGACCCTGGAAGGTGCCACTCCCACTGTCCTTTCCTAATAAAATGAGGAAATTGCATCG  17542

Query  318    CATTGTCTGAGTAGGTGTCATTCTATTCTggggggtggggtggggCAGGACAGCAAGGGG  377
              ||||||||||||||||||||||||||||||||||||||||||||||||||||||||||||
Sbjct  17541  CATTGTCTGAGTAGGTGTCATTCTATTCTGGGGGGTGGGGTGGGGCAGGACAGCAAGGGG  17482

Query  378    GAGGATTGGGAAGACAATAGCAGGCATGCTGGGGATGCGGTGGGCTCTATGGCTTCTGAG  437
              ||||||||||||||||||||||||||||||||||||||||||||||||||||||||||||
Sbjct  17481  GAGGATTGGGAAGACAATAGCAGGCATGCTGGGGATGCGGTGGGCTCTATGGCTTCTGAG  17422

Query  438    GCGGAAAGAACCAGCTGGGGCTCGATCCTCTAGTCGAGGGGGCTAGAGTCGAGGCGCGCT  497
              ||||||||||||||||||||||||||||||||||||||||||||||||||||||||||||
Sbjct  17421  GCGGAAAGAACCAGCTGGGGCTCGATCCTCTAGTCGAGGGGGCTAGAGTCGAGGCGCGCT  17362

Query  498    AGTACCGTTCGTATAGCATACATTATACGAAGTTATCAATTGCGTACGGTACCAACGAAG  557
              ||||||||||||||||||||||||||||||||||||||||||||||||||||||||||||
Sbjct  17361  AGTACCGTTCGTATAGCATACATTATACGAAGTTATCAATTGCGTACGGTACCAACGAAG  17302

Query  558    TTCCTATTCTCTAGAAAGTATAGGAACTTCGCGACACGGACACAATCCC  606
              |||||||||||||||||||||||||||||||||||||||||||||||||
Sbjct  17301  TTCCTATTCTCTAGAAAGTATAGGAACTTCGCGACACGGACACAATCCC  17253
```

Sa-P2A-mCherry-pA cassette sequence is in orange text; Lox 66 sequence is highlighted green; the remaining Neo cassette sequence is in red text.

2200 Smithtown Avenue, Ronkonkoma NY 11779 ● tel: (631) 468-8530 Ext.114 ● email: echen@genetargeting.com

## 4. Confirmation of 5’ Distal LoxP Retention by DNA Sequencing

Confirmation of distal LoxP retention was performed by PCR using the LOX1 and FN2A primers. This reaction produces a product 0.90 kb in size.

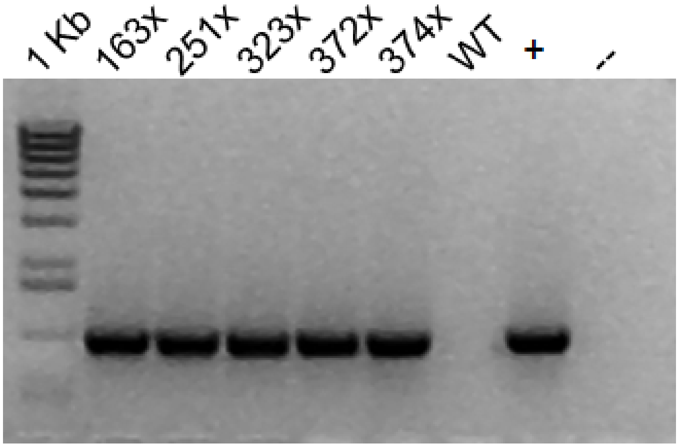


**JENS-B LOX1/ FN2A (0.90 KB)**

Sequencing was performed on purified PCR DNA to confirm presence of the distal LoxP cassette using the LOX1 primer. The sequence from a confirmed clone is shown below (Query = sequence from clone #374; Sbjct = vector sequence).

```
Query  16     TCTGNCTGC-AGTATAGGCAGG-AAACGACCAGGTTGGTTCTATGTTGAGACAGAGACTA  73
              |||| |||| |||||||||||| |||||||||||||||||||||||||||||||||||||
Sbjct  16421  TCTGACTGCAAGTATAGGCAGGAAAACGACCAGGTTGGTTCTATGTTGAGACAGAGACTA  16480

Query  74     AGGGCAGAAGAGTGAACAGATACAGACACAGGGGCCGTGGTTCTGTGGAGGGTCCTGGGG  133
              ||||||||||||||||||||||||||||||||||||||||||||||||||||||||||||
Sbjct  16481  AGGGCAGAAGAGTGAACAGATACAGACACAGGGGCCGTGGTTCTGTGGAGGGTCCTGGGG  16540

Query  134    AAGCCTTGTCCTCACTATACATTTGAAGGTCTTAGCATTATGCTTCTAAATGACTGGGCT  193
              ||||||||||||||||||||||||||||||||||||||||||||||||||||||||||||
Sbjct  16541  AAGCCTTGTCCTCACTATACATTTGAAGGTCTTAGCATTATGCTTCTAAATGACTGGGCT  16600

Query  194    AGTTTGGGGAAACACCCCAAATAACTTCGTATAATGTATGCTATACGAAGTTATGTACAA  253
              ||||||||||||||||||||||||||||||||||||||||||||||||||||||||||||
Sbjct  16601  AGTTTGGGGAAACACCCCAAATAACTTCGTATAATGTATGCTATACGAAGTTATGTACAA  16660

Query  254    ACTCCTAAATGCAATTTCACATTGACCAGAGCAAAATATTCCCTAAAAAGAATAATGACT  313
              ||||||||||||||||||||||||||||||||||||||||||||||||||||||||||||
Sbjct  16661  ACTCCTAAATGCAATTTCACATTGACCAGAGCAAAATATTCCCTAAAAAGAATAATGACT  16720

Query  314    CCAGTTTTCTGCCtttattttatttattttatttattttatttattttattgttttC  373
              ||||||||||||||||||||||||||||||||||||||||||||||||||||||||||||
Sbjct  16721  CCAGTTTTCTGCCTTTATTTTATTTATTTTATTTATTTTATTTATTTTATTGTTTTC  16780

Query  374    CTTTCCTTCCCCAGATACGTGTGAGGACATTTTTATGCACAATGTGATAATTTCAGAGGG  433
              ||||||||||||||||||||||||||||||||||||||||||||||||||||||||||||
Sbjct  16781  CTTTCCTTCCCCAGATACGTGTGAGGACATTTTTATGCACAATGTGATAATTTCAGAGGG  16840

Query  434    CCAGCCTTTTCCTTTCAACTGCACATACCCGCCAGAAACAAACGGGGCAGTAAATCTGAC  493
              ||||||||||||||||||||||||||||||||||||||||||||||||||||||||||||
Sbjct  16841  CCAGCCTTTTCCTTTCAACTGCACATACCCGCCAGAAACAAACGGGGCAGTAAATCTGAC  16900

Query  494    ATGGTACAAAACACCTAGCAAAAGCCCAGTATCTAACAACAGACACCTTAGAGTTCACCA  553
              ||||||||||||||||||||||||||||||||||||||||||||||||||||||||||||
Sbjct  16901  ATGGTACAAAACACCTAGCAAAAGCCCAGTATCTAACAACAGACACCTTAGAGTTCACCA  16960

Query  554    GGACCAGACCTGGATCTTGTTTCTTCCATTGACACTGGAGGACTCCGGTATCTATCAGTG  613
              ||||||||||||||||||||||||||||||||||||||||||||||||||||||||||||
Sbjct  16961  GGACCAGACCTGGATCTTGTTTCTTCCATTGACACTGGAGGACTCCGGTATCTATCAGTG  17020

Query  614    TGTTATAAGGTAAGTCCTTCATTTAAAGTGGAACTAATTCCCAAGTCTCCCTCTCCATTT  673
              ||||||||||||||||||||||||||||||||||||||||||||||||||||||||||||
Sbjct  17021  TGTTATAAGGTAAGTCCTTCATTTAAAGTGGAACTAATTCCCAAGTCTCCCTCTCCATTT  17080

Query  674    CTAAAGACAACTTTTTAAGAAAATGGGTATTATTGGCCTTTATTTTGGAGACTTGTTATA  733
              ||||||||||||||||||||||||||||||||||||||||||||||||||||||||||||
Sbjct  17081  CTAAAGACAACTTTTTAAGAAAATGGGTATTATTGGCCTTTATTTTGGAGACTTGTTATA  17140

Query  734    TAGCCTANACAAGTTTCCAACTGCTTTCTAAAGTACTGAAAGGACAGGTGTGAACCACCA  793
              ||||||| ||||||||||||||||||||||||||||||||||||||||||||||||||||
Sbjct  17141  TAGCCTAGACAAGTTTCCAACTGCTTTCTAAAGTACTGAAAGGACAGGTGTGAACCACCA  17200
```

Genomic sequence is in plain text; distal LoxP is highlighted yellow.

Clones 163, 251, 323, 372, and 374 were further analyzed by real-time PCR.

## 5. Analysis of Gene Targeting by Real-time PCR

### A. Gene Targeting Analysis

Analysis of clones for gene targeting using a probe which anneals to wild type allele and corresponds to the target site is shown below. The WT sample is indicated as BF1.

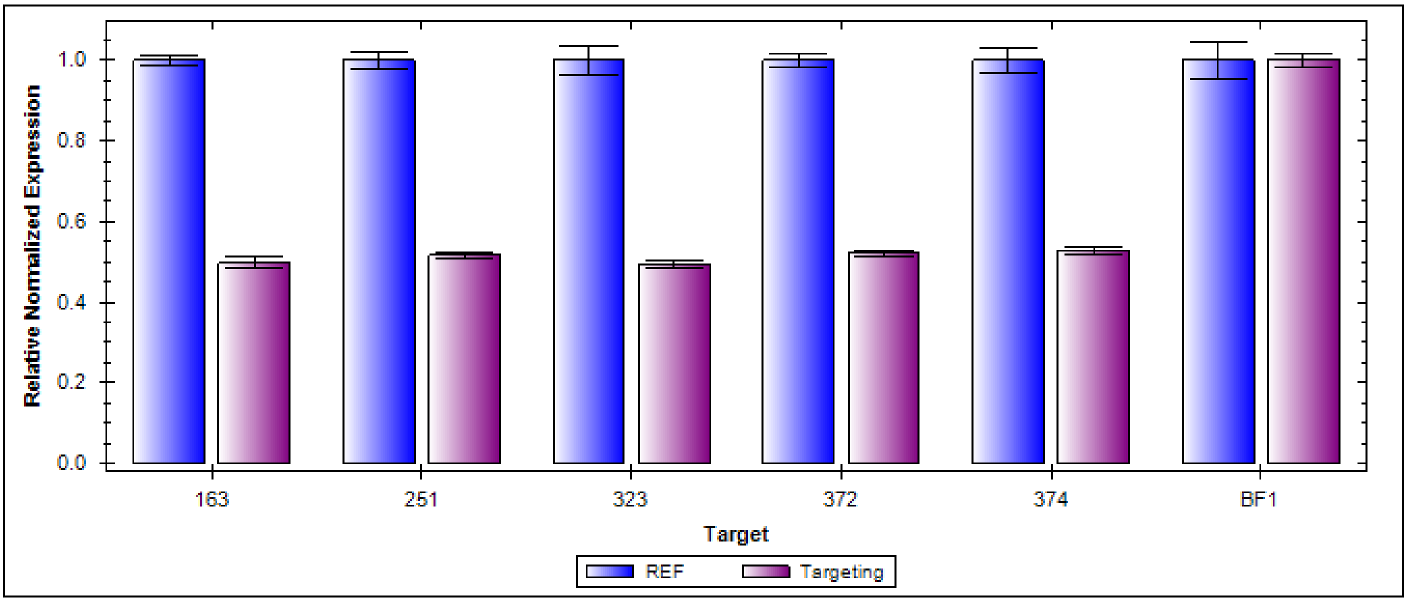


### B. Integrated Copy Number Analysis

Analysis of clones for copy number using a probe annealing to the 5' homology arm region is shown below. The WT sample is indicated as BF1.

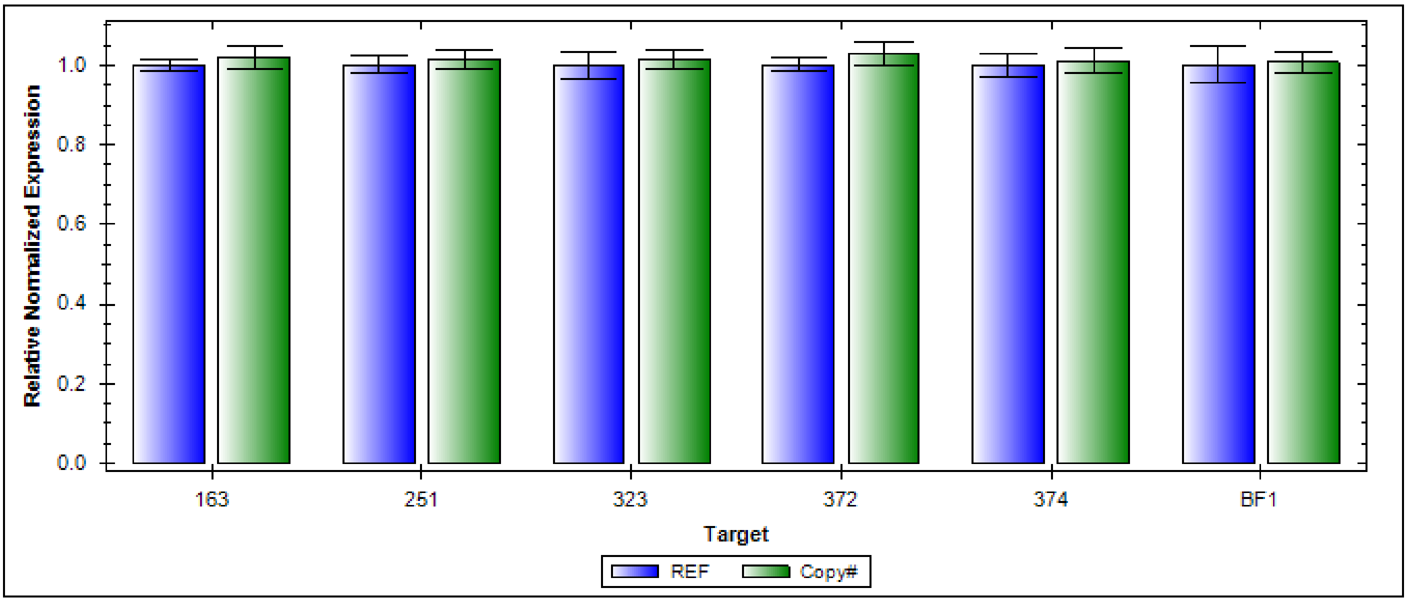


**Result:** Clones 163, 251, 323, 372, and 374 are correctly targeted and carry a single copy of the vector sequence integrated through gene targeting.

ingenious targeting laboratory

Clones 163, 251, 323, 372, and 374 are recommended for injection.

2200 Smithtown Avenue, Ronkonkoma NY 11779 ● tel: (631) 468-8530 Ext.114 ● email: echen@genetargeting.com

## 6. References

Below are references for the 1 kb and 100 bp ladders.

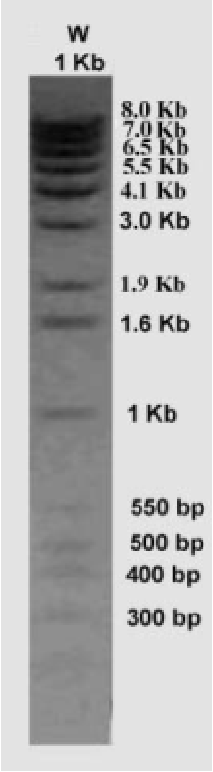


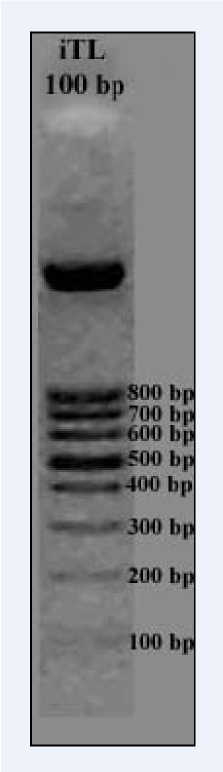

Supplement: Supplementary file 1 [file cells-13-00787-s001.zip › Supplemental file S2.pdf]
