# Supplementary material for: Floxed Il1rl2 Locus with mCherry Reporter Element Reveals Distinct Expression Patterns of the IL-36 Receptor in Barrier Tissues
Source: Cells. 2024 May 6;13(9):787. doi: 10.3390/cells13090787 (PMC11083296; doi:10.3390/cells13090787)

JENSEN: PCR Screening of Mice

<u>Sections</u>:

I. Identification of Somatic Neo Deleted Mice

1. Schematic and Information
2. Screening for Neo Deletion
3. Confirmation of Distal LoxP
4. Confirmation of Short Homology Arm Integration
5. Somatic Neo Deleted Mouse Information

II. Identification of Germline Neo Deleted Mice

1. Schematic and Information
2. Screening for Neo Deletion
3. Screening for FLP transgene
4. Germline Neo Deleted Mouse Information

III. Reference

I. Identification of Germline Neo Deleted Mice

1. Schematic and Information

Targeted iTL IN2 (C57BL/6) embryonic stem cells were microinjected into Balb/c blastocysts. Resulting chimeras with a high percentage black coat color were mated to FLP C57BL/6 mice to generate Somatic Neo Deleted mice. Tail DNA was analyzed as described below from pups with black coat color.

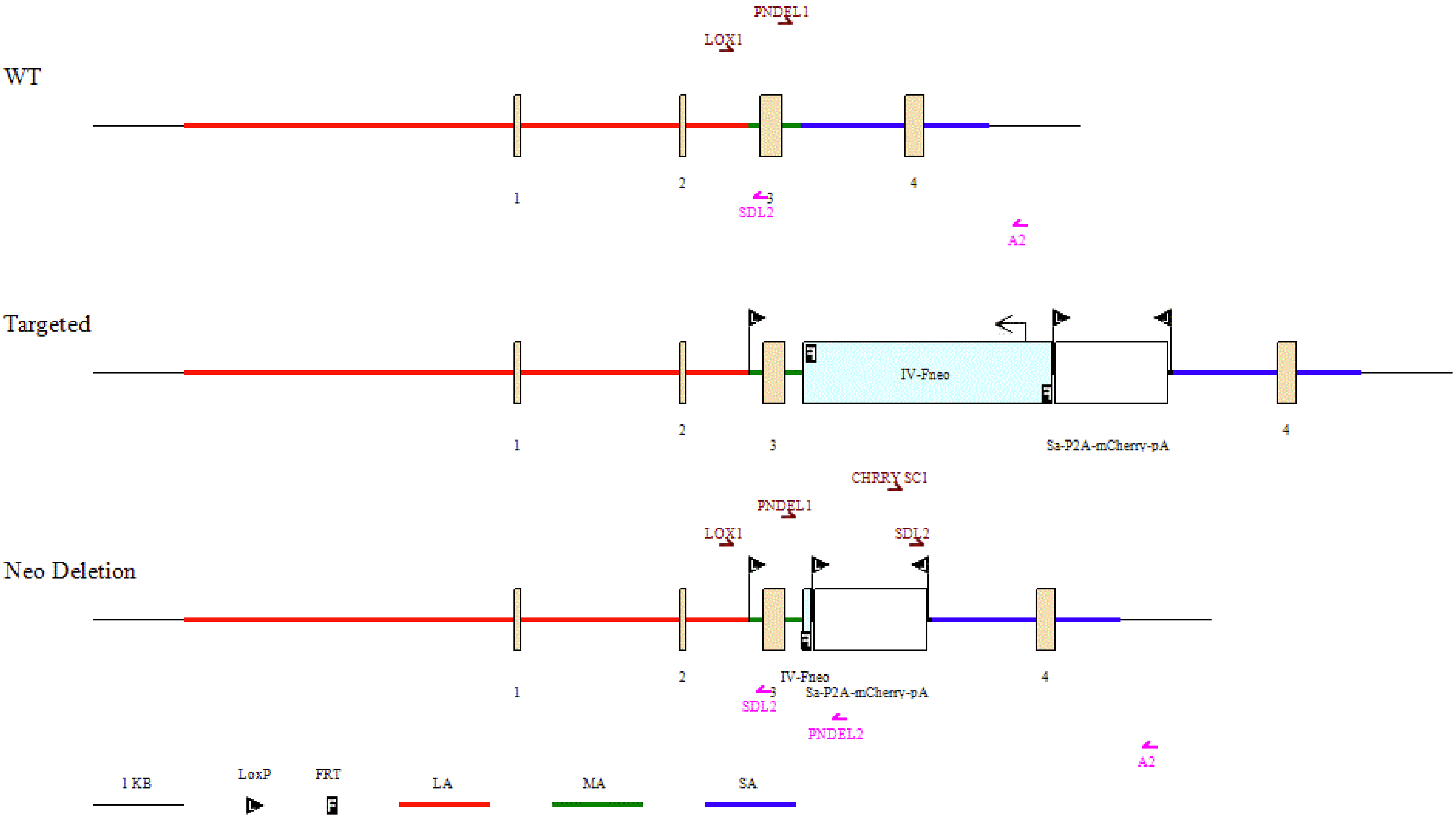

Primers for PCR Screening:

```
Forward Oligos

LOX1:       5'- AGG GAA GCT GTC TTT AGA ACC AAG C -3'
PNDEL1:     5'- TCC CAA GTC TCC CTC TCC AT        -3'
CHRRY SC1:  5'- CAC CCT TGG TCA CCT TCA GCT TGG   -3'
SDL2:       5'- CAC ACG TAT CTG GGG AAG GAA AGG   -3'

Reverse Oligos

SLD2:       5'- CAC ACG TAT CTG GGG AAG GAA AGG -3'
PNDEL2:     5'- CCA TCT GTT GTT TGC CCC TC       -3'
A2:         5'- ACC TTC AAG GAC CTG TGT CAT TCC -3'
```

2. Screening for Neo Deletion

Primer set PNDEL1 and PNDEL2 was used to screen mice for the deletion of the Neo cassette. PNDEL1 is on the long homology arm upstream of the remaining Neo Cassette, and is downstream inside the mCHRRY cassette. A 428 bp PCR product indicates Neo Deletion.

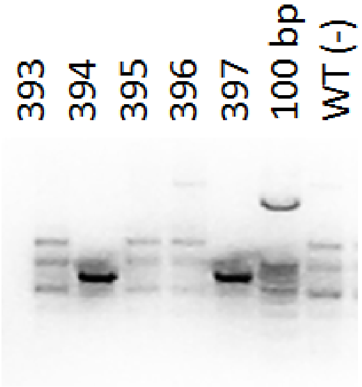

PNDEL1 / PNDEL2 (428 bp)
**Annealing temperature 58 °C*

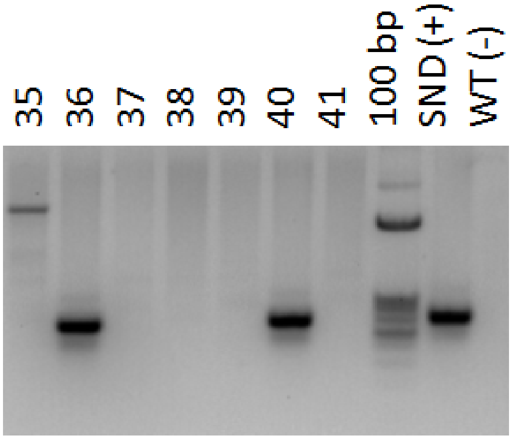

PNDEL1 / PNDEL2 (428 bp)
**Increased annealing temperature to 60 °C*

PCR Parameters for PNDEL1 / PNDEL2:

EconoTaq Plus Green 2x Master Mix (Lucigen catalog# 30033-1)

11.00 µL ddH2O
12.50 µL EconoTaq Plus Green 2x Master Mix
0.25µL 100 µM Primer
1.00 µL DNA

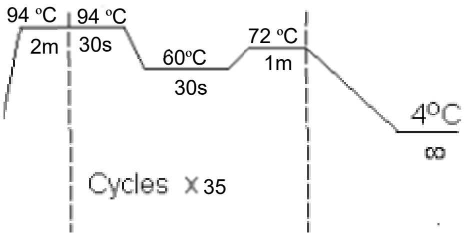

After a 2 minute hot start at 94°C the samples were run using the above conditions. The PCR product was run on a 2% gel with a 100 bp ladder as reference. The band representing Neo deletion was excised and sequenced in positive samples.

Below is sequencing of representative mouse #394 using primer PNDEL1. The sequence shows the deletion of the Neo cassette.

The remaining sequence of the Neo cassette is in red text with the FRT site underlined and the LoxP site highlighted, and the mCherry cassette is in violet text.

```
Query  18     GGGTATTATTGGCCTTTATTTTGGAGACTTGTTATATAGCCTAGACAAGTTTCCAACTGC  77
              ||||||||||||||||||||||||||||||||||||||||||||||||||||||||||||
Sbjct  17105  GGGTATTATTGGCCTTTATTTTGGAGACTTGTTATATAGCCTAGACAAGTTTCCAACTGC  17164

Query  78     TTTCTAAAGTACTGAAAGGACAGGTGTGAACCACCACAACCAGCCGCTACACACCACAAC  137
              ||||||||||||||||||||||||||||||||||||||||||||||||||||||||||||
Sbjct  17165  TTTCTAAAGTACTGAAAGGACAGGTGTGAACCACCACAACCAGCCGCTACACACCACAAC  17224

Query  138    CAGCTGCTACACAAATGCGTACGTTCGTGGGATTGTGTCCGTGTCGCGAAGTTCCTATAC  197
              ||||||||||||||||||||||||||||||||||||||||||||||||||||||||||||
Sbjct  17225  CAGCTGCTACACAAATGCGTACGTTCGTGGGATTGTGTCCGTGTCGCGAAGTTCCTATAC  17284

Query  198    TTTCTAGAGAATAGGAACTTCGTTGGTACCGTACGCAATTGATAACTTCGTATAATGTAT  257
              ||||||||||||||||||||||||||||||||||||||||||||||||||||||||||||
Sbjct  17285  TTTCTAGAGAATAGGAACTTCGTTGGTACCGTACGCAATTGATAACTTCGTATAATGTAT  17344

Query  258    GCTATACGAACGGTACTAGCGCGCCTCGACTCTAGCCCCCTCGACTAGAGGATCGAGCCC  317
              ||||||||||||||||||||||||||||||||||||||||||||||||||||||||||||
Sbjct  17345  GCTATACGAACGGTACTAGCGCGCCTCGACTCTAGCCCCCTCGACTAGAGGATCGAGCCC  17404

Query  318    CAGCTGGTTCTTTCCGCCTCAGAAGCCATAGAGCCCACCGCATCCCCAGCATGCCTGCTA  377
              ||||||||||||||||||||||||||||||||||||||||||||||||||||||||||||
Sbjct  17405  CAGCTGGTTCTTTCCGCCTCAGAAGCCATAGAGCCCACCGCATCCCCAGCATGCCTGCTA  17464

Query  378    TTGTCTTCCCAATCCTCCCCCTTGCTGTCCTGccccaccccacccccCAGAATAGAATGA  437
              ||||||||||||||||||||||||||||||||||||||||||||||||||||||||||||
Sbjct  17465  TTGTCTTCCCAATCCTCCCCCTTGCTGTCCTGCCCCACCCCACCCCCCAGAATAGAATGA  17524

Query  438    CACCTACTCAGACAATGCGATGCAATTTCCTCATTTTATTAGGAAAGGACAGTGGGAGTG  497
              ||||||||||||||||||||||||||||||||||||||||||||||||||||||||||||
Sbjct  17525  CACCTACTCAGACAATGCGATGCAATTTCCTCATTTTATTAGGAAAGGACAGTGGGAGTG  17584

Query  498    GCACCTTCCAGGGTCAAGGAAGGCACGGGGGAGGGGC  534
              |||||||||||||||||||||||||||||||||||||
Sbjct  17585  GCACCTTCCAGGGTCAAGGAAGGCACGGGGGAGGGGC  17621

Query: Sequencing data from PCR products
Sbjct: Respective targeted allele sequence
```

3. Screening for Distal LoxP Site

A PCR was performed to detect presence of the distal LoxP site using the LOX1 and SDL2 primers. This reaction amplifies a wild type product 396 bp in size. The presence of a second PCR product 32 bp greater than the wild type product indicates a positive LoxP PCR.

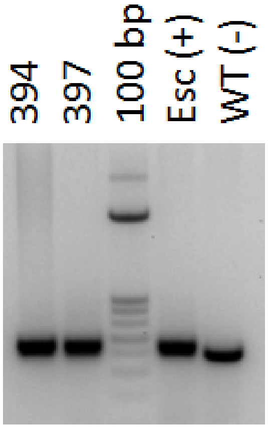

LOX1/ SDL2 (396 bp/ 428 bp)

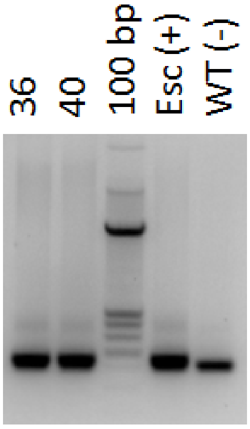

LOX1/ SDL2 (396 bp/ 428 bp)

PCR Parameters for LOX1/ SDL2:

EconoTaq Plus Green 2x Master Mix (Lucigen catalog# 30033-1)

11.00 µL ddH20
12.50 µL EconoTaq Plus Green 2x Master Mix
0.25µL 100 µM Primer
1.00 µL DNA

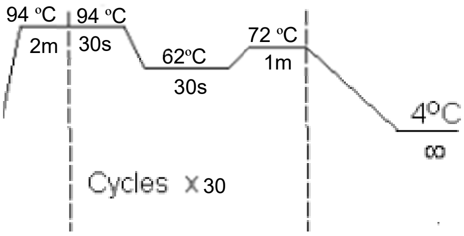

After a 2 minute hot start at 94°C the samples were run using the above conditions. The PCR product was run on a 2% gel with a 100 bp ladder as reference. The expanded ES clone, which was used as a positive control, is denoted by a (+) in the gel photograph above.

4. Confirmation of Short Homology Arm Integration

Tail DNA samples from positive mice were amplified with primers CHRRY SC1 and A2. CHRRY SC1 is located inside mCherry cassette and A2 is located downstream of the short homology arm, outside the region used to create the targeting construct. CHRRY SC1 / A2 amplifies a fragment of 3.15 kb in length.

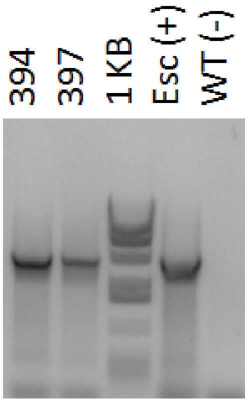

CHRRY SC1 / A2 (2.83 kb)

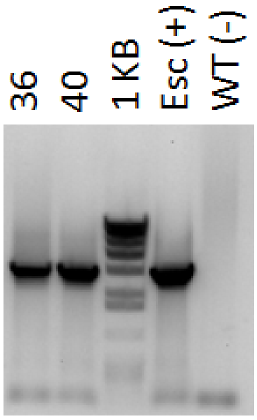

CHRRY SC1 / A2 (2.83 kb)

PCR Parameters for CHRRY SC1/ A2:

Expand High Fidelity PCR System (Roche catalog # 04 738 276 001)

17.50 µL ddH20
2.50 µL 200.0 µM dNTP
2.50 µL PCR Buffer with 15mM $MgCl_2$
1.00 µL DMSO
0.25 µL 100 µM Each Primer
1.00 µL 1.5 µL DNA

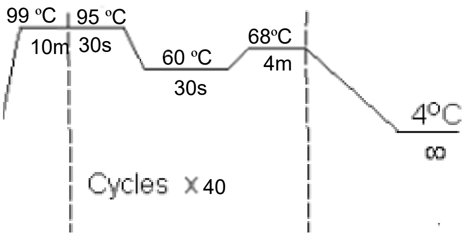

After a 10 minute hot start at 99°C, 0.125 µL of Taq polymerase was added to each PCR sample followed by a layer of 2 drops mineral oil. The PCR product was run on a 0.8% gel with a 1 KB ladder as reference. The expanded ES clone, which was used as a positive control, is denoted by a (+) in the gel photograph above.

5. Somatic Neo Deleted Mouse Information

The following mice were confirmed for Somatic Neo Deletion.

Mouse #	Sex	DOB	Clone #	Parent Info
394	M	07/07/2020	281/364	CH X FLP C57BL/6
397	F	07/07/2020	281/364	CH X FLP C57BL/6
36	M	08/04/2020	281/364	CH X FLP C57BL/6
40	F	08/04/2020	281/364	CH X FLP C57BL/6

****Asterisked mouse #394 will be retained at ingenious facility as backup.**

II. Identification of Germline Neo Deleted Mice

1. Schematic and Information

Confirmed Somatic Neo Deleted mice were set-up for mating with WT C57BL/6N mice to generate Germline Neo Deleted mice. Tail DNA from offpspring was analyzed as described below.

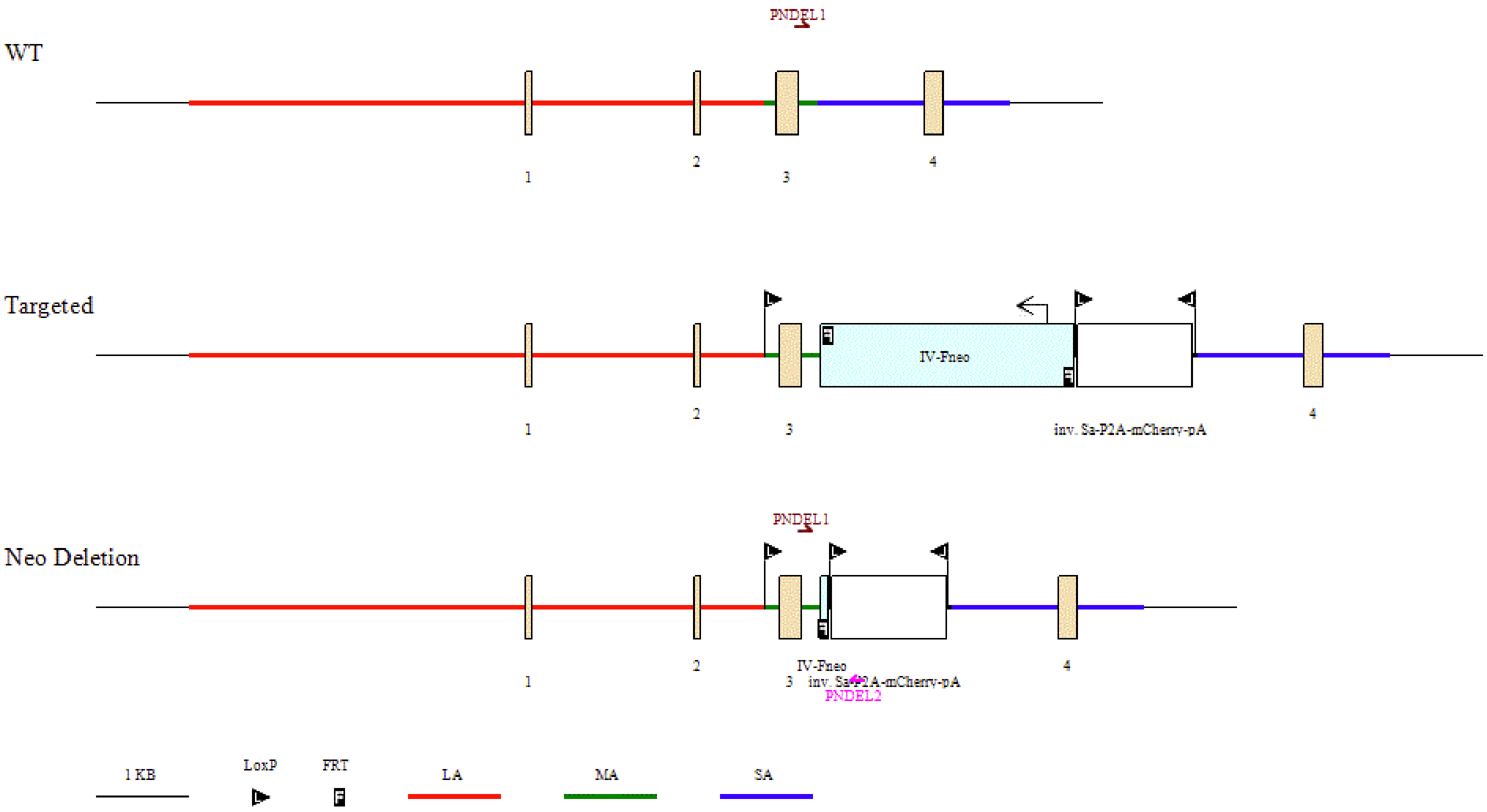

Primers for PCR Screening:

Forward Oligos

PNDEL1: 5'- TCC CAA GTC TCC CTC TCC AT -3'

Reverse Oligos

PNDEL2: 5'- CCA TCT GTT GTT TGC CCC TC -3'

newFLP1: 5'- ACA GAG ACA AAG ACA AGC GTT AGT AGG -3'
newFLP2: 5'- ATT TCC CAC AAC ATT AGT CAA CTC CGT TAG G-3'

*The FLP primers cannot be seen in the schematic above.

2. Screening for Neo Deletion

Primer set PNDEL1 and PNDEL2 was used to screen mice for the deletion of the Neo cassette. PNDEL1 is on the long homology arm upstream of the remaining Neo Cassette, and is downstream inside the mCHRRY cassette. A 428 bp PCR product indicates Neo Deletion.

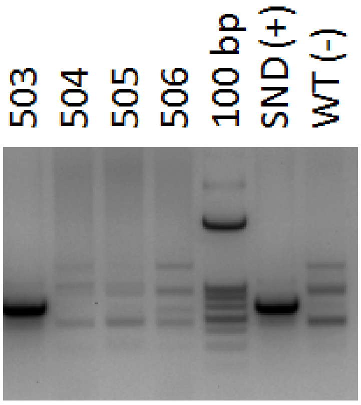

PNDEL1 / PNDEL2 (428 bp)

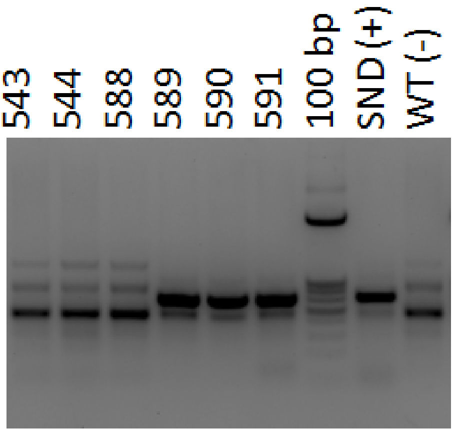

PNDEL1 / PNDEL2 (428 bp)

PCR Parameters for PNDEL1 / PNDEL2:

EconoTaq Plus Green 2x Master Mix (Lucigen catalog# 30033-1)

11.00 µL ddH2O
12.50 µL EconoTaq Plus Green 2x Master Mix
0.25µL 100 µM Primer
1.00 µL DNA

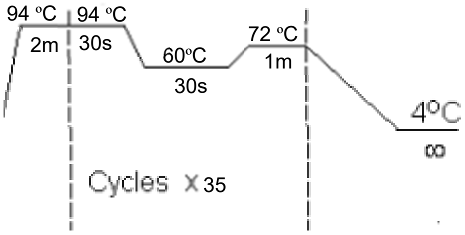

After a 2 minute hot start at 94ºC the samples were run using the above conditions. The PCR product was run on a 2% gel with a 100 bp ladder as reference. The band representing Neo deletion was excised and sequenced in positive samples.

3. Screening for FLP Transgene

Primer set newFLP1 and newFLP2 was used to screen mice for the FLP transgene. The amplified product for primer set newFLP1 and newFLP2 is 330bp.

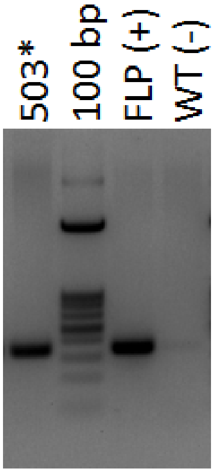

newFLP1 / newFLP2 (330 bp if FLP transgene present)
(*Asterisked mice are FLP present)

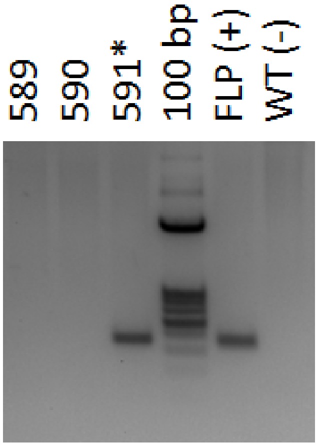

newFLP1 / newFLP2 (330 bp if FLP transgene present)
(*Asterisked mice are FLP present)

PCR Parameters for newFLP1 / newFLP2:

EconoTaq Plus Green 2x Master Mix (Lucigen catalog# 30033-1)

11 µL ddH2O
12.5 µL EconoTaq Plus Green 2x Master Mix
.25µL 100 µM Primer
1.0 µL DNA

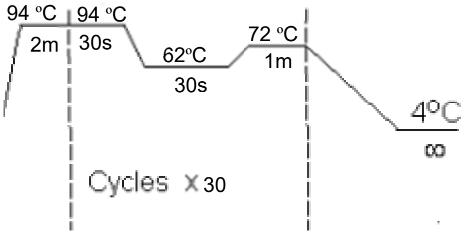

After a 2 minute hot start at 94ºC the samples were run using the above conditions. The PCR product was run on a 2% gel with a 100 bp ladder as reference. Tail DNA sample from a FLP mouse was used as a positive control and is denoted by a (+) in the gel photographs.

4. Germline Neo Deleted Mouse Information

The following mice were confirmed for Germline Neo Deletion.

Mouse #	Sex	DOB	Clone #	Parent Info
503*	M	09/23/2020	281/364	SND #394 X WT C57BL/6N
589	M	09/23/2020	281/364	SND #397 X WT C57BL/6N
590	M	09/23/2020	281/364	SND #397 X WT C57BL/6N
591*	M	09/23/2020	281/364	SND #397 X WT C57BL/6N

***Asterisked mice contain the FLP transgene, which are recommended to mate with WT mice to breed out the FLP transgene.**

III. Reference

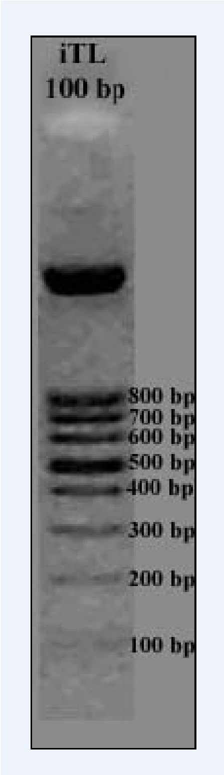

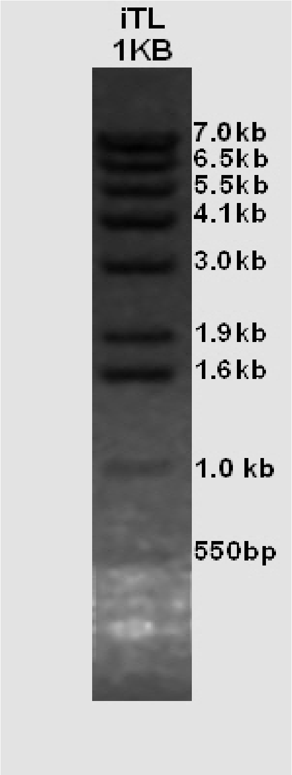

Supplement: Supplementary file 1 [file cells-13-00787-s001.zip › Supplemental file S3.pdf]
