# Supplementary material for: Floxed Il1rl2 Locus with mCherry Reporter Element Reveals Distinct Expression Patterns of the IL-36 Receptor in Barrier Tissues
Source: Cells. 2024 May 6;13(9):787. doi: 10.3390/cells13090787 (PMC11083296; doi:10.3390/cells13090787)
Supplement: Supplementary file 1 [file cells-13-00787-s001.zip › Supplemental file S4.pdf]

ingenious targeting laboratory

JENSEN *Il1rl12* Knockin Allele (Neo Deleted)

1. Outside Long Arm (Outside LA) (10451 bp): Plain text (e.g., ATCG)
2. Outside Short Arm (Outside SA) (13681 bp): Plain text (e.g., ATCG)
3. Long Arm (LA) (6173 bp): Bold and italic text (e.g., ***ATCG***)
4. Middle Arm (MA) (586 bp): Green text (e.g., ATCG)
5. Remaining Neo Cassette Sequence (83 bp): Red text (e.g., ATCG)
6. Sa.P2A-mCherry.pA(1243 bp): BlueViolet text, (e.g., ATCG)
7. Distal LoxP (39 bp): Lightgray text, highlighted in yellow (e.g., ATCG)
8. Short Arm (SA) (2062 bp): Underlined text (e.g., ATCG)
9. LoxP: Red text, highlighted in yellow (e.g., ATCG)
10. FRT: Underlined in red (e.g., ATCG)
11. Exon (mouse): Highlighted in pink (e.g., ATCG)
12. Oligo (forward): Highlighted in lime (e.g., ATCG)

```
LOX1:      5'- AGG GAA GCT GTC TTT AGA ACC AAG C -3'
PNDEL1:    5'- TCC CAA GTC TCC CTC TCC AT        -3'
CHRRY SC1: 5'- CAC CCT TGG TCA CCT TCA GCT TGG   -3'
SDL2:      5'- CAC ACG TAT CTG GGG AAG GAA AGG   -3'
```

13. Oligo (reverse): Highlighted in magenta (e.g., ATCG)

```
SLD2:      5'- CAC ACG TAT CTG GGG AAG GAA AGG -3'
PNDEL2:    5'- CCA TCT GTT GTT TGC CCC TC      -3'
A2:        5'- ACC TTC AAG GAC CTG TGT CAT TCC -3'
```

CATATTGGCAGGACACACGGGTGTTTAGAGCAGCTGTCTGTAAGTTAGTTACCCAGAATCCATTGTTAG
GCCATGCTCTGTGCTGCATGTGCCCTATGGACTTCATGACCCACATTCAAATGAAATAAAGAGATTTCT
GTACCCAAAGAAAGTCCCTTTTTATCTATAAATCACAGCATCATATCAAACTAATATGGGAAGAATAAG
AATATAGGGCTTGAGGTATTTAGACGCTAGTGTAAGAAAAGGTGATTAGGGTCTGATCATATTGTATGA
ATATAGGGGGCATTAAATGCTATGTGAGGGCAATTTTGTAGTAAAGCATGAGAATTTAAAAAGCTATTG
TGGGAGAGGATTGAAAAGAAAGAATAGCAAGGGTGACTCTGGTATTTTGTATACATAGATGAAAACATT
ACAGTGAAACGTGCAACCATTTTAGAGTTGACATATATTAGTGAAAACAGAGGCTGACATTAAAAAAAA
ATCTCAACATGCAATTTTTGTTTCTGGGAAGTTAAAAATAAACTCACTATATATTCTATAGGGAAAGTC
AAATGCACAAGTTCAAGGAGAAATAGGAACCATTTGCATGATGCTACTGTTAGAAAAGAAGCTATGGCT
GCCTTTGAAGATCCAGCCATGGAGAGTGAGGAGCACACGAAGCCCCGCCCCTAGCTGAGAATGGGTGCT
GGCTGCTGGGGACAGTCAGTCTTCTTTGGTGTTATGTCCCTTGGCAGGCTGCCTGTGCTCCAGAAGATA
GCTCTGTGTGAAGATAGCTCTGTGCTGAATCGGGTACAAATGACACTAGCTGAACTCAGAGGGACATAA
GTGAAGGGCATGAAGTTAGGAGGGGGATGCTCAGAGCAAGGCTCTGGGGGGGAGATGGAGTGGAGCAGG
GACAGATAGATATAACCAGAATACACTCTATACATGTATGAAGTTTATAAGGAATGAATAAAGTATGTT
TTTGAAATAGAAAAAGATTGTGCATAAAGTGAATGCTAGTGTATATGATTCTATTATTTAGGTTATATT

2200 Smithtown Avenue, Ronkonkoma NY 11779 ● tel: (631) 468-8530 Ext.114 ● email: echen@genetargeting.com

TTATACATTCAAATGAATGAAATGGTAGTTTTTTGTTAATAGTCAAAAGTTACAGTCTGAGTATAGCCT
CTGTGACTATTTGTCTTAGTTAGGGTTTCTATAGACAATTTGAAGGTGTTTACCACGGACAAAGTGTAT
TCAGGAACCACTTTGTTTGGGTTGGCCATGAGACAGCTTGTAGTCATTTCATCTCTGATGAAATCAGGA
CAGGAACCTGGAGGCAGGGACTGAAGCAGAGAGTATAGGGGGGTGCTACTTACTGGCTTCCTCCGCAAA
GTTTGCTCAACTCACTTTCTAAAGCCATTGGGGCCACATTACTAGGGATGGCTCCTCCCACAGTGGGCT
GGGCCCTCCCTCCCACATCAATCACTGGTCAAGAAAATGCCCTATTGTTAGTCTGGTGGGAGGTTTCTC
AGCTGAGGCTCCATCTTCTCAAATGACTAACTGTGTCAAGCCGACAATCAAAACTAATGTGGAGACTAT
TTAAATATATGCGGAAAACTTAAATGCACACTTAAAAAAACACGTGAGAAGGCGCGTTGCTCTCTAAGC
TTTGGAAACAAAGCTATAATCTCTCTTGTGTTTTTAAAGCAGCCTTAGACTTTTGGTATTGCTCTTTCA
GTCAGAAATGAATGTTGGTGCTGTTGATTTATTGAATCCCTTTCCTGTCACGGCTCTAAATGTATTTTA
TTTCCCTTTTAGCTTCAGATGGAAAGACATACGATGCCTATATTCTTTATCCCAAGACCCTGGGAGAGG
GGTCCTTCTCAGACTTAGATACTTTTGTTTTTAAACTGTTGCCTGAGGTCTTGGAGGGACAGTTTGGAT
ACAAGCTGTTCATTTATGGAAGGGATGACTATGTTGGAGAAGGTATGTAAATGTTTCTTGTGAAGTTAG
TTGCTGAGTTCTGATTCTGGAAGGTTGGCACCATTGTACATATTGGAAGACTGAAGAATTATTAACTTT
CCCTTAGTCATGTTGGGCCAATGTTTAATTGACTTTTAGTATTCTGGAATGTAATGACTAGTTAGATCT
AATCATATGCAACTGCTTTAATAGACTAGGAGTATTATAGATCAATCAACCTAACAAGAAGTTTAAAAT
TTTTTGGCTGAGAGTTTTCCAGGTTCTAAGAGCAAAGTCCGTCTTTCGTGGGTCAGTTTTTGAGCTGGT
CTGTAAATTTTAATTGTCTTTGAATCTCAAGTAAGGCAAGGAAGAAGCAACAGGGGACATAAAAAGGAG
TCTGGGAAGAGCATCTTTGAGTGTTGAATTCAGGAAAGAATGTATTGATGGCCTCTTGCCTGACTGCAC
TGCACTCTGTGTCTTACAGATACCATCGAGGTTACTAATGAAAATGTAAAGAAAAGCAGGAGGCTGATT
ATCATTCTAGTGAGAGATATGGGAGGCTTCAGCTGGCTGGGCCAGTCATCTGAAGAGCAAATAGCCATA
TACAATGCTCTCATCCAGGAAGGAATTAAAATCGTCCTGCTTGAGTTGGAGAAAATCCAAGACTATGAG
AAAATGCCAGATTCTATTCAGTTCATTAAGCAGAAACACGGAGTCATTTGCTGGTCAGGAGACTTTCAA
GAAAGACCACAGTCTGCAAAGACCAGGTTCTGGAAAAACTTAAGATACCAGATGCCAGCCCAACGGAGA
TCACCATTGTCTAAACACCGCTTACTAACCCTGGATCCTGTGCGGGACACTAAGGAGAAACTGCCGGCA
GCAACACACTTACCACTCGGCTAGCATGGCAAAAGTGGGCAGGCCAAGAACTTCGGAATATCTCCCATC
ATAAGAGGCTGCAGCTGGGCTGTGCCTCCCAGTAAAACAGTCACGAACCAAACCTGTGCAGTCCCTTGT
TCCAGATCACCTGGAACTGGATTGGGAAGAGAACAGGACTTGGTGGCCAGGACCGCTCAGAGAGCCATG
GTTGCTCAGGGATGCTGCTCCGGGATGCTTGACTAACAGTCGAGGCAGTGAACTGGGTGTAGAAAGCGT
CAGGAAATGGCCACATGTGTGGATGGTTTAATTAGATTCTGTGGAGTCTCACAGTGGGATTGTGGTTGT
CTGAGGACACTTTGGGGGGTCGCTGTCCAAGAAGTGGCTCCCCAAAGTATAAGTGCGGGTGAGGTTTAC
TGATACCCCACAGTGGAAGGCATTTCTTCCAGATATTTTCCAGATACCTTGGCCGCCAAACTTCGTTTT
CAAACACTGTGTTTTATTGTGTAGACCTTGAAAACCACGGTTTCAGTGAAGAAGGATTCTGTAGGGTTC
ACTCTTCTGCAGTCACTTTAGCTTTCTGCAGAAGTCTAGATTTTAAATTAACTGTAGAAGGAAGTCAGA
CACCCACAGGTTTCTTCTAATCAAGCTTCCACCCTGCAATGGCTGTCGTGCCATCTTCCTTCTGTGTTT
TACTCGGCTCATTGCTTCTCTGGAGACAAATGCAATGCTTCCTACCCCCGTCCTGTCACAGGCAGTCTG
AGGACTCACCTTCACACGTGAGACTTCGCTCTTACCCCAGCCCCCACCGCTGGAGGGGTTTCAATCCCC
CACCCTGGGGCTCTTCTGCGCAGAAGCTGAAGTCTACGCTCCTCCCAATCTTTGGTCCCAGTGCCGCTG
TTCGCCTTCCTGCTGGTTGATTGCAGAACCACCTCTGAGACCTCTGTACCTGCTCTTGGAATGTCTCTG
TCTTTGCCCTGCCGCCCACTTCCCGTGCCTATGATTTGCTCTAGCATTTCTGCCACCTGACAAGGAGAG
GAGCTTGCTAAAGTTTAAAGTTTATTTCAACGCTAAGATCCCAACCTTCCGATCTCTGCCTACTCTCAG
ACTTGGCCAGCCCTCTTGCCTGTCATCCCAACCCTGCTGCTTCTCTGTAAAAGGTTGTTTCTTTACTAG
CAGAAATCAGTCTGCAGGGAGGGGTAGTGCGTGTATCTCACTGTTTTCCGTATTGGGAACAGATGGTAT
AGATTTGCCAAGGCCTTTATTTTAAAGTGTTCTTGGAAAATCGTATTTAAATTTGCAATTATTCTAATT
TGATATAGAGAGAAACTGACCTATTTAACAAATGGACTCTCTTAATTCTACTGGACCCAGGCCTTGAAC

TTTCTGGTTTCTGTCTCTGTGCTCTGACTTTTTGATTTCAGGTCTAGCCCTGTGCACTTACCTCAGTAC
TCAGTACTTACTCAGTACTAACTCAGTACTCGTGGAGGACACCCGACCCGGGTGTTCTTTGAGAAGCA
GCTGAGTGAGGCATGTTTTGACAGGAGGAACCCAGTTCTTTTCACTGTTGGTATTTATAGCACCTACCT
TCAGATTTTTCAGTATGATGTTTCTATTTTCCAATACTGGTTCCCTAACGGTCAACATTTCTTCTCCCA
ACTAAAAATGTAAGAAGAAAAAAAAATTTTTTTCTTTTTCTCTTTTTTTTTTTTTTTGTGGTACAGAAG
TTTTTTGTTTGGTTATTTGTTTGTTTGATTTTAACTAGATGACTGTCTTCAGAATAATCCTCACAGTTC
TGGAAGATTGAAAAACCAAGTTCACATGACAAGAACAAACCCGGTTTTAAGGGTTGAATGTAGGGGGAT
ACCATTCTGTCTTTTAGACTTTCAACATCTGCTATGGTGGAGCACAAATGTTATTTAAAAGCATCTTCT
CACCTACATAGAAGTAAACAAAACACACAATTTGGGATTTTGACTCTTCAGTTTTGACCTCACTTCTCC
TGGATCCAACCTGACAGGAGATGACTGGATTGCTGGGGTAGAGAAGGAAAGGCTGTCCCCGACAGCAGC
CAGAAGTCTGTGGGAGTGAGGTCATTCCTGACCCGTGATGCTTTCAAGGCTTGGTTTTTCTGATAATTA
TTTTTGCCTGTCATCCCAAAAACGTATTGACATGCGTGTATATTACCTGTGAGTGAGAGTTCTTAGAAA
CTTGGCCAGAGAGGATAAAGGGGAGAGCAGTGACAGAGAACCGCCGATCTTGTGACACCGGAGTCTGAG
GTGGAACAGAGCCAGTGTCAGGAAAGCCCCTCAGCAAGAAAGAAGAGTCATTGCTGGTTCCCAGGGGCA
GTCACAGAGGATGAAATACCACATCAAGGCAGTGGGAACTTCCTGCGACTTCTCCTGGTCCTCCCCAGA
AGTATCTGGTGTCAGCTGAAGACCATGTGTCCCCCAATACTTAGATTTCACCGTACTTTCTGATGGTGT
TTTTAAAAGGCCAAGTGTTGCAAAAGTTTGCACGTGCAACCGTATATTAATCGCCAGTGTTTAAAACTG
GTTTATCAGAATTTATATTTTTGTCTCTCCTGCCTTTTGAATTTTACTTATGACAGAGAAAGTATTGAG
CCATGACTTTTTAAAAATAATTTATATTTATACTGGTCAATAATGAAGGTTTTTTTTTATTATTAAAGA
ACTTGATTTTATAATTCTGCAGCAGATGCCAAATGTTATCTATCACAAACCACAAAATTTATGTACAGT
GCATATTATTGATCAATAAACTAAACTTGTTTTCCAATAAATTGTTAAAGCCTCTTTTGAGTATCGCTG
AAAGTTGGTTGCTCTGTCCTTTTTCCATCTTACATTCGTTCTAAGGCCTGATAGGAAGGGCCAGGCCTT
TTGCAGGGTGTTCTTGGAGTAGGGGAGCTGAGATCTGTGCTGGTTAGTTTTATGTCAGATTGACACATG
CTAGAGTCACGGGCAAGGAAGGAGCCTCAATTGACAAAATGCATCCATATTTCTGGGCTGTAGGCAGGC
CTGTAGAACATTTCTTTAATTAGTGATTGGTGGGGGAGGCCCAGCCCATAGTGAGTGGCGCTACTCCTG
AGCTGGTGGTCCTGAGTTCTACAAGAAAGCAGGCCGAACAAGCCATGCGGGAGCAACCCAGTAAGCAGC
AGCCCTCCATGGCCTCTGCATCAGCTCCTACCTTGTTTCTGTCCTGAGTTCTTCAGTGATGAACAGTGC
TGTGGAGGTATAAGGCAAAGAAACCTTTCTTCCGAAGTTGGTCATGGTGTTTATCATAACAATGATGAC
TCCAACTAAGACAAGGTATGAGGTTGATGGTCAAACAGGTAGTTCTGTGAACCCTGACTTCCCTTACTT
AGAGCCATCTGTTCCTTTGTTTATGGAGTAGATTAAGAGCTAGGCTGCTGCTCTGTGATAGACCAGTTG
CCTAGCTGAGGCCCTGGACTTGATCCTCAACACATCACTAAACTGATGATAAACAAATGTAGATCTTAT
ATTCTGTTATTGTTGCCATTCTAGCCTTTATGGGTAAATATCCATAGCTCTCAGACCAATAAGCAAGGT
GTCTGAAATATATGTACTATGAAGCGTTGAGTTCAGTGGCCTTCATTTTATTGAGTAAACCACTCAATA
AAAAGTTAAAATAGTAGGTATGCAGAAGTTTGCAGTCTGGTGTCTGGAAAACTTACTTTTCCTAAATTC
CTCACCCAAAGAATCCAATATGCCAGTGTGCTTTTGTTCTTCGACCAACAAATGTTTGTCTTAGCCATG
TGTTAGGTCAGATCTAACACTGTTAACACGTCACATATAATGTCTCTCTGTCAAATGCCGTTCCCAGCA
TATTTTGAACTAGCTTCTTTTGGAGATCGAGGTCTCTGGAATTATTTCTCCCTAAGAGTGCCACTTGCT
TTCAAAGTTGCAAATTATGAATTTCTCTCCAGTTGTTCTGAATGAGGGCAGAGCGCCTGGCTGCTTTAG
AGCCCAGCTTTCCTCTCTCTGGTAAGACTTCATATATATCCAGGTCACACAGGAAGCATCTCCATCCGG
TACCTGGGACTGAACTTCCAAATAGACCCTCTTAGTTTTAGCAGCTAGTGGACGATACATTGAGAAGTG
TTTTCACATTCACTAGAATAAAACGAGAAATGAATCCTTGCAGCAGCAAGGATACATGGGAGACAAGGC
CTCTGTGTGTAGGGGGAGGTGGGCGTTTGCGTGTGCGAGCACGTGTCCAAGTATATTCTGGAGAAGGAA
AACCTTACAGGGAATGGATGACACCATTTAAAATGAGCACCGGCCTTCCAAACAAAGGCATACTCCGCT
GGTTGCCGACTGCTCGGGGGTCAGCTGAGGAAGACTTGGCTGGCTTGGGCAGCTAACTCCTGTGTATTA
TTTCATCTTAAATCTGAAGATGAAACACTAACACCGCCTCTCTGAGCCAGGGAGCAAGCGGACCAGTCT

GGCCTGTCTTGCTAGACTGTGGGCTGGTAGGATCCTGTTGACACCTGCTAGGATCTCAGCAGGCTGAGA
GCCGTATCTATTCAACCTTTTTTAGGACCTTTTACCTAGAATGGGTTGCCGGTGTCATTTGGGCATTGT
TTTCTGGTCAAAACCTCTGGTTTTGACAGGTGCTTATTTCTGTCTGGCTCCTGATTTTTTCCCCCTTCT
CAACTTTCCTTGTCCAGCTTTGCCTGAGTCAGGTGCAGCGCCTTATTTACCTTGAGGGCTGTGGACAGT
GGCAGTCCCTCCTGTACACTTAGTCATTAACCGAGTTTCTGTTCTGCTTCAGTGCCCCTCAAGTCCTGT
GTTTCCTGCTTGATTCGATACATGTGGCTAATATCCAGACTCTAAAATGAACTGTAAAAACCAAACGCC
AAGGAAACGAGATGTCCAGTCGGCAAGCGGATGAATGATAGAACAGGCAGATTTCAAGGAAGGAAACAC
AAGGGGATGATGGTGGATATCTTTTTAAAGTGTACTACATCCCTAGTCATCAGGGGAAATGCAAATTAA
AACTACAATGAGATGTAGCCTCTTATGGTCGTCATAATGGCTATCACTAACAAACTCGACACCAGATGT
TAGGAAGATGCTAAGAGAAGCCTTACTCTACACGGGTGGCAGTGAAGCTTAAGACAGTCATTACGGAAA
TCAGTTTGGAGGTGCCTTGGAGGGCCCATGCTGGGTCATCCCCTGTCCCCTTGAGGTACCAGCTGTAGG
ACAGATCTAATATAGAATGAAGTTTAATTGGGGACATGGGATGGAAAGTTGAGAAGGGAGTAGAGACAG
AGAATGAGAGGGGACAGGGAAGGAGAAAGAGAGAGAGAGAGAGAGAGAGAGAGAGAGAGAGAGAGAGAG
AGAGAGAGAGATCAGAAAGAGAAAAGGGAGGCCAAAGAGTCCCTTTCATAGCAAGCCAGGACTACCAGG
CTGTTGCTAGGTAACTGTTGGATGGAGCCTAGAAGGAATGCTAACACTAGCCTTTAACACACAGAGGTT
TATATAACTATATTATATATACACATATATAAAGGAGCATGCATGCAGCTATAGTTTAATATGCCACTT
GGAGAGGAACAAAGGGTAAACATTGGGTGTGAACTGAGCAGGACTGAATGCAGCTGGGACAGGCCACGT
GAGTCATAAAAGAGAATTTAAGTGCCGGGCGTGGTGGCACACGCCTTTAGTCTCAGCATTTGGGAGGCA
GAGGCAGGCAGATTTCTGAGTTCAAGGCCAGCCAGGTCTACAAAGTGAGTTCCAGGCCAGCCAGGGCTA
TGTGAGTGAGAGAGAGAGAGAGAGAGAGAGAGAGAGAGAGAGAGAGAGAGAGAGAGGAGAGAGAGAGAG
AATGAATTTAAAGCTGATCGTCTAGTTTAATTACGTCATGGGGTTTTCATTTTTTGTGGTGGCTAAGAG
CACATCAATGTGACTGATAGTGAAGGTATAAAATCCACAGTGAGCCTCAGAACAGCTCTTGTATGAGGA
GAAGACCTCTCAGACAGGCAGACCTCGGGGCTGGGCCAGGCCTTGTGTGAGTTGCCATGTCAGTCTAGC
TGCGTGACTTTTTTTTTTCTAATTTGCAGATTCTTCATAATAAATTGATTAAAAGAAATAAAGCCAGTC
TCATATCCTTCACTGGATTTCAAAGCAGAGGAAATATGCCTGCTGTCAAGCTTATGTAGATATTCTAAG
AGTATTAAACTTGTGCCTAATGGAGAAATCGAGGAAAGAAGAAACTGGGATCCAGCGAGAGAGAAGGCT
ATGAAGGAAGAGTTGAATTCTGGCCTTCGGTTGCTTGTCTAGCCCTGTATGATTGACAGCATCCATGAC
ACAGGGAGGCAGGAGTGGTATCTGCTGTTGCTGTTTCATAGGCAGCCAGTACTGAGCCTTTCCTTTTGC
CAGCATGACAGCAACTGGCAGGCTGTCCACCAGGATCCCTCAGCCCTTGATAGCAGCCAGCATTACAGC
TACCAGACCTAGGTGTCCGGCACTGTTGCTTTTTGGCTTTGAAAGCTTGGGAGCTTCCTGGAACTCTCC
AGACACCAAAGCCTCAGTTTGTCTTCTCTGCCTTGGTATACTAGTCTTCCTCCATAAAAATTATCGGTC
AGCCATCCTTCCTCCTGCCCTGGCTGCTGTCTTGAACTTAGGGTGTCAGAATACAAGAATACTTGTTTT
AAAAAAAAAAGACAAAATTAAGGTATACGTGTGTGTGCATGCCTCTGTGTGTGTGTGCTTGTGTGCGCT
TGTGTGCATGCATGTGCGTGTACCCAAACAAGCTTTAGAGAAGGGGAAAATTACAGGAAATGGAACCTC
CCCCTCTCCTGTTCAGCAGTTAGACAACCAGGCACTTTTCCACTTTGGCAGTTTGGCCTGTCACTTGTC
ACTGCCCCTCAGAACTCCTCTTCCTGTCAGGCAGCTAGCTACCTTGCCCCTTGCTGAGAGAGACCACTG
GGGAGAAGCTAGTGTGACAATTGAAGAGAGGCAGAAGGGTGTCCCGACAGAGAGAGGCTGAGCAGAATG
CTTAGATAGCTGCAAAAGGGTGTTGGTACCAATCAGAGCACTTCACATGCATATGGAGGATTCTACAGA
CATTTACCACTTATATTCCAGCCATGGCTGTCTCTGTACCAGCCCACTGGGGTATGTGTTCCCTTTCTG
GCTCAGGCAGGAAAGAATGCAACATCCTTTGGAGTTCACTGAGGCCCAAAGCAGCAACAAATCTAGTAT
GAATTGTCTGAGACTGCCCGGCAGAGGCTACACCTTCAAGAGCCAGCACACTCCATTGAGAACTTGGGG
AGCTGCCTCAAGGCATCCACCAGAGAGTTCATGGCTCAGCAGCTAAAGGTACTTTCTACAAGCCAGGTG
ACCTGCACTCAATCCCTGGAACCCTTGTAAAGATGGAAGGAAAGAACCTGCCCCCCAAGGTTCTCTGAT
GTCCATATATGTGTCTTGGCACATGTATTCTTCACCCTAGAGTGTGCACTCTTGCGCATGCGTGCACAC
ACGTGTACACACACACACCACTATATGCCCTCACATATACATATACCCCTATATGCACACACACCCCTA

2200 Smithtown Avenue, Ronkonkoma NY 11779 ● tel: (631) 468-8530 Ext.114 ● email: echen@genetargeting.com

TATGCCCACAAATATCCTATATGCACACATACACCTATGTGCACACACATACCCCTATCTACACACACA
CATTCACATCCATACACATACAAAAACTTAGC***ACAAACCTTTGTCCATGCTGCTCTACTTGAAGAATGC***
TCTGTAAGTTGCTATTAAGGCTGCCAAATGGATAGCTTTTCTGTTTCTCTTGGCCCTCTGGATTTTCTT
TCCCCTAGTTTTTAGATAGGCAGAACCAAGCCACCCTTTCCAAAGTCACTTGCTCCTCTCCCTGATAGT
TTGCTTGGTGTGGTTACAGAAGGACAGTAGCTCTTCCGGTTCTTGGCTGACTACATTCCACAGAGACTG
TGCGGCTCTCTCACACACAGGGTTGCAGTGTTGGGCACACACCTAGAAATCACTGTGAGTGTGAGAGTC
AAAACCGATGTCCTTTGAAATGAATGCTTCTTTATTAGTTAAGTATTAATGTTTAAAAGCTTTACTTAC
AAGTACCTGGGCTATGGGACTGCCTTTGCTCCCGATTCATGCCAACCTTCATTAATGGCCAAGTGTGCT
GAGACAGGAGCCAGGTCCTCTAACTCCCAGATAACTAGTTACCCTTTCTAACCATCTTCTTTCTGCAGA
TGACATTGGAGAGGAATAAATGCAAGTCCTTTTATTTTTGATAAGGACATATGTCCGAGTTAAGTTTGA
AAAGGTAGCCCCAGGAGCATCAAGAGTAGATAAGGATTTAGGGTGTTAATGTACAAGCAGAATGAGTTG
CAGCACTGGTTGGCACCAGAACTTGCCAGCAGCTCCAGGGCCCTGTGACTAGGAGCCAGCACCACTTGG
AAAGTGTCAGGGCAGATGGCTTCCTGCTTGCCAGGCTGGAAAGACCAGGAGACATCAACTACTGACTAC
TGGCTGAGTATCCTGAAGATCTGTTTGCACCTCTACCTCTAGTTATATGTTTTGAAGAGAAGTGTCATC
GAGGAATCATAACGAGGTTAGATAGATGGACAGCCGTCTGGAAAACTAGCTTCCTCTTTCTATGGACCC
AGAGGAACAAGACTCCTGACGGAGGCCACACTGCCAGGCTTCCTTTTTCTAGAAGGGGAAATGATTTGC
TAATAGGACTTTAGTTTGTAGTGGGAGCTCTTGGAGGCAGTGGGTTTCTTCAAGCCCAGGCTAAAGGAC
TCCTGGATGTGTGCACTCGTGGCTGTGACTGTAAAGAATCACACAGTAATCATTAACTTCTGAGGCTCT
GTGGTGCTGGTTATCGTACCAGGCGGCACCCCATGTATTTGCCGCTCTACCCAACACATATCCAGATTT
TAGGTTCTACCCCAATGTATGGTTTGTGGGTCTCTAAATTTCATGTTAGACAGAAGTTGAGAAAGTGCT
GAGATTTATGTCATAACCATATAGGTCAAAACAGAAAGGCCAAAGGGAGTATCTTCCTCTGGTATCTGT
GGAGAGGTTGCCTGACTGAGCAACCAAGGCGCACATGCACATAGCCAACACAGATGAACACACATAATT
AAACATAGTCAAAGTAAAACTTAAAAAGTAGAAAGAGAGAGCTCTTGCCCCAGACATCTACCTGTTGGC
CCTAGAATATGGAGACGGACAGAGCTTCCTCTCCAAATCAGAAATACCTTGTCAGAGCTGGTAATGACC
TGGGGCCAGGGCCTTGGGGGGAGCTGTGACCTCAGATCCTGAGATCTCAACTCTTCTGACTGTATGGAG
TTATTGGTTATCAGTCTCAAGGCCATTGTATGCATGGCCTATATATAGTATTCTCCAGAAACCCTGTGT
TCCTCTCTTGCAACACTGTTTGATTAGCTTCCTGCTGACTTTGTCCCGTTGTCGGGTAGCTTCTTTGGA
CACCCCCATTTCTCCCCTGGAAGTTGTACATCAAATAATGCTGTACTTTTTAAATAAGCTTGCTTGGCT
CAAGCCATAGCTTGTACAAGACCTCCAGATCCCAAACTTTTTTTTTAAATCTTCTCTCTCTTCTATCCT
CTTTATCTTCTGATCTCCTGGTTCTCTCTCTCTCTCTCTCTCTCTCTCTCTCTCTCTCTCTCTCCCTCT
CTCCCTCTCTCCCTCTCTCTCTCTCTCTCTCTCTCTCTCTCTCTCCCCTGTTACTGAAGAAGGGCTTGCTG
GTCTAGGCCCTAACCCCCACTCTCTGTGTCTCAGGGGAACACAGCAGCCATGAGCTCAGTAGCACACAC
CGACTTGCCCTGCCTTTGTGTATTATTAGATCTACTCTTTCCCCATGCAACTCCCCCGCCCCCATGCCT
TCACCCTGCCATTCCTCTTCAGCTCTATGTGCACCTGGTTTGAAAACCTGTGCTGTTCCCTTAGTGCTT
TGCGCTTTAGGCTGTCTTTAGTGGTAAGGGTACCAATTCCGGGGTGAGGAGGAATTAGTGTGAGATTAT
ATTTTTCCTGATTTAAACATTGTAATATCTTTTAATTTTGCCTATAATAATGGCAGCCATTGTATTGTC
GTTCCCAGGCCCCATGTGATCTAGCCACACCTACTTCCATACTTCAGTTTACCCAGCTCTCCCCTCTGC
TGTGTGGCAATCACTCTCCCTTCATTTCAGTTTTTTTTTTTGGAGTGTCCCAAACTCGGTCCTTCAGAG
ACTTTGTACTGACGGTTCATTCTGTAGGGATGTAAATGGCTGTTGCAGAAGATCAGACTTTGGTCCCCA
GAACCCATATCAGGTAACTCACAAACACCTGTAATTCCAGCTCCAAGGGAGCCGACACCCTCTTCTGGA
CCCTATGGTCACCTGCAGTCACATGCACATAGCCAGTGCAGATGCACACACATAATTAAACATAGTAAA
AATAAAACGTAAAAAGTAGAAAGAGAGAGCTCTTGCCCCAGACATCTACCTGTTGGGCTCCAGGCCACT
GCTCAGGAGAAAGGCTGTGCCTTATCATATTCTTTCCACCTGTCTCCTAACTGTATTTCATATCTTATG
TGCTTCCTAATAGTTGAATATCTCTTTTAAAAAAATGTTATTTACCAGAAAATCATGGTGGCAAGCTGT
TGAAGGGCTCATGTCTCTAAGCTGTCTTCCCAACACTCAGTGTGCCATCGGACACATTCGGATGCCTTT

AATATTTGGCAAGAACATGGGGCATCAGAATCCTTCTCTAGAGAATGGTGTCATGGAATAATCAGTCAG
AGGTTTGCCAACGCAGACGAGGGCACACCTACCTAATGATCCAGGAGCTTCTCTCAGACGTGTAAGAGA
CATTGCCAGGATTGTCGCGGCAACATTATGTATAATAATTAGCAAAACGTAAAAAAAATGGGACAACCA
AAATGGCTAAGGGAGAGAGACACGTCGGTTGCTCATGCTCACAGGATGGGGCAAGCCTTTATTGAGAAG
GACCACACCTCAAATGTTTAAATCCCGCAAGTATAATGCTTTTATTAAAAAGTAAGTTATGTAAGTGCA
CAGTATAGTAAATTTTATACAGTGACTAGGAAATCTTATGTAAGAATATCTTACATTGCTTAAGGAATT
TCCTGCTTCTCCCTCTCTGCTCTCCACCCCCACACACCACTCAGACACACACAAAGGGAGGGGGAGGGG
AGAGAATAAGCACCAAGCTGAAGACTGGGAGTTTGGGTTTGGGCGGACAGAGACCGGAAGCTGGGAGAT
GATGGCTTCTGATGTTTGTTGCTTACGCGACACAGCTTGTTTGGAATTATGTGGCCCTTGACAACTGCG
TGGCACACACTTCAAGATACAAAAGTGAGAAGAAGAGGGGGGTCACCAGAGGAGACTGAAGTCAGTAAG
TTTCTACCAAAGCTGGGTTTCTTCTGGCCACTCCAAACAAAGCTGAAATTGTCCTTCAGACCTCTGTGA
GTACAGTCTCAGTTCCTTCCGTAGTTCTCTGGGAGGTTCTGCGTGCACACACGTGATAGTTTTGTGTTT
TTATTTTTAAAACTTCAATCAGTTTTGTCTTAATTTTATATATTACTGGGGTTTGGTGCGCACTAATTG
AACCCAGCTTCTTTTCACCGGCTTCATCCACCCACCCCTGTAATCTCTCTTCTACATCTTAGCTTCCTG
TTTGTTTAATTTCCGTATAGGTAGGACGTTTCTTCTGTTTCTCATTCCGCTTAACGTCGTGGTTTTTGT
TTTTGTTTTCCCTGAAAACTCACTATCCCCATATCTGTTCTGTATCCAGAACTAATGATTGATTCCTCA
TGCATCAGCAGACTTACTCAACACAGAGATCATCCCAGAATTGTAAGGCAGAGTGCTTCTGCCAAGGGA
CAGTACTCGGACAGACACGTGTACATCTTAACTTTGTGAACCTGGGACAGCTAGTCAGACTGACGCTGT
GGTTGTTTTCAAAGTCTTACAGTTTCTAATCCACAAAGACTCCGAGGGATGCCCACTAAGAAGCTGTTG
GTCTGACAGCGTTTGCCGCCACTGCTCTTCTCAGTGCCTAGTGGGGTCTCCAGTGTCTCTGGCGCAGGA
AGACAGGTCCGAGCAAGCATTACTTTATGGCAAGTTTAGACTGACTGTATCGGTGCTCAACCTGTCGGG
AGTCTGCTTTACCTAAGATCTGGCACGAGGACGGGGCTGGGGTGGGGTTAATGCCTTTTCTCAGAAGCG
CTTCCACCTCTGGAATAGTCCATTTTAGGACAGTGCTGGGTGTGTAAGAACCGCTCAGATAGTGTTTGG
AAAGGATATAATAATTTTTAGCGATGATCTGGAGCATATTTAAGCCGACATTCCTGATAGATGAACGTG
CTGGCCTGGGATCAGGAAAAAAAAGACTGAACGATTTTCGAAGGACGGATTATTTCTGTCCTGCTCCACT
TGGGGAAGGTGAGTCTGTTCCGCCCGCGGCGGAGAAGCGATGCCCAAGGTGCAGAGTGCGGTGACCAAC
CAGGATTCGGTCCCCAGAGTGGTTCCCGCTCTGCTCCACGAGACCCAGCGCTCTGAGACCCCATAGGCA
GGTGGAGCAAGCCCGGGGCAGCCGGAGCGGAGGGAGTTAGCGATCAATAGTCACTGGGGCGGCTAGAAG
ACTCCGACCTGGAAAGTGGGCCGGGACTTGGGTCAAACTCTGGGATTCCTGCTCTCCCTTGCCCTTTCC
AGGTGGGCCAGCACTTACCGCTCAGCCAGCGAGCACATAGGGTTACAGGGGAGCCCCTCCGACCCATGC
GATCCCAGATGCACGCTGGGCGGGTCCACAGTCTGCTCTGGCTGGGGTTCAGTCTCCTGGCCCTGGCTT
GATCCAAGTGCCAGCACATCTGGCCCTGTCTCCCAACTCCCCACCAGTCCCCTTCACAGGCCAGTCGGC
GTTTACTGGCTTCTGAAGGTCTGGTGTAATGGAGATGGGGAAAGGCTGGCCTTGGAACGGAGGGAGGCC
AAGTGGAGGGCTCCCCATGCAGAGGGGAAGGAAGGCCAGTGACCTGAACTGGGACTGACATTCTGGATT
CTCTAAAATTGTGTGCGGGAAGGTACCAGCCTCACTGCATCCCTTACTTCTACCATTGTCCTCTCTTTT
CCTTTGTAGCCTGAGATGGGGGTTACATCTTTGCTCTTCTGTGGGGTGTTTTTCCTGCTTCTGCTTTTC
GTGGCAGCAGGTAAGTTCTGCGGGTCTGTGAGTCCTTGTATCCCAGAGACCTGTTGCCTTGCCAGCAGC
ATCCTTTGTCATTCATCTCCGTGCCCCTGACTCACCCACTTGCAGGGGCATCAACTAAGCCCCAACCTT
GCTAGTCACTCTTTCCACCAGATAATCTGGCAGGCCGGTCAAAAAGGGAGCGGGGAGGGAGGGATAGGA
AAAGTTCATTCCCAACTTCTTGGAGGCTCAACCCCCAAATGTCACCTTGGAACTTTGGACTAACAGGTG
ATACAGAGAAGTCCTCAAACACTCGAGTGGACTCAGTTCCAATGCTTAAACAGATGGGCTACTAGTGTC
ATCTATTTCATATAAAGGGCTATTTTATGGTCCAAAACTACCAGAGAGTACAAGTTTCTAAGAAAACCC
AACTTGGTAGAAATGGGCTTTAAAGGGAAGCTGTCTTTAGAACCAAGCACTGTGGAACGAGGATACATC
TGACTGCAAGTATAGGCAGGAAAACGACCAGGTTGGTTCTATGTTGAGACAGAGACTAAGGGCAGAAGA
GTGAACAGATACAGACACAGGGGCCGTGGTTCTGTGGAGGTCCTGGGGAAGCCTTGTCCTCACTATAC

2200 Smithtown Avenue, Ronkonkoma NY 11779 ● tel: (631) 468-8530 Ext.114 ● email: echen@genetargeting.com

ATTTGAAGGTCTTAGCATTATGCTTCTAAATGACTGGGCTAGTTTGGGGAAACACCCCAAATAACTTCG
TATAATGTATGCTATACGAAGTTATGTACAAACTCCTAAATGCAATTTCACATTGACCAGAGCAAAATA
TTCCCTAAAAAGAATAATGACTCCAGTTTTCTGCCTTTATTTTATTTTATTTTATTTTATTTTATTTTA
TTTTATTGTTTTCCTTTCCTTCCCCAGATACGTGTGAGGACATTTTTATGCACAATGTGATAATTTCAG
AGGGCCAGCCTTTTCCTTTCAACTGCACATACCCGCCAGAAACAAACGGGGCAGTAAATCTGACATGGT
ACAAAACACCTAGCAAAAGCCCAGTATCTAACAACAGACACCTTAGAGTTCACCAGGACCAGACCTGGA
TCTTGTTTCTTCCATTGACACTGGAGGACTCCGGTATCTATCAGTGTGTTATAAGGTAAGTCCTTCATT
TAAAGTGGAACTAATTCCCAAGTCTCCCTCTCCATTTCTAAAGACAACTTTTTAAGAAAATGGGTATTA
TTGGCCTTTATTTTGGAGACTTGTTATATAGCCTAGACAAGTTTCCAACTGCTTTCTAAAGTACTGAAA
GGACAGGTGTGAACCACCACAACCAGCCGCTACACACCACAACCAGCTGCTACACAAATGCGTACGTTC
GTGGGATTGTGTCCGTGTCGCGAAGTTCCTATACTTTCTAGAGAATAGGAACTTCGTTGGTACCGTACG
CAATTGATAACTTCGTATAATGTATGCTATACGAACGGTACTAGCGCGCCTCGACTCTAGCCCCCTCGA
CTAGAGGATCGAGCCCCAGCTGGTTCTTTCCGCCTCAGAAGCCATAGAGCCCACCGCATCCCCAGCATG
CCTGCTATTGTCTTCCCAATCCTCCCCCTTGCTGTCCTGCCCCACCCCACCCCCCAGAATAGAATGACA
CCTACTCAGACAATGCGATGCAATTTCCTCATTTTATTAGGAAAGGACAGTGGGAGTGGCACCTTCCAG
GGTCAAGGAAGGCACGGGGGAGGGGCAAACAACAGATGGCTGGCAACTAGAAGGCACAGTCGAGGCTGA
TCAGCGAGCTCACTTGTACAGCTCGTCCATGCCGCCGGTGGAGTGGCGGCCCTCGGCGCGTTCGTACTG
TTCCACGATGGTGTAGTCCTCGTTGTGGGAGGTGATGTCCAACTTGATGTTGACGTTGTAGGCGCCGGG
CAGCTGCACGGGCTTCTTGGCCTTGTAGGTGGTCTTGACCTCAGCGTCGTAGTGGCCGCCGTCCTTCAG
CTTCAGCCTCTGCTTGATCTCGCCCTTCAGGGCGCCGTCCTCGGGGTACATCCGCTCGGAGGAGGCCTC
CCAGCCCATGGTCTTCTTCTGCATTACGGGGCCGTCGGAGGGGAAGTTGGTGCCGCGCAGCTTCACCTT
GTAGATGAACTCGCCGTCCTGCAGGGAGGAGTCCTGGGTCACGGTCACCACGCCGCCGTCCTCGAAGTT
CATCACGCGCTCCCACTTGAAGCCCTCGGGGAAGGACAGCTTCAAGTAGTCGGGGATGTCGGCGGGGTG
CTTCACGTAGGCCTTGGAGCCGTACATGAACTGAGGGGACAGGATGTCCCAGGCGAAGGGCAGGGGGCC
ACCCTTGGTCACCTTCAGCTTGGCGGTCTGGGTGCCCTCGTAGGGGCGGCCCTCGCCCTCGCCCTCGAT
CTCGAACTCGTGGCCGTTCACGGAGCCCTCCATGTGCACCTTGAAGCGCATGAACTCCTTGATGATGGC
CATGTTATCCTCCTCGCCCTTGCTCACCATAGGTCCAGGGTTCTCCTCCACGTCTCCAGCCTGCTTCAG
CAGGCTGAAGTTAGTAGCTCCGCTTCCGTCCTCACACGTATCTGGGGAAGGAAAGGAAAACAATAAAAT
AAAATAAAATAAAATAAAATAAAATAAAATAAAGGCAGAAAACTGGAGTCATTATTCTTTTTAGGGAAT
ATTTTGCTCTGGTCAATGTGAAATTGCATTTAGGAGTTTGTATAACTTCGTATAGCATACATTATACGA
ACGGTACGCGTAGGGCAGCCTTGAACTGGCAGGAGATGACGTCCAGTGAGCACTTTTGAAAGCAGAACA
GCTTTAGAAATAACTTTTGGAGCACAGATTGCACCATCTTCTTCATTGAAGTTTTAGCCCAGACCAGCT
TACAGAGAGAGGTGTTTTATCAATGAGATTGCTGCCATTTCCGAAGATGGCTAATGGTTTTGGAAAACT
GGCTGATCCAGACCCAACTAGATCATTCAAACACATCTGATAAGGAATCAGCTTAAGGCAGACATTTGA
AAGCTAGCAAATCTGGGAGATGTTCTAGTGTCTACATGAATCCATGTGCCAGAGGTAATGTTTTTGAGT
GGCAGTGGACTACTGAACTATTTCCATGGAAGACAATTTTGCTTGGAGAGTACTAGAGTCTGCTAGAAA
ATACGTGCCCCACCAAAGGCACTTCTTCCTGTCCCCAGTCTGTCTCTGTCTGTCTGTCTGTCTGTCTCT
TTCCCTGTCTCTGTCTTGCTCTGCTTGTCTCTCCCTAGTTCTCTCCCTTCCCTTCTCTTTTCCTTTTCA
GTTTGTAAAGATTTTCAAGAGTTCAATAAAACCCACCTGTAAAACTCATTTGGCCTGAGAATTAAAATT
CTCTGACTTTCCACATAAGCTTGACTGACAACAATGTATTATGTACTCTCTCTAAACCTTGCCTTTCCC
ACCTGTAAATTGGGTGTGTTCTGTACGCCTTTAAACTTTGTTCACCTGGAGCAGCTTTTAGTGATTAGG
TTCCATGCTGTCGACAGTTTTAAGGGATACAGGCAGTCTCACAGATCAGATGTGTATAAGTGTAGGTGA
CTTCTGTACCAGAAGTGAAGTGTAAGCACTGTATGGAGTCTTCAGATAAATAGTTCAAGAGGTTCATGG
TGGCCCTCAGTGGCTCCTGGGCCGTGCTGAATGAGACACCAGTGTCTACCCTGATGAATACCACTTATC
CTTGGTTAGTGCATGGTTTGAGGGTTGGTTATTCTTTACTTCATATGAGGCAGAGGGTTTGTGCAGTGT

2200 Smithtown Avenue, Ronkonkoma NY 11779 ● tel: (631) 468-8530 Ext.114 ● email: echen@genetargeting.com

CTAGAAAATAATAAGAAAAGATCTGAAAATAGCTGACTGAATTCTGAGACTGCTTAAGAGTTCATTGGC
TGCATTCTGCTGTGTTTCATTGTTATTTTGCTTGTTTTTGTTTTGTTTTGTTTTACAGGAATGCCCACA
ACTGCTACCAAATAGCTGTGAACCTAACCGTTTTAAAAAACCACTGGTGTGACTCTTCCATGGAGGGGA
GTCCCGTAAATTCACCAGATGTGTACCAGCAAATATTACCCATAGGAAAATCGGGCAGTCTGAATTGTC
ATCTCTACTTCCCAGAAAGTTGTGCTTTGGATTCAATAAAATGGTATAAGGTAAAACCAAACCCAAACC
CAAACCAAACAAAACCAAACCAACAACAATAACAAAACAACAATGAAATCCTCTATTGATAATGTTTTC
TCCTCTTATATAAAACCCACTTTCCCCACCAAGGGGAAAGCTTTCTGGATTCTCAGAAAGCCCCCTTCA
GGGCAGGTTTGGTATATAGTTGGTGCACCTGAGACAGGTGGTAGTAATTTTGATAATTACTTTAAACTT
ATGGTTGTTATTGTTTTATAATGTTGTTGTATCTTCTCTATACCCCAAATTATCCATTTTTCAAGTTCC
TGTTGTCTCTCTCTCTCTCTCTCTCTCTCTCTCTCTCTCTCTCTCTCTCTCTCTCTCTCTCTCTCTCCC
TCCCTCCCTCCCTCTCTCTTTCTCTCTCTTTCTTTTTCATTCACCTTGGTTTGTTAGAATGGCTCTGAA
ATCTGGTACTTTGAAAATTACAAATTACACAAATTCTTGCCTAAGAAAGACTCCTCAGGGCTATCTGTG
TTCTGTTACTCACCAAACCTTGTCCCCAGCCATGGCTGCTGCTATCAACTACTGCCACCCTCCTCCCGC
CACTCCCCACCTCAGTATTTGAAAACTCAGTTGTATCTTAGCCTCTTCTCTTTGAAATGTGAACAGTTT
CCCATGCTCCTCTTTCTTCATCCTGTGTTTGCTTTAACCTGGCCTTCTCTCCTCAAGGCTCAGCTAGAC
ACTGCCTGTCAGCTACCTTGAGTTTCCTTTGTGGCAGCCTTCCTAACACTGTGACCCTTCAATACAGTT
CCTCACATTGTGGTGACACCCCAACCATACAATTATTTTCATCGATACTTCATAACTGTAGTTTTGTTA
CTGTTAGAATTGTAAATAAATATCTGATATGTTTCTCCTGTGGGGGCTGTGACCCCCAGGTTGAGAACC
ACTGCTGTTTCATGGGTTTGGATTCACCTCCCCCACTTCGTCTGTGGCACGGGATTATTCAAACGTCTT
ACTTTTTCCTTTGCTGAATTTTGGTAGGATTTTGTTTCCTAGGGAATGACACAGGTCCTTGAAGGTTTC
ATTCACACTGGCAAATTATTTATCATCTCTTCTCTTCTTGTCTGTCTACTTCTGCAGAGGAGATCGAGT
TTGGTCCTCTTTACAGGGATTCTCTTCCTCTCTCTCCCTAACCTGGTATAAGCCCCTTTGCATGAGCAG
CTTGTCGTTCAAGATGAAGCCTTCTGTCTTCTCAGACAGCCTGCCTGGCACTTTGTGATCAGCTCGATC
TTCTTCGCGTTCATATCCTTTGCCGCCTTGTGTGGAGTGTGGACGGGAGCATCCTTACACCTGTCTTGT
GCGACTTTGACAGAGTGTGAGGTTCTCAAAGGTGCTTGATCACCACCCCTAGAATTGCCAGATACTAAA
GATGCAAATAAATAACTCACGGTTCCACGAATGTCTTGTAGTTCCACTTCCTGTCTTGTAGCTCCACTT
CCAGTCTTGTAGCTCTACTTCCTGTCTTGTAGCTCCACTTCCTGTCTTGTTTCTTAGTTCCCAACCCTT
TCAAGGAGTTTGTCAGTCTCCCCCCCGCCCCAACCCCCACAGTTGCTAACTCTCCGAGTTTGTCAGTCT
TATCGATGTTGTGGAAGTATTAACAGCAGGCCTTATAAATCCTCTTTTATTCGTTTGTTTGCTTCATCA
ATTTCTGTTCTTGTCTGGTTCTCCTAATCCCTTTGTTTGACTTTTGGTTTTAATTTGCTATTATTTTAT
GAACATTGTGAGATAGACTCTTCTCTCACTAAACCAAGTTTCTGACATTTAAGGTCGTGGCTTCCTTCT
AAGCCGCATCTTGCTTGACCTCTGCAGGTTTTGATCTATCATTCCATAAGCACCCGACTCAGGGCATTC
CAATTTTCCTTGTCATTTCTTCTTTGATTCTAAGTGTTTTTGGAGGACTTGTTAATTTGCAGATGTAGC
AATGTTTGGGTATTTTTAAAATTAACTTTTCTGTTTGTGATAGAAGGCAGTGTGACAGAGAGCATATTC
CAAATGATCTCAAAACTTTGAAATTTGAGGCTGGCTTTTTGAACCAACATATGTTTAACCTCTGTAAAT
GTGTCTTGTGTATTGAAGAGAATGTAAATACTGAAAGTTGGGCTTGTGGATTGTGTTATGCAGGCCAGT
GAGAGAAAAGCGTTAATATAAATTAGTGTGGTTATTCATTTCACTACACATCTGACATTGATAATGTCT
GCGCTATGCTTTTGAGATATTATTTACAGAAATATTTTAAATTACTCTGGCTCTTAACTCTTAACAACA
GTTCCCACATGATTATATATGACATATGTGTGCCCAGCTAGCATCTCTTAGAAAATTTATGAATTTTCT
TAGTGAAAATGATCTCATTTTCTGAAAATGTTTTTATTTCGTCCTACCTACGTTTTTTTATTTTTATGT
TTGTAAACTATTCTTCATGGATTGACATTTATTATTGTTCTGCACTTTCACACCATCATACAAGACTTC
AATATTTCTAGTATTTTTCAGGCTCTGTGGACTTTGTTATTATTCCCGTCTTTCTTCCCTTTTTGAAGG
TAATAGGCCTGAATCTCCGAAATCCTCAAAATTATTCTCCTTAACTTTGGAGTTTAGTAGTATAACTCT
GGTGAATGAAGATAGAGATTTCTTATCTATTTTGCAAATGTCTTGAAATATAGAGTATTGTGTCTTTCC
TCCATTGTTTCTTAGTTACTACTTTCTTCAGTGTTTGCTTCCTGGACTGCTTTTCTAAGTTTCCCATTT

ATTCTTGGGTATGTAAAACCATCTGTTAAATCCATTAGTTCACTTGAGTTTGTAATTTTCTTTCTTTCT
TTTATTTTCTTTCTTTCTTTCTTATTTTCTTTCTCTCTTTCTGTCTGTCTTTCTTTCTTCTTTTTTTTG
CAGGTGTTTGTTTCTAAGACTTTTATTTATGTGTTCTTTGATTAATTACAGTGTTAGAACTCAAACCCA
GGGCCTTGTACATACCAGGCAAGAGCTCTATCACTGAGCTACATCCTCAGCTTTACAAAAGTTCTTTTT
TTAAAAAAAATTTTTTTAAATTTTAAGATATTTTCTTTATTCACATTTCAAATGTTATCCCCTCTCCTA
GTTCCCCCTCCAAAAATGCCCTATCCCCTCCTCCCTCCCCCTGTTCCCCAACAAACCCACTCCCATTCC
TGGTCCTGGCATTCCCCTATACTGGGGCATAGAGCCTTCAGAGGACCAAAGGCCTCTCCTCCCATTGAT
GACTAACTAGGCCATCCTCTGCTACAAATATAGCTAGAGCCACATGTTCTACCATGTGTTTTCTTTGAT
TGGTTGTATAGTTCCAAGGAGCTCTGGGGGTACTGGTTAGTTCATTTTGATGTTCCTCCAATGGGGTTT
CAGACCCGTTCAGCTCCCTGGATACTTTCTCTGGCTCCTTCTTGGGGACCTTGTGCTCCGTCCAGTGGA
TGACTGTGAGCATCCACTTCTGTATTTGCCAGGCACAGGCAGAGCCTCACAGGAGACAGCTATATCAGT
GTCCGGTCAGCAAGCTCTTGTTGGCATCTGCCTAGTATCTGGGTTTGGTGGTTGTTTATAGGATTCATC
CCCAAGTGGGGCAGTATCTGGATGGTCCTTCCTTCAAGCTCTGCTCCAAACTTTGTCTCTGTAACTCCT
TCCATTGGTAATTTGTTCACCATTCTAAGGAGGAGCAAAGTATCCACACTTTGGTCTTCCTTCTTCTTG
AGTTTCATGTGTTTTGCAAATTGTATCTTGAATAGTCTAAGTTTCTGGGGTAATATCCACTTATCAGTG
AGTGCATATCATGTGTGTTCTTTTGTGATTGGGTTACCTCATTCAGGATGATATCCTCCAGATCCATCC
ATTTGCCTAAGAATTTAATAAATTCATTGTTTTTAATTGCTGAGTAGTACTCCATTGTGTAAATGTACC
ACATTTTCTGTATCCATTTTTCTGTTGAGGGACATCTGGGTTCTTTCTAGCTTCTGGCTATTATAAATA
AGGCTTCTGTGAACATAGTGGAGCATGTGTCCTTACTACAAGTTGGAGCATCTTCTGGGTATATGCCCA
GGAGAGGTATTGAAGGATCCTCCAGTAGTACTGTGACCAATTTTCTGAGGAACAGCCAAACTGATTTCC
AGAGTGGTTGCACCAGAATGCAATCTCACCAGAAATGGAGGAGTGTTCCTCTTTCTCAACATCCTCGCC
AGGATCTGCTGTCACCTGAATTTTTGATCTTAGCCACTCTGACTGGTGTAAGATGGAATCTCAGGGTTG
TTTTGATTTGCATTTCTCTGATGATTAAGAATGTTGAACTTTTTTTTCAGGTGCTTCTCAGCCATTCGG
TATTCCTTAGGTGAGAATTCTTTGTTTAGCTCTGTACCCAATTTTTAATAGGGTTATTTGATGTTTCTG
GAGTCCAATTTCTTGAGTTCTTTATATATATTGGATATTAGTCCCCTATCAGATTTAGGATTGGTAAAG
ATCCTTTCCCAATCTGTTGGTGGTCTTTTCGTCTTATTGACAGTGTCTTTTGCCTTACAGAAGCTTTGT
AATTTTATGAGGTCCCATTTGTCAATTCTTGATCTTATAGCACAAGCCATTGCTGTTCTGTTCAGGAAG
TTTTTTCCCCTGTTCCCATATCTTCAAGGCTTTTCCCCACTTTCTCCTCTATAAGTTTCAGTGTCTCTG
GTTTTATGTGGAGTTTCTTGATCCACTTAGACTTGAATTTTGTACAAGGAGGTAAGAATGGATCAATTC
GCATTCTTCTACATGATATCTGCCAGTTGAGCCAGCACCATTTGTTGAAAATGCTGTCTTTTTTTCACT
GGATGGTTTTAGCTCCCTTGACAAATATCAAGTGGCCATAGGTGTGTGGGTTCATTTTTGTGTCTTCAA
TTCTATTCCATTGATCTACCTGTCTGTCGCTGTACCATGCAGTTTTTTGTTTTTTGTTTTTTGTTTTTT
TTTTTTTTTTTTTTTACAATTGCTCTGTAGTACAGCTTGAGGTCAGGCATGGTGATTCCACCAGAGGTT
CTTTTATTGTTGAGAATAGTTTTTGGTATCCTAGGTTTTTTGTTTTTCCAGATTAATTTGCAAATTTTC
CTTTCTAACTCTGTGAAAAGTTGAGTTGGTATATTGATGGAGATTGCATTGAATCTGTAGATTGCTTTC
AGCAAGATAGCCATTTTAAATATTTTAATCCTGCCAATCCATGAGCATGGGAGATCTTTCCATCTTCTG
AAATCTTCTTCGATTTCTTTCTTCATAGACTTGAAGTTCTTATCATACAGATCTTTCACTTCCTTAGTT
AGAGTCACACCAAGGTATTTTATATTATTTGTGACTATTGTGAAGGGTGTTGTTTCTCTAATTTTTTTA
TCAGCCTGTTTATCCTTTGTGTAGAGAAAGGCCACTGATTTGTTTGCATGAATTTTATATTCAGCTACT
GCACTGAAGCTGTTTATCAGGTTTAGGAGTTCTCTGGTGGAATTTTTAGGGTCACTTATATATACTATC
ATATCATCTGCAAAGAGTGATATTTTGACTTCTTCCTTTTCAATTTGTATCCCCTTGATCTCCTTTTGT
TGTTGAATTGTTCTGGCTAGGACTTCAAGTACTATATTGAATAGGTATGGAGAAAGTGGGCAGCCCTGT
CTAGTCCCTGATTTTAGTGGGATTGCTTCCAGCTTCTCACCATTTACTTTGATGTTGGCTACTGGTTTG
CTGTAGATTGCTTTTATCATGTTTAAGTATGGCCTTGAATTCCTGATCTTTCCAAGACTTTTATCATG
AATGGGTGTTGAATTTTGTCAAATGCTCTCTCAGCATCTAACGAGATGATCATGCAGTTTTTGTCTTTG

AGTTTCTTTATATTGTGGATTACGTTGATGGATTTCTGTATATTAAACTATCCCTGCATCCCTGGATGG
AAGCTTACTTGATCATGATGGATGATCATTTTGACTTGTTCTTGGATTCAGTTTGTGAGAATTTTATTG
AATATTTTTGCATCGATATTCATAAGGGAAATTGGTCTGAAGTTCTCTTTCCTTTTTTGGGTCTTTGTG
TGGTTTAGGTATCAGAGTAATTGTGGCTTCATAGAATGAATTGGATAGAGTACCTTCTGCTTCTATTTT
GTGAAATTGTCTGAGGAGAATTTGAATTAGGTCTTCTTTGAAGGTCTGATAGAACTCTGTACTAAACTC
ATCTGTTCCTGGGCTTTTTTGGTTGGGAGACTATTAATGACTGCTTCTATTTCTTTAGGGTATATGGGG
CTATTTAGATCATTAATCTGATCCTGATTTAACATTGATACCTGGTATCTATCTAGAAAATTGTCCATT
TCATCCAGGTTTTCCAGTTTTGTTGAGTATAGCCTTTTTGTTGTGGGATATGATGATGTTTTGGATTTC
CTCAGGTTCTGTTGTTATGTCTCCCTTTTCATTTCTGATTTTGTTAATTATGATACTGTTCCTGTGCCC
TCTAGTTAGTCTGGCTAAGGGTTTATCTATCTTGTTGATTTTCTCAAAAAACCAGTTCCCAGTTTGGTT
GATTCTTTTTGTTTCTACTCGGTTGATTTCAGTCCTGGGTTTGATTATTTCCAGATGTCTACTTCTCTT
GGGTGAATTTGCTTCTGCTGTTCTAGAGCTTTTAGGTATACTGTCAAGCTGCTAGTGTACGCTCTCTCT
AGTTTCTTTTTGGCGGCACTCAGAGCTATGAGTTTTCCTCTTAGGATTGCTTTCATTGTGTCCCATAAG
TTTGGGTATGTTGTGGCTTCATTTTCCTTAAACTCTAGAAAGGCTTTAATTTCTTTCTTTCTTTCTTTC
TTTCTTTCTTTCTTTCTTTCTTTCTTTCTTTCTTTCTTCCTTCCTTCCTTCCTTCCTTCCTTCCTTCCT
TCCTTCCTTCCTTCCTTTCTTTCTTTCTTTCTTTCTTTCTTTCTTTCTTTCTTTCTTGACCAAGTTATC
ATTGAGTAGAGTGTTGTTCAGCTTCCACATCAACATTTTGCTGGGTATAGTAGCCTGGTCTGGCATTTG
TGTTCTCTTAGGGTCTGTATAACATCTGTCCAGGATCTTCTTAGTCTTTCATAGTCTCTGGTGAGAAGT
CTGGTGTAATTCTAATAGGTCTGCCTTTATATGTTACTTGATCTTTTTTTCCCTTACTGCTTTTAATAT
TCTATTTTCACTTAGTGCATTTGTTGTTCTGATTATTATGTGTCGGGAGGAATTTCTTTTCTGGTCCAG
TCTATTTGGAGTTCTGTAGGCTTCTTGTATGTTCATGGGCATCTCTCTCCTTAGGTTAGGGAAGTTTTC
TTCTATAATTTTGTTGAAGATATTTACTGGCCCTTTAAGTTGAAAGTCTTCATTCTCATATATACCTAT
TATCCGTAGGTTTGGTCTTTTCATTGTGTCCTCAATTTCCTGGATATTATGAGTGAGGATCTTTTTGCA
TTTTGCATTTTCTTTGATTGTTGTATCCATGTTTTCTATGGAATCTTCTGTACCTGAGATTCTCTCTTT
CATTTTTTTTGTATTCTGTTGCTGATGCTTGCATCTATGGTTCCTGATTTCTTTCCTAGGATTTCTATC
TCCAGAGTTGACACCCTTTGTGATTTCTTTATTGTTTCTACTTCCATTTTTAGATCCTGGATGGTTTTG
TTCAATTCTTTCACCTGTTTGGTAGTGTTTCCCTTTAGCTCTTTAAGGGATTTTTGTATTTCCTCTTTA
AGGGCTTCTAGCTGTTTACCTGTGTTCTCCTGTATTTCTTTAAGCGAGTCATTCATTTCCTTCTTAAAC
TTCTCCATCATCATCATGAGAAGTGAGTTTAGATCCATATCTTGCTTTTCCGGTGTGATGGTGTATCCA
GGACTTGCTATGGTGGGAGAATTTGGTTCTGATGATGCCTTGGTTTCTGTTGCTTCTGTTCTTATGCTT
GCCTCCTGCCATCTGATTATCTCAAGTGCTTGCAGAGCTCAATTTATGTGGTTGGAACCTGTTCTTCCT
ATAATCCTGGTTGATTCAGGACTTCTTAGAGTCTAGCTTTCTCTGTGATCCTGTGATTCCAGGATCCCG
TGAACCTGAGATTCTGGGTGTGTCAGAGTTCTTGGCAGTCAAGCTTCCTCTGAGACCCTGAGATCCTGT
TGTGACCAAGCTCCTGTTATCCTGGTATCCTGAAATCCTAAGATCCTGGGCATGTTACAGCGCCTGGAA
CTGGTGTTTCCTCTAGGGAGCATGGGGCTGTCTGGTGTTTTTGAAACCAAGGTATAACAGCACTGATCA
GAAGGAACCTGAGATGCTGGTCAAGCAGGGCTCCTGTATCCCTGCTCCTGCTGTCACAGGCCCATCATG
ATTGGTTTGGAACAGATGTTGTATTCTACTCACCAGTGATCCTAAGGTCCTGGGCGTGCTAGGGTGCCT
GCGGCTTGGAGAGTCCTCTGAGGACTGTCCGTCGAGTTCGTTCCCAAAGTGGCCCAGAGCTGGCGCCGA
CTGGAAGGGCACTATTATAATTTTCAATCCTCTGTAAAATTTTTGTTTTTTTCTATTTTGCATGTGTGT
ATTGTGGATATTTTTGCATGTGTGTGGGCATGCATGCAAGTGTGTACAGCAAGTTGGTGTCTGTCCAGA
TTCATGCTCGCTTGCTCTTATACGTTATTCTTGAGGGAAAGTCTCTTCATTAAACCCAGCACTTAACTA
TGCGGCCAACCTTACTAGCAAGCTAACACTGTGGATTCCCTGTCTCTGCCTCCTGAAGCTAGAATTACA
GGTGGTGGCCATGCCTACCTGGCACTTAGCTGGGTTTCTGCAGATCCAAATGCTGGCCCTCAAGTTTGC
AGAGCAAGCACTTTCAGCACTAAGCCAGCTATACAGCCCTGATCTGTAATTTGTAATTGTCATTTTCAT
AACTTATCTAGATATGGAGAAAGTCACCTACATTGCTGTTAAACTCCTGGTCTTCAGGAATGGCCCATT

2200 Smithtown Avenue, Ronkonkoma NY 11779 ● tel: (631) 468-8530 Ext.114 ● email: echen@genetargeting.com

TTAACCCTCAGACACCCAAGTGACCATCTTTCTCTCTTGCTGATAATGTTTCAGATATTCATTCTTCTG
CCACCATTTCCGGAGATTAGACTTCCATCTGCTCACTGATTCCACCTCCATGAGACTTTTACATCCAGT
GTGTATTAGTTAGGCTGTCCTGGGATATTTTGCATCTGAAAAAGATGCTGTACTTGCCGAAGTGGGATT
AGAACAGGAGTCAGAATGTGTGCTGATGAATGACAGTATCTTCATAGCATTAGCTTGTGAGCTATGAGT
AAATAAAAGATTTACATTTGCATGGTTCCCAGAGCTTAGATTATTTAAAACCTGCATTGGTTACATGCA
CTACAGATAGCTTCAGAAGTACCCAAATGTGTTGGTGTCACATAAATAAAAGAATATTCACACTTACCC
TTGGATTCATTTGATCAAGAACAAACTCAAGTTTGAATTTTTTTTCTCTCCCTGTGTCTCTGTCCATCT
GTCTGTCTTAATTTGAGGTTTGGGGGTTGTCATTATTCTATCGGTTGCTGAGTCCTTGTGTCTGTATCT
ACCTGTAGCCATTTGGTGGCTTATTGTCTCTTTCTTTGTGTCCCTAACAAATTGTTTTGCTCTGTATTG
ACTGAACATTATAGCATCACATGGGATGGAATGAAAGCCACTTTACATAAATTTTTACCAGAGTTTAGC
AAGGGATTAGGTCACTGACTCCAGCTGACCCCAAAGCACTCAGTTTTAAAAACATACTATTTTCAAGTT
ATAACTGTATGTTGTACCCAATATTTATGGTAGAAGTTTGCATACAAGATCTTTTTATACCTGTATCAT
GTTTTCAGGGTTGACTAGCATAACACAAGTACATGCATACATTGCTCTTTGCATCAGGAGAGTGGCATA
GTACATAATTTAAAATTAGAGATATGTACTAACTTATGTTGTGACTGATACATGGGAAATCCAAGGTTA
GTGGGGTTGACTGTTATGTTATTCTCTTAAAGTAGATTAAAAATCAGGCTTAGGGAACAGTATGACATC
AGGATAAGTCTTAAGACATCTCAAGGGGCATTAGCAACTTGACTATATGGTCCAACAAAAGTTCCAGTC
TCTTAGAAATGTGATCTATTTTCGTAATAATGCATCTCTATAACAATCTAACTGGAAAACGATTAATGG
CTGCCATTTTTATTATTTCTGTTTAAATCTTGATAATGAGATATCCCCCAAAAATAAAAGTTAAGCCAG
TGGGAAGTTTGTACCCTCCAGTAGATTGGGAAAATGGCACTTCCAGTCACTGCAAGGGGCAGGATGATC
TTTATACAGCAAGGTTAGTGTTAATGTGATGGCTCATGTGGATAATTGGAAGAAACCAATGAGTCAGAG
GTGGGATTGCTCAACTATGAAGCATGGCACAGAATGTGCTTAGAAAGGGACTCTCCAGTTTAGCCTACA
GGAGAGAGGTTTTTGGAATCTAAGAATTTGAGCTTTGAGACCTTGAACCCCGAAGAAGTAGACCATGAA
ACTCATACAACAGCAAACCAAAGACAAGCCCCAAACCACAAACTACAGATAGAAGACACAGGGTGGGAA
GAGGAGAACTCAGCTGTGAGTTTGCTTTTGGTCAAATCAATCCAAGGATTAAGTGTTGAGTTTTGCCCA
TTAATGTAACAGACATGTTTTGGTACCTTTGAGATTAGTTGTAATGTGTCTAATGGATATAGACTGTAA
ATAATCTCTAGGTGCCACATAAATTTATATGTCTTATCACTCCCCCATTTTATAGAGCATATCTATGAA
AATAACTAACACTCCACACACTCATTTGAATGCCTCTTGTATTCAAAGTGCCGATTTTATTGTGGTGCC
TTATCCACCCAACCCCTAATAGTAGGTCCCATCTACTACTGGTGAGCAATTGTCAAATGGTAACTAGAG
TTTCCTTAAAGAGTATCTGTTGGAGCAGTGTGTAACACATTTAAGAAGACAAGTTGTACTTTGTAAATA
AGCATTCTGGCATTACTTATGGATTGCCCATGCAGAGTGGATTTATTCCTCTTTGCTGAGGATGCACTC
ATCTGTGACTAAGTGATAAGGGGATGTAACACGGAGTTACTTACATTTTCCTTGAACTGGGGTTTTATC
TGTTTTAACTATGAAACTGTGATCTTCATGAGGACAGTAAATGCATCTAATTTCTGCATGGCCAAAGCC
TCTCTCTGTGCTGGAAAGATAGGAACTCAAAAATTCAAATAAAAAACAGACTCAAGCTGGGCGTGGTGG
CCCACGCCTTTAATCCCAGCACTCAGGAGGCAGAGGCAGGTGGATTTCTGAGTTCGAGGCCAGCCTGGT
CTACAGAGTGAGTTCCAGGACAGCCAGGGCTACACAGAGAAACCCTGTCTCGAAAAAAACCAAAAAAAA
AAAAAAAAAAAAGAAAGAAAGAATGAAAGGAAGAAAGGAAGGAAGGAAGGAAGAAAGAAAAACAGACTC
AACTAGCCAGGCAGTCTCAGTGTGGAGTGAGCGCTGCAAAGGAACAGGTTTGAGCAAGAGTCCGGAAGC
AGGTGTGTGAGGTGAAGGCGGTTAGCTTGATACGGTGAGTTACTGGTGAGGAAGGTGAATTGAAGGCAG
CTCACTTGGCAATATGTTTGTCTTGGAAACATGAGGACCTTGATTGCTCCCCATAAACACATTTTAAAA
AGCTGGGTGTGGTGGTGCACAGGGGACTGGCGGATCCTTAGGACTTGTTGGCCAACCAGTTCTGACAAG
TCAGTGCTGTCTCAAAATAAAAAGTGGAAAGAGACAGAGGAAAACACCTCATGTCTAGGTCTAGTTTAC
ACACAACACCACACACCCCCCACACACACACACATAGACACACATACCCCACACACATAGACACACATA
TACTCACACATACACACACACACACACACACACACACACACACACACAAATACATCACCACACACAGAC
ACACACAGACATGCATACAGATGTACTCACACATACACACATACAACACAAACACACATACACACAACA
CACACCACACATACATATCACACACATACATATACACTCACACATATACAAATATACATTCACTCACAC

ingenious targeting laboratory

ACAGCCACACACAGCCACACTCACACAGCCACACAACACAAACATACAGACACACATATAACACAGATA
CACACACACACACAAACACACACACACAGGAATGGCATGTGAGCCTCTTCTAAGACACACAAATATGAG
CCTTTCATTTTCTGTGGTTGCTGTAATGACAAGAGAGAAAGGCATTCTACAGATTGGTAGTTGCTTGAG
CCCTGAAGAAAGTTCTATGAGGTTTTCTCGTTATAGACTGATGTACGTGGGTTCTTTTCTCTCATTCAG
GCCTCCAATTAGTTGGTATACACTGGATTCTGTAAAGCAAACTCTCATCCATGGAGAGTCGAGTCTCTT
GTGTGGTTTAAACAGTCATTATCCAACTCACCGGGGCTTGCAATCCTGACAAAGGCGCTTGGCATCCTG
GGGTAGGAGTCCACTGGGGGAGGTGGCCTTGAGGTGGAGAAGAATATTCTTCATCTGCAGCCTTCCTCC
ATGAAATCTTTGCAGTTCTTCTCAGCTCCCATTAGACTTAGTGTTTGTATCACCCATTTTAGTGTGTAT
CCTTACCAAGTCTTCTTGAGATAGATATAAATAGAAATAAACAGATCTCTCTCTCTCTCTCTCTCTCTC
TCTCTCTCTCTCTCTCTCTCTGTGTGTGTGTGTGTGTGTGTGTGTGAGAGAGATCTATCTATTTACCTA
TCTATCTTATCTCTCTCTGTGTGTGTGAGACACACACACACACACACACACACAGACACACACACACAC
ACACACACACACACACACACACACACAGAGAGAGAGAGAGAGAGAGAGAGAGAGAGAGAGAGAGAAAGA
GTAAGTGATGAGCATCTACCCTCCCTTTCTCTCTCTATGTTGATTGTCAGCTTGAATTGATTGAGAAGT
GCCAAAGAATTTAAAGAGAAACTCTAGGTGTGTCTGTCAGGGTATTTCCAGTGAAGCCTAATAAGAGGA
AAATACCTGCCCTGCATGTGGGTGGCACCATCCCATAGTCTGTGCTCCAGATGAAACAAATAGGCAAAA
GAGAAAGCGGATTACTGCTGTCATTCTTTCTAATCCTGGCCTCCATGGTATGTTCTCCTGTGCCCCACC
CCATACTTCTCATGATGGACAAGTACATTAAATCTCGTCTTCTTTAAGTTGTTCATGTCAAGTCTTTTT
GTTAAAGTGACAAATCGCTACTAGCACAGTAACCTCCATCTTTTTGTCCATTAACCTCACTATCCTACT
ATCTGTCCATCCATCCATCCATCCATCCATCCATCCATCTATCCATGCATCTATCATCTATTCTCTCTC
TCTCTTTCATCTATTACCTATCTGTCATTCTATGTCTGTATATCATCTATCTAATCTATCTATCATCTC
TGTCTGTCTGTCTGTCTGTTTATCTATATTATGCATTGTCTTCTGGTTAGATGGTCAATATCTAAGATT
TAGGTTGTAAGTGACTCTGTAGATATTGGGCAGTGGTTCTTCAATCTTCCTGAGGCTATGACCCTTTAA
TACAGTTGCTCATGTTGTGATAACCTCCACCTATACAATTAGTTTGCTGCTCCTCCACAACTATAATTT
TGCTACTGTTATAGATCATATGAAATGTAAATATCTGACATGTGACCATGAAGGGGCTGCAACCCACAG
GTTGAGGAACAGTGATATAGACACATTTCCGCACAGTTCATAGGTTAATGCTGACAGAGAGACTAGAAT
TTGCTAGCACTCATTCCTTTCTTGTGTGTGTGCCTGTAGGGTTGTGAAGAGATTAAAGCGGGGAAAAAG
TACAGCCCTTCAGGAGCAAAGCTTCTTGTGAACAACGTTGCTGTGGAGGACGGCGGGAGCTATGCGTGC
TCAGCCAGACTGACTCACTTGGGGAGACACTTCACCATTAGAAACTACATTGCTGTGAACACCAGTAAG
GTTTTGTTTTTGTTTTCAAATATGGTTGCTTGGTTCTCAGCTTTATGGGAGCCAGATGGCTTCCTGCTT
CAAGTTTCAGAGATTGGATATTGATTGTACAGAGCCCCCCCCCCAGCCATGCTGGTGATAGATCAGGG
GGCCAGAGAGAGGGGCCAATGGGCACAACCAGCTAGGCACAACCAGCTCACTTTTCTCTCCTTTAAAAA
CACAATATTGAAAACTCTTGGCTGGAGAACATTGAAAGAGATCATGTAAAAGGGGGGAACTTCCTGGTT
GCCTACCAAAGTGTCAGCGTCCAGCACAATGTAGACAGTACTAATTCTACCTTCATTTCCCATCATCTC
TGCTGTTTTAGTGTCGTGCTGG
