# Supplementary material for: Floxed Il1rl2 Locus with mCherry Reporter Element Reveals Distinct Expression Patterns of the IL-36 Receptor in Barrier Tissues
Source: Cells. 2024 May 6;13(9):787. doi: 10.3390/cells13090787 (PMC11083296; doi:10.3390/cells13090787)
Supplement: Supplementary file 1 [file Cover Letter 040824.pdf › Cover Letter 040824.pdf]

TEMPLE UNIVERSITY
Lewis Katz School of Medicine

Liselotte E. Jensen, Ph.D.
Associate Professor
Department of Microbiology, Immunology, and Inflammation

April 8, 2024

Editors of Cells

Dear Editors of Cells

Please find enclosed our manuscript ‘Floxed *Il1rl2* locus with mCherry reporter element reveals distinct expression patterns of the IL-36 receptor in barrier tissues’ by Noppradat Tongmuang, Kathy Q. Cai, Jiahui An, Mariah Novy and Liselotte E. Jensen for consideration for publication in Cells.

The submission of this manuscript to Cells has been discussed with Section Managing Editor Beth Xu.

We confirm that neither the manuscript nor any parts of its content are currently under consideration or published in another journal.

All authors have approved the manuscript and agree with its submission to Cells.

The IL-36 cytokines are related to IL-1 but are far less well understood. Yet, evidence is mounting that the IL-36s are involved in a diverse range of inflammatory diseases from psoriasis in the skin, asthma in the lungs to inflammatory bowel disease in the intestines. The receptor for the IL-36 cytokines is IL-1RL2. In this manuscript we describe the development of a new transgenic mouse strain with a floxed *Il1lr2* gene locus. The targeting strategy included a reporter gene that can be used to confirm recombination and to track cells expressing *Il1rl2.* We here used this reporter to identify which cells express the IL-36 receptor in a range of barrier tissues. We examined both tissues in which a function for IL-36 has been demonstrated and tissues that have not previously been studied in the context of IL-36. With respect to organs which have previously been studied, several **surprising findings** were made, e.g., 1) the **distinct expression of the receptor in the air conducting epithelium of the lungs**, but not the gas exchanging alveolar epithelium, 2) **lack of the receptor in intestinal epithelium,** and **absence of the receptor in fibroblasts**. In addition, we **for the first time demonstrate potent expression of the receptor in the epithelial cells of the tongue, trachea, and esophagus**. This identifies these tissues as organs for which a function for IL-36 signaling has yet to be identified. The described mouse strain will be a useful tool for identifying such and other functions.

Our findings should be of interest to not only investigators already in the IL-36 field, but also those from other fields of study with potential interest in starting IL-36 research. As such, we genuinely believe this manuscript will be of interest to a wide and diverse range of readers of Cells.

Yours sincerely

Liselotte E. Jensen, Ph.D.
Associate Professor

1158 MERB • 3500 N Broad Street • Philadelphia • PA 19140
Tel: 215-707-8144 • Fax: 215-707-7788 • Email: liselott@temple.edu
